# Highly Pathogenic Avian Influenza H5N1 in South America, 2022–2025: Spread, Affected Species, and Southward Expansion into the Antarctic Region

**DOI:** 10.3390/v18070764

**Published:** 2026-07-12

**Authors:** Fernanda Sánchez-Rodríguez, Constanza Diaz-Gavidia, Soledad Ruíz, Pedro Jimenez-Bluhm

**Affiliations:** 1 One Health Institute & PhD Program in Conservation Medicine, Faculty of Life Sciences, Universidad Andres Bello, Santiago 8370251, Chile; f.snchezrodrguez@uandresbello.edu; 2 Escuela de Medicina Veterinaria, Facultad de Medicina, Facultad de Ciencias Biológicas y Facultad de Agronomía y Sistemas Naturales, Pontificia Universidad Católica de Chile, Santiago 7820436, Chile; constanza.diaz@uc.cl; 3 SENTINET, Santiago, Chile; 4 Escuela de Medicina Veterinaria, Facultad de Recursos Naturales y Medicina Veterinaria, Universidad Santo Tomás, Santiago 8370003, Chile; soledadruizph@santotomas.cl; 5 Centro Bahía Lomas, Facultad de Ciencias, Universidad Santo Tomás, Puerto Montt 5400000, Chile

**Keywords:** South America, Antarctic, phylogeny, highly pathogenic avian influenza (HPAI), H5N1, marine mammal, wild bird, poultry

## Abstract

The H5N1 highly pathogenic avian influenza (HPAI) virus has caused severe global losses, reaching South America in 2022 and Antarctica in 2024. Here, we synthesize outbreak reports submitted to the World Organization for Animal Health by South American countries and overseas territories in this continent, and document the virus’s unprecedented expansion into Antarctica, affecting wild birds, wild mammals, and domestic poultry. Phylogenetic and time-calibrated Bayesian analyses were performed on available genomic sequences. Over 6 million domestic birds were lost, mostly from commercial operations. Of the 11 South American countries and overseas territories that reported H5N1 to WOAH, 10 reported infections in wild birds, spanning 104 species, 59.62% of which are migratory and predominantly non-trans-equatorial. Marine mammal outbreaks followed wild bird detections, with the South American sea lion (*Otaria flavescens*) being the most reported species. Several Antarctic bird species with migratory behavior were also reported in South America. Genomic analyses revealed multiple introduction events, regional viral diversification, and patterns consistent with repeated cross-species spillover events. These findings highlight H5N1’s extensive ecological reach in the Southern Hemisphere and underscore the urgent need for a One Health approach that strengthens wildlife and backyard-poultry surveillance, alongside coordinated regional action to control and prevent further HPAI spread.

## 1. Introduction

Influenza A virus (IAV) is a member of the Orthomyxoviridae family and is a highly adaptable pathogen with a broad host range and significant potential for cross-species transmission [1,2]. It is primarily maintained in nature by aquatic wild birds, which serve as reservoirs for diverse hemagglutinin (HA) and neuraminidase (NA) subtype combinations [3]. Influenza A viruses are classified based on variations in these surface proteins. To date, 17 distinct antigenic variants of the HA and 9 different NA subtypes have been identified in avian species [4]. In addition to antigenic diversity, IAVs are categorized by their pathogenic phenotypes in poultry. Although many strains “of avian influenza” are classified as low-pathogenicity (LPAI) and typically cause mild or no clinical signs, they may still contribute to disease under certain environmental conditions or in combination with secondary infections [5]. In contrast, highly pathogenic avian influenza viruses (HPAI) can cause mortality rates approaching 100% in chickens, a phenotype that has so far only been observed in H5 and H7 subtypes [6]. Genetic mutations or reassortment events can transform LPAI into HPAI, resulting in severe disease in poultry, wild birds, and occasionally mammals [7]. A key molecular marker of this transition is the acquisition of a polybasic cleavage site in the precursor HA protein (HA0), which permits cleavage by ubiquitous host proteases rather than being limited to trypsin-like enzymes in specific anatomical regions. This enables HPAI to replicate systemically, contributing to its heightened virulence [8]. The polybasic cleavage site can arise through mechanisms such as gene insertion, substitution, or recombination with other viral gene segments [6,9,10]. Beyond point mutations, the segmented genome of IAV enables genetic reassortment during coinfections, producing novel viruses without pre-existing population immunity [11]. Although the low-fidelity RNA-dependent RNA polymerase contributes to genetic variability by introducing frequent mutations, viral adaptability, transmissibility, and pathogenicity emerge from a combination of viral and host factors rather than polymerase activity alone [12,13]. Similarly, long-distance spread and host switching are influenced not only by viral molecular traits but also by ecological and environmental processes, including interactions among wild species and opportunities for cross-species exposure.

The emergence of HPAI H5N1 clade 2.3.4.4b, which has recently spread across multiple continents, including Asia, the Middle East, Europe, and the Americas, originated from the Gs/Gd lineage of HPAIs first detected in Guangdong in 1996. This lineage has diversified through reassortment with LPAI viruses circulating in wild birds, giving rise to multiple subtypes and clades [14,15,16,17]. Clade 2.3.4.4b emerged in 2016 in wild birds in China and along the Russia–Mongolia border and subsequently spread across South Asia, Europe, Africa, and the Americas, causing major intercontinental epizootic events [17,18,19]. Its ongoing circulation along migratory flyways has led to the emergence of H5N1 as the dominant subtype in Europe and the Americas since 2020, highlighting the virus’s high adaptability, broad host range, and potential for zoonotic transmission [14,19].

Migratory birds then introduced the virus to the American High Arctic in late 2021, first detected in Newfoundland, Canada, followed by a spread along the U.S. east coast and into the Americas, causing widespread deaths in wild birds and mammals [20]. Over time, both the number of wild mammal species infected with H5N1 and the virus’s geographic range have expanded markedly. Before 2020, infections in wild mammals were confined to terrestrial and semi-aquatic species, with no cases reported in the Americas. Since 2020, however, aquatic mammals now account for a significant proportion of reported cases worldwide [21].

HPAI has affected many countries worldwide, and although South America has not been an exception, the number of outbreaks reported in this region has been comparatively lower, and only a limited number of distinct viral strains have been detected. To date, only two HPAI subtypes have been identified in South America: H7N3 in commercial broiler chicken in Chile in 2002 [22], and currently H5N1 clade 2.3.4.4b in domestic and wild birds, as well as wild mammals, which began circulating in South American countries since the second half of 2022 [23]. The latter has expanded its geographic range and host spectrum, including infections in multiple wild mammal species, with evidence of mammal-to-mammal transmission reported in South America, raising concerns about its ecological impact, potential for reassortment, and risk of zoonotic spillover [24]. While much of the early global focus has centered on outbreaks in Europe, Asia, and North America, the progressive southward dissemination of H5N1 into South America and the Antarctic region has emerged as a new frontier in the global epidemiology of HPAIV [25,26].

In this manuscript, we present an exhaustive review of publicly available data to characterize the impact of highly pathogenic avian influenza in South America and the Antarctic region. This includes a comprehensive assessment of reported outbreaks, affected species, and the geographic extent of viral spread across the continent. Additionally, we analyze publicly available viral genome sequences to provide insight into the genetic diversity and evolutionary dynamics of circulating strains. By combining epidemiological and genomic information, this study aims to provide an integrated regional perspective for public health officials, wildlife conservation managers, and policymakers. Although Kuiken et al. (2025) [27] recently reviewed the spread of HPAI H5N1 in South America and Antarctica, their study was limited to South American outbreaks through 2023 and Antarctic outbreaks through 2024, without incorporating data from domestic birds or phylogenetic analyses. Therefore, our study provides a broader and more comprehensive analysis by integrating reports from domestic birds, wild birds, and mammals together with phylogenetic analyses to characterize the continental spread of HPAI H5N1 across South America and the Antarctic region through May 2025.

## 2. Materials and Methods

### 2.1. Data Gathering and Visualization

We gathered all the reports from the World Animal Health Information System (WAHIS) and from the World Organization for Animal Health (WOAH) from the “Influenza A viruses of high pathogenicity (Inf. with) (non-poultry including wild birds) (2017-)” and “High pathogenicity avian influenza viruses (poultry) (Inf. with)” from South American countries and overseas territories in this continent, as well as in the Antarctic region, up to the 30 May 2025 [23]. We considered all domestic non-poultry as backyard domestic birds, and poultry were classified according to the epidemiological unit reported for each outbreak in the WOAH reports for each country: outbreaks labeled as farms were considered commercial poultry, while those labeled as backyard were considered backyard domestic birds. Because reporting methods varied across countries and overseas territories (for example, the Falkland Islands sometimes reported only deaths, while other countries reported cases and deaths referring to the same individuals), we present cases, deaths, and eliminations as separate columns. As case counts may appear again in the deaths or elimination columns, these values should not be summed. Susceptible wild bird species that were eliminated but were not infected by H5N1 were not included in this study, while susceptible domestic birds (backyard and commercial) that were eliminated as a method of control were included. The conservation status for each affected species, as well as the migratory information for wild mammals, was retrieved from the IUCN Red List of Threatened Species [28], and the migratory information for wild birds was retrieved from Birds of the World from the Cornell Laboratory of Ornithology [29], which resulted in three categories based on bird migratory pattern: trans-equatorial, non-trans-equatorial and non-migratory in South America. We defined trans-equatorial migration as movements in which birds crossed the equator, migrating between South America and Central or North America. The non-trans-equatorial category included all migratory birds that remained within South America, undertaking austral, longitudinal, or altitudinal migrations, as described in Jahn et al. (2020) [30]. Species known to undertake circumpolar movements around Antarctica were retained within this category but were identified separately (Supplementary Table S2) because of their potential epidemiological relevance for viral dissemination within Antarctic and sub-Antarctic ecosystems. We analyzed the retrieved data descriptively, while the timeline graph was created in R (version 4.2.1) with the ‘ggplot2’ package, and the maps with the ‘tmap’, ‘sf’, and ‘rnaturalearth’ packages [31,32,33,34].

### 2.2. Genetic Characterization and Phylogenetic Analysis of Influenza A H5N1 Viruses

All available South American H5N1 sequences were retrieved from the GISAID database (2022–2025). A total of 493 viruses were downloaded, of which 419 possessed complete genome data (all eight segments). The dataset included isolates from South America (Chile, Peru, Argentina, Uruguay, Brazil, Colombia, Venezuela, Bolivia, Ecuador, and the Falkland Islands) and representative sequences from North America, Europe, Asia, and Antarctica. Samples originated from marine mammals, birds, and humans. Quantitative sequence quality control was applied prior to phylogenetic analysis. Sequences were filtered based on: (1) minimum length of 90% of the expected length for each genomic segment; (2) maximum of 5% ambiguous nucleotides (N); and (3) removal of duplicate sequences showing 100% sequence identity, retaining only one representative when multiple identical viral genomes were present. Following alignment trimming and quality filtering, the following number of sequences were retained for each segment: HA (*n* = 379), NA (*n* = 329), NP (*n* = 322), NS (*n* = 265), PA (*n* = 353), PB1 (*n* = 352), PB2 (*n* = 348), and MP (*n* = 260). Detailed information and the accession numbers of the viruses included for each segment are provided in Supplementary Table S6.

For each genomic segment, sequences were aligned using MAFFT v7.526 [35] and manually inspected and edited in BioEdit [36] to remove ambiguous or poorly aligned regions. Duplicate sequences were identified and removed using a custom Python script (https://www.python.org/, Supplementary Method S1) that compared nucleotide sequences character by character after removing alignment gaps. Sequences with identical nucleotide composition were grouped, and a single representative sequence was retained for each group, selected as the first occurrence in the dataset. Following alignment, nucleotide sequences of the HA gene were translated into amino acid sequences to analyze the HA cleavage site across all downloaded viruses, allowing for classification of strains as HPAI or LPAI based on the presence or absence of a polybasic cleavage site. Amino acid sequences were screened for the conserved GLF motif marking the HA1–HA2 junction, and the ten residues upstream of this site were examined. Viruses containing a polybasic cleavage site, defined as at least three arginine or lysine residues including two consecutive basic amino acids, were classified as HPAI, whereas the remaining viruses were classified as LPAI.

To reduce redundancy and avoid overrepresentation of specific outbreaks, identical or nearly identical sequences were removed. In cases where multiple highly similar sequences from the same country or host were present, only one representative was retained, preferentially the earliest collected sample. Maximum likelihood phylogenetic trees were inferred independently for each of the eight segments using IQ-TREE v2.4.0 [37]. The best-fit substitution model for each alignment was selected using ModelFinder [38], and node support was assessed with 1000 ultrafast bootstrap replicates [39]. Trees were visualized and annotated in iTOL v4 [40], incorporating relevant metadata such as collection country and host species. To contextualize the phylogenetic relationships within known viral diversity, genotypes for each virus were assigned using the Genoflu v1.06 tool [41], allowing for interpretation of lineage structure across segments.

We subsequently performed time-calibrated phylogenetic analyses for the HA and NA segments using BEAST v1.10.4 [42]. Each dataset consisted of time-stamped sequences expressed in decimal years. Temporal signal was assessed with TempEst v1.5 [43], which showed a positive correlation between sampling dates and root-to-tip genetic distances, supporting the suitability of molecular clock inference. Root-to-tip regression analysis yielded R^2^ values of 0.5058 for HA and 0.4194 for NA, indicating moderate but significant temporal structure in the dataset that supports phylodynamic inference.

To determine the most appropriate clock model, we compared strict and uncorrelated lognormal relaxed clock models using marginal likelihood estimation (MLE) [44]. In addition, several coalescent demographic priors were evaluated, including constant size, exponential growth, logistic growth, and expansion growth. Model selection was based on MLE comparisons, and the best-fitting combination of clock and tree prior was used for the final analyses.

MCMC chains were run for 50 million generations, sampling every 5000 steps, with three independent replicates conducted to ensure convergence. MCMC convergence was assessed using Tracer v1.7, with convergence criterion defined as effective sample size (ESS) > 200 for all model parameters and tree likelihood. Log and tree files were combined after discarding 10 percent burn-in using LogCombiner. Maximum clade credibility (MCC) trees were summarized with TreeAnnotator and subsequently visualized in Figtree v1.4.4.

## 3. Results

### 3.1. Outbreaks

**Figure 1:**
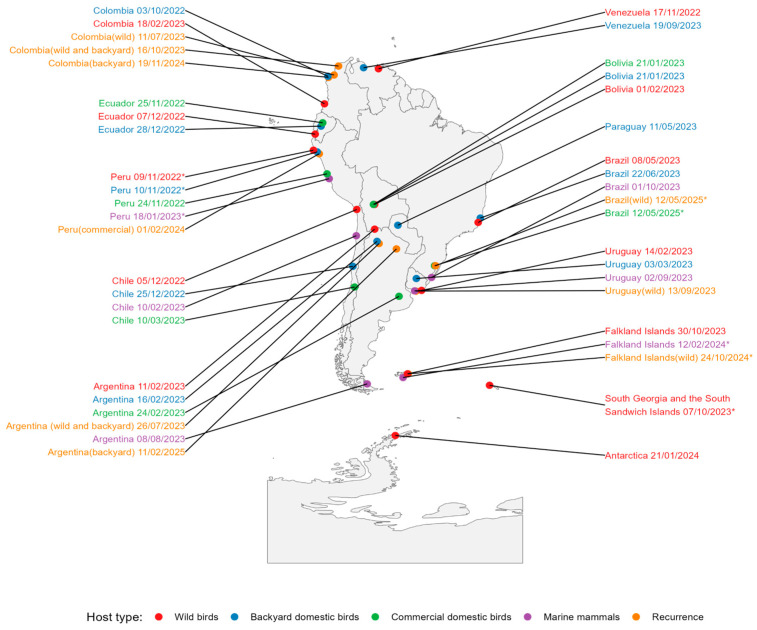
Map of the first highly pathogenic avian influenza (H5N1) outbreaks reported to the World Organization for Animal Health (WOAH) in South American countries, overseas territories in this continent, and the Antarctic region up to the 30 May 2025. This data is documented in the World Animal Health Information System (WAHIS). The map highlights the first reported outbreaks for each host type, wild birds, commercial and backyard domestic birds, and marine mammals, by country or territory, including the Antarctic region. Outbreaks are displayed in each country in chronological order, from the earliest to the most recent (dd/mm/yyyy). * These events were still open at the end of May 2025.

Eleven out of the 14 countries and overseas territories in South America reported HPAI H5N1 to the WOAH either in wild birds, commercial domestic birds, backyard domestic birds, or wild mammals up to May 2025. No cases were reported in French Guiana, Guyana, and Suriname. Among the 11 countries and overseas territories that reported HPAI H5N1 cases, seven (63.64%) first detected the virus in wild birds (Venezuela, Chile, Argentina, Uruguay, Brazil, Peru, and the Falkland Islands). In contrast, Colombia and Paraguay initially reported cases in backyard domestic birds, whereas Ecuador first reported cases in commercial poultry. Bolivia reported concurrent initial outbreaks in both backyard and commercial domestic birds. In addition, in six of the seven countries and overseas territories where wild birds were the initially affected hosts, backyard poultry represented the second group in which the virus was detected; the Falkland Islands were the only exception. A detailed graphical map showing the temporal dynamics of outbreaks across South America is presented in Figure 1.

All South American countries and overseas territories that reported HPAI H5N1 to the WOAH documented cases in wild birds, except for Paraguay. The first wild bird species reported varied by country: brown pelicans (*Pelecanus occidentalis*) in Colombia and Venezuela; Peruvian pelicans (*Pelecanus thagus*) in Peru and Chile; blue-footed boobies (*Sula nebouxii*) in Peru and Ecuador; blue-and-white swallows (*Pygochelidon cyanoleuca*) in Bolivia; Andean geese (*Oressochen melanopterus*) in Argentina; black-necked swans (*Cygnus melancoryphus*) in Uruguay; Cabot’s terns (*Thalasseus sandvicensis* spp. *acuflavidus*) in Brazil; and southern fulmars (*Fulmarus glacialoides*) in the Falkland Islands.

A total of ten South American countries reported HPAI H5N1 cases in backyard domestic birds (Colombia, Peru, Chile, Bolivia, Ecuador, Argentina, Uruguay, Paraguay, Brazil, and Venezuela). In addition, five countries reported cases in marine mammals (Peru, Chile, Argentina, Uruguay, Brazil), as did the Falkland Islands. Cases in commercial poultry were documented in six countries (Argentina, Bolivia, Brazil, Chile, Ecuador, and Peru). Paraguay exclusively reported cases in backyard domestic birds, while the Falkland Islands reported cases only in wild animals (birds and marine mammals). Notably, all marine mammal cases occurred after wild-bird cases.

Several countries and overseas territories have exhibited recurrence of HPAI H5N1 in specific host groups. Wild birds were most frequently reported in recurrent outbreaks, as observed in Argentina, Brazil, Colombia, Uruguay, and the Falkland Islands. Recurrence in backyard poultry was reported in Colombia and Argentina, while Peru was the only country to report recurrence in commercial poultry. The last outbreaks reported in South America, which were ongoing up to May 2025, were reported in Peru, affecting both wild birds and backyard poultry, and in Brazil, where cases persisted in wild birds. Other outbreaks that remain officially open have not reported additional cases.

### 3.2. Affected Species of HPAI in South America

#### 3.2.1. Domestic Birds

**Figure 2:**
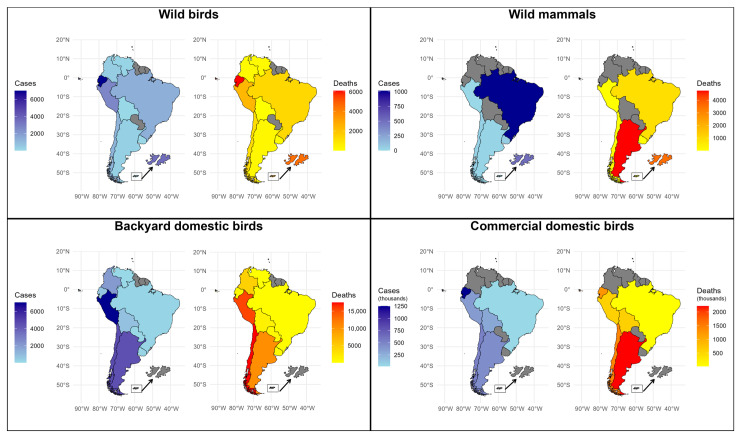
Number of positive cases, as well as deaths (including eliminations) from H5N1 reported by South American countries and overseas territories in this continent to the World Organization of Animal Health (WOAH), until the 30 May 2025. This data is present in the World Animal Health Information System (WAHIS). Given that reporting methods differed across countries and overseas territories, we show positive cases separately from deaths and eliminations. For some countries, positive case counts may appear again in the deaths or elimination data, these values should not be summed.

From 2022 to May 2025, South American countries reported a total of 2,930,410 H5N1-positive domestic birds to the WOAH, with 6,147,176 deaths/eliminations recorded (Supplementary Table S1). In backyard poultry, 20,121 birds tested positive for the virus, while 51,730 died or were eliminated due to this virus. Peru reported the highest number of cases, followed by Chile, Argentina, and Colombia (Figure 2). In contrast, Chile recorded the highest number of deaths and eliminations, followed by Peru.

Regarding commercial domestic birds, more than 2.9 million birds were reported as H5N1-positive across South America, with over 6 million deaths or eliminations. Ecuador registered the highest number of positive cases, followed by Chile and Argentina (Figure 2), while Argentina reported the largest number of deaths and eliminations, followed by Chile and Ecuador (Supplementary Table S1).

#### 3.2.2. Wild Birds

A total of 13,118 confirmed H5N1-positive cases and 14,505 associated deaths or eliminations were recorded in wild birds across South America. In total, 104 wild bird species and five unidentified species were reported. Chile reported the greatest number of species affected (*n* = 57), followed by Brazil (*n* = 34) and Peru (*n* = 27). All other countries reported fewer than 10 species.

In terms of total cases and deaths, Ecuador reported the highest numbers, with 7017 confirmed cases and 6111 fatalities, followed by Peru, Brazil, and Chile (Figure 2). Most of Ecuador’s cases were associated with the magnificent frigatebird (*Fregata magnificens*), which accounted for over 6000 cases and deaths. The most reported species per country can be found in Table 1.

**Figure 3:**
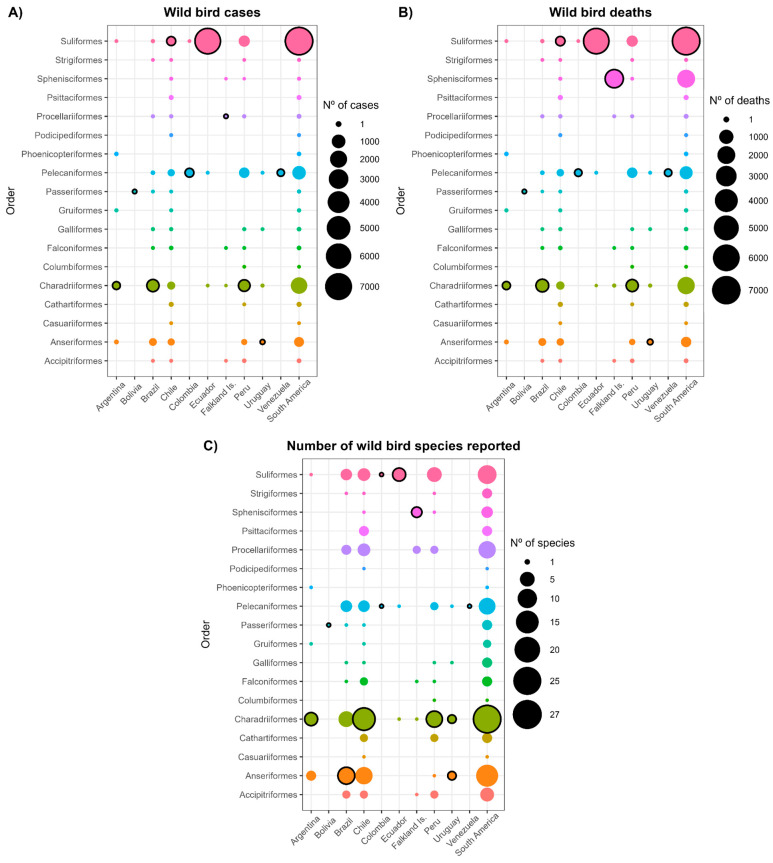
Wild bird orders affected by H5N1 in South American countries, an overseas territory, and South America as a whole, as reported to the World Organization for Animal Health (WOAH) up to 30 May 2025. This data was retrieved from the World Animal Health Information System (WAHIS). Panel (**A**) presents the number of confirmed cases per order; Panel (**B**) shows the number of deaths (including eliminations); and Panel (**C**) shows the number of reported species per order across the same regions. Black circles indicate the most reported order in each country, the overseas territory, or South America overall. Circle colors correspond to the different taxonomic orders, facilitating comparison across countries and panels.

Among the wild bird species reported with HPAI in South America, 27 (24.77%) belonged to the order Charadriiformes, followed by Anseriformes (*n* = 17, 15.6%), Suliformes (*n* = 12, 11%), and Procellariiformes (*n* = 10, 9.17%) (Figure 3C). The number of species reported per order in each country is presented in Figure 3C. Notably, during the recurrence event reported in Brazil beginning in May 2025, 10 of the 12 affected species belonged to the order Anseriformes. However, when considering the impact in terms of the number of cases and mortality, Suliformes (cormorants and boobies) were the most reported order in South America, mainly due to the magnificent frigatebird. The second most reported order was Sphenisciformes (penguins), largely driven by the high mortality observed in Gentoo penguins (*Pygoscelis papua*) in the Falkland Islands. Charadriiformes and Pelecaniformes ranked third and fourth, respectively, in terms of overall impact (Figure 3A,B). The distribution of positive cases among wild-bird orders varied across countries and overseas territories, which can be seen in Figure 3.

Table 1 summarizes the diversity and distribution of HPAI H5N1 detections in wild birds across South America, including their IUCN conservation status. Notably, 33.65% (*n* = 35) of species were reported in more than one country; among these, the South American tern (*Sterna hirundinacea*) and the black-necked swan (*Cygnus melancoryphus*) were each recorded in four countries. Most affected species are classified as Least Concern, although eight are listed as Near Threatened and six as Vulnerable—two of the latter reported in captivity (the tawny eagle and the Andean condor in Peru). Only one species, the waved albatross, is classified as Critically Endangered and was recorded in the wild in Chile. Additionally, a large proportion of affected species were migratory (59.62%, 62/104), of which 67.74% (*n* = 42) were non-trans-equatorial migrants (Supplementary Table S2). Overall, these results highlight the broad taxonomic and geographic impact of HPAI H5N1 on wild birds across the region.

#### 3.2.3. Wild Mammals

No cases of HPAI H5N1 have been reported in domestic mammals in South America up to May 2025, although infections in wild mammals have been documented, totaling 1141 confirmed cases and 5772 deaths or culls. Table 2 summarizes these records, including their distribution and conservation status.

Most affected species are marine mammals, with the South American sea lion (*Otaria flavescens*) being the most frequently reported across five countries. Only one terrestrial species, Geoffroy’s cat (*Leopardus geoffroyi*), was recorded in the wild (in Chile), and the southern elephant seal is the only species classified as migratory by the IUCN. The most threatened species reported are the southern river otter (*Lontra provocax*) and the marine otter (*Lontra felina*), both Endangered and recorded in Chile. All infected wild mammals belong to the order Carnivora.

#### 3.2.4. Presence of HPAI in Antarctica

The first report of HPAI H5N1 to the WOAH was on the 7 October 2023, in the South Georgia and the South Sandwich Islands (SGSSI), where four brown Skua (*Stercorarius antarcticus*) tested positive for H5N1 in Bird Island. Later, H5N1 was found in the South Polar Skua (*Stercorarius maccormicki)* in the Argentinean Antarctic base “Primavera” in January 2024.

The wild bird species reported in the Antarctic region, up to May 2025, can be found in Table 3; all the species are of the order Charadriiformes. The most reported bird was the brown skua, with 14 cases and deaths in the SGSSI. The second most reported bird was the kelp gull (*Larus dominicanus*), which was also reported in Argentina, Chile, and Peru (Table 1). The brown skua and the south polar skua are considered migrants, while the kelp gull is not migratory.

#### 3.2.5. Phylogenetic Analysis of Available Sequences

A total of 501 HA sequences of Influenza A virus were analyzed in this study. Based on the amino acid composition of the HA cleavage site, the majority of sequences were classified as HPAI viruses (Supplementary Table S3). In addition, for seven viruses, the conserved GLF motif required for accurate identification of the cleavage site could not be detected.

Maximum likelihood analysis of all eight viral segments demonstrated consistent phylogenetic topologies across the H5N1 genome. Across all eight genomic segments, the South American B3.2 lineage formed a robust monophyletic clade with consistent topology from polymerase genes (PB2, PB1, PA) through surface and regulatory proteins (HA, NA, NP, MP, NS), with strong bootstrap support (≥70%) across all major nodes. The South American B3.2 lineage exhibited geographic substructure within the monophyletic clade, with sequences from Peru, Chile, Brazil, Argentina, and Uruguay forming distinct sub-clusters in phylogenetic space, indicating country-level genetic differentiation. In contrast, the early-diverging genotypes B1.3 (Colombia) and B2.2 (Venezuela) consisted of limited sequences that did not diversify geographically beyond their countries of origin (Supplementary Figure S1).

**Figure 4:**
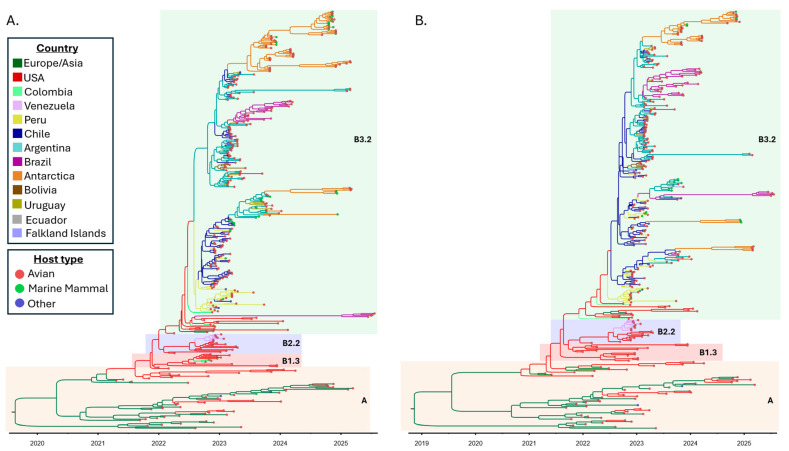
Time-scaled phylogenies of the HA (**A**) and NA (**B**) segments of HPAI H5N1 clade 2.3.4.4b circulating in South America and reference global sequences. Trees were inferred using a Bayesian relaxed molecular clock and are displayed on a calendar timescale (x-axis, years). Branches are colored according to the country of detection. Tip symbols represent host type, where red circles indicate avian hosts, green circles correspond to marine mammals, and blue circles denote other mammals, humans or environmental samples.

The time-scaled phylogeny provides temporal context for the early circulation of HPAI H5N1 in South America during 2022, allowing for estimation of the timing of lineage emergence and subsequent local diversification. Three independent HPAI H5N1 lineages were introduced into South America from North American sources. Early divergence events in Colombia and Venezuela were from genotypes B1.3 (TMRCA: HA 2022.52/NA 2021.92) and B2.2 (TMRCA: HA 2022.60/NA 2022.57), which did not establish sustained transmission. In contrast, genotype B3.2 emerged in mid-2022 in Peru (TMRCA: HA 2022.47/NA 2022.45) and Chile (TMRCA: HA 2022.65/NA 2022.52) and became the only lineage to achieve regional circulation, with first field detections in October–December 2022 from wild bird hosts (owl and pelican) (Figure 4, Supplementary Tables S4 and S5). The contrasting outcomes of B1.3, B2.2, and B3.2 should be interpreted cautiously, because available genomic data alone cannot determine why some introductions failed to establish sustained local circulation while B3.2 became dominant.

Following its initial detection in Peru and Chile, the South American B3.2 lineage exhibited rapid geographic radiation across the continent during late 2022–2023. Early diversification events (late 2022) were detected in Ecuador, Bolivia, Argentina, and Uruguay, followed by spread to Brazil (early 2023) and subsequently to the Antarctic region and the Falkland Islands (mid-2023). The detection of the lineage in both avian (Southern Fulmar) and marine mammal (Southern Elephant Seal) hosts during this expansion highlights the broad host range associated with the regional spread (Figure 4; Supplementary Tables S4 and S5).

## 4. Discussion

This study provides a regional overview of the emergence, spread, and impact of HPAI H5N1 clade 2.3.4.4b in South America and the Antarctic region between 2022 and May 2025. Taken together, the epidemiological and phylogenetic findings show that the current epizootic has affected a wide range of domestic and wild hosts, with relevant implications for poultry production, wildlife conservation, and One Health surveillance in the region.

### 4.1. HPAI in Domestic Birds and the Need to Strengthen Backyard Poultry Surveillance

Although the largest absolute losses were recorded in commercial poultry, the regional pattern in this study suggests that backyard systems represent a critical interface for viral introduction into domestic birds. In most countries where wild birds were affected first, backyard flocks were among the earliest domestic hosts subsequently reported, supporting the hypothesis that these systems frequently act as a bridge between wildlife and poultry production. This is due to the lower biosecurity typically associated with small-scale poultry keeping, which is primarily for self-consumption, and the greater likelihood of contact with wild birds or contaminated environments [45,46]. These findings highlight the need to improve surveillance in backyard poultry, particularly in countries where poultry is raised extensively in rural and peri-urban areas such as Ecuador, Bolivia, and Paraguay [47]. Improving passive and active surveillance in these settings, together with risk communication and practical biosecurity measures, may be essential for earlier detection and control of future incursions.

At the same time, the concentration of reported cases and losses in commercial poultry likely reflects not only the scale of production, but also stronger surveillance and reporting intensity in economically significant sectors. This may explain why some of the largest poultry-producing countries in the region did not necessarily report the highest outbreaks in all domestic host categories, while smaller producers experienced proportionally severe impacts. Beyond direct animal health and economic losses, outbreaks in domestic poultry may also have implications for food availability and supply stability at national and regional levels, particularly where poultry production contributes substantially to affordable animal protein. In this context, the apparent distribution of HPAI H5N1 in domestic birds should be interpreted cautiously, as it may be shaped by differences in detection capacity and reporting practices as much as by true epidemiological risk.

Moreover, Colombia, Ecuador, and Bolivia reported domestic birds as the source of their first outbreaks, consistent with our temporal phylogenetic analysis. In Colombia, available 2022 sequences (*n* = 6) correspond exclusively to domestic species, aligning with WOAH reports. In Ecuador, although phylogenetic results suggest an initial detection in a wild bird (*Fregata magnificens*), this likely reflects a methodological limitation, as only complete genomes were included, and available chicken-derived sequences did not meet this criterion. Similarly, in Bolivia, genomic evidence agrees with WOAH reports, but the limited number of complete genomes (7/16) highlights gaps in surveillance.

### 4.2. Widespread Mortality and Conservation Threats Among South America’s Wild Birds

Wild birds were among the most visibly and extensively affected hosts during the South American HPAI H5N1 epizootic. The concentration of affected species in seabirds, waterbirds, and coastal-associated taxa is consistent with ecological conditions that may favor transmission, including colonial breeding, high host density, carcass exposure, and repeated contact at shared roosting, nesting, and foraging sites [48,49]. This pattern was particularly evident in Ecuador, where mortality was heavily concentrated in magnificent frigatebirds. Despite this extensive mortality, only a small number of H5N1 sequences from the country are publicly available (*n* = 8), and most lack the quality needed for robust phylogenetic reconstruction. This data gap limits a comprehensive assessment of viral spread into and out of Ecuador and hinders our ability to quantify its contribution to regional dissemination patterns, underscoring the need to strengthen genomic surveillance in this setting. More broadly, these findings suggest that pathogen detection in wild animals should be strengthened, particularly in Charadriiformes, Suliformes, Anseriformes, Procellariiformes, and Sphenisciformes, which were among the most frequently affected avian groups in South America.

Although most affected species are currently classified as Least Concern, the conservation implications of HPAI H5N1 should not be underestimated. For long-lived seabirds, scavengers, and colonial breeders, even moderate mortality may have disproportionate demographic effects, particularly where reproductive rates are low, or breeding populations are spatially concentrated [49,50,51]. This concern is especially relevant for the waved albatross (*Phoebastria irrorata*), the only Critically Endangered bird species reported in this study, whose highly restricted breeding distribution may increase vulnerability to localized outbreak events. Because its breeding range is limited to Ecuador [52], where the highest wild bird mortality was reported, HPAI prevention should be considered a conservation priority for this species, requiring coordinated action across Ecuador, Peru, and Chile. A different but also notable concern applies to the Andean condor (*Vultur gryphus*), a Vulnerable species reported in captivity in Peru. If wild populations become infected, associated mortality could threaten long-term population persistence. A similar situation occurred with the critically endangered California condor (*Gymnogyps californianus*) in the United States, where vaccination was implemented as an emergency conservation measure [53], suggesting that comparable strategies may eventually need to be considered in South America.

The high proportion of migratory species among affected wild birds reinforces the broader geographic significance of the outbreak. Migratory connectivity likely contributed both to viral introduction into South America and to its subsequent spread across coastal, pelagic, and subpolar ecosystems, although species likely differed in their epidemiological role depending on their movement ecology and degree of overlap with susceptible resident hosts [20,54,55]. In our dataset, 67.74% of migratory species were non-trans-equatorial migrants, with most of them performing austral migrations, although some species are known to undertake multiple migration strategies [29,30]. This complexity is well illustrated by the southern giant-petrel (*Macronectes giganteus*), which has been recorded migrating north from Antarctica to Brazil [56] and whose banding records also indicate movement across South America, South Africa, Australia, New Zealand, Madagascar, and the Indian Ocean [57,58]. Such broad dispersal highlights its potential to facilitate viral movement across subpolar and intercontinental systems. Although only two individuals were reported in Chile in our dataset, the confirmation of HPAI H5N1 in this species in the Antarctic region suggests that it may contribute to viral dissemination well beyond the locations where outbreaks are first detected. Understanding these differences will be essential for identifying which species and flyway connections are most relevant for future surveillance and early warning efforts.

### 4.3. HPAI Spillover into Mammals and Implications for Surveillance

Spillover into wild mammals has been documented globally [25,26,59,60], but its extent and patterns in South America remain poorly characterized. Our phylogenetic analysis reveals that spillovers in the region were indeed a repeated and geographically widespread feature rather than an isolated event. Mammalian viruses consistently clustered within avian lineages rather than forming a distinct mammalian clade, indicating repeated avian-to-mammal transmission events. This pattern is consistent with the ecology of coastal pinnipeds exposed to infected wild birds or contaminated carcasses and demonstrates that spillover events are a central feature of HPAI dynamics in South America.

The zoonotic dimension of these spillover events also warrants attention. Although only two human cases have been reported in South America [61], current genomic data remains insufficient to robustly reconstruct transmission pathways. In the Ecuadorian case, the publicly available human sequence includes only the HA and NA segments, highlighting the need for complete genome sequencing to allow for reliable phylogenetic inference, reassortment detection, and accurate assessment of zoonotic transmission pathways. In contrast, the Chilean human virus was included in our phylogenetic analyses and clustered with avian and marine mammal sequences, suggesting a wildlife-associated origin. However, future analyses, such as phylogeographic reconstruction or Markov jump models, would be required to formally assess transmission pathways and test hypotheses regarding cross-species spread.

Repeated spillover into mammals may create opportunities for the selection of host-adaptive mutations. In South America, PB2 substitutions associated with mammalian adaptation, including D701N and Q591K, have been reported in the Chilean human H5N1 case and in Chilean marine mammal viruses [26,62]. However, their detection should be interpreted as evidence of mammalian replication and potential adaptation, not as proof of sustained zoonotic transmission.

Taken together, these findings indicate that mammalian infections should be considered a central component of HPAI surveillance in South America rather than a secondary consequence of avian outbreaks. Expanding systematic surveillance to include marine mammals, terrestrial carnivores, captive wildlife, and domestic mammals, together with more complete genomic sequencing, will be essential for detecting viral adaptation early and for better understanding the zoonotic and ecological risks posed by continued H5N1 circulation in the region.

### 4.4. Evidence of H5N1 Transmission Between Avifauna in South America and Antarctica

The emergence of HPAI H5N1 in Antarctica in October 2023 represents a critical expansion of the virus into one of the most ecologically sensitive regions on Earth. Before this event, only LPAI subtypes such as H6N8, H11N2, and H5N5 had been detected on the continent [63,64,65]. Antarctic and sub-Antarctic ecosystems are characterized by dense breeding colonies, strong site fidelity, and species with low reproductive rates, making them particularly vulnerable to acute mortality events and long-term population impacts [66,67,68].

Although the precise pathways of introduction cannot be definitively resolved, the available epidemiological and ecological evidence is consistent with seabird-mediated southward dissemination from South America into sub-Antarctic and Antarctic regions [69,70]. Initial detections in South Georgia and the South Sandwich Islands, followed by cases in continental Antarctica, support a stepwise spread through ecologically connected regions [29,71]. This hypothesis is further supported by the occurrence of H5N1 in several species shared between South America and Antarctica, including the southern giant petrel, gentoo penguin, and black-browed albatross, which may facilitate viral movement across geographic boundaries through overlapping wintering grounds [29]. In addition, several seabird species undertake circumpolar movements around Antarctica, potentially contributing to viral dissemination among Antarctic and sub-Antarctic colonies after the virus has been introduced into the region. Although direct evidence for this mechanism remains limited, these movement patterns may enhance ecological connectivity among breeding colonies and facilitate the regional spread of HPAI H5N1 within the Southern Ocean [72]. Together, these findings highlight the importance of ecological connectivity in the spread of HPAI H5N1 and underscore the need for enhanced surveillance of migratory seabirds and other polar wildlife to better understand and mitigate the risks posed by the virus in Antarctica.

Our temporal phylogenetic analysis provides further support for this connectivity. We identified two distinct Antarctic viral lineages, one of Argentine origin and another of Chilean origin, both associated with viruses of avian origin detected in animal hosts. These findings suggest that Antarctic incursions are not isolated events but reflect the continuation of southward viral spread through interconnected avian populations.

### 4.5. Limitations and Future Directions

This study provides a comprehensive analysis of the introduction and spread of HPAI H5N1 in South America and the Antarctic region. However, the exclusive use of WOAH reports represents an important limitation, as these records likely underestimate the true burden of infection and mortality in wild animal populations. Evidence from multiple regions suggests that confirmed H5N1 cases represent only a fraction of the outbreak’s actual impact. For example, extensive mortality events among pinnipeds along the Pacific coast indicate that laboratory-confirmed infections account for only a small proportion of affected animals [73]. In Chile, sea lion strandings increased dramatically during the first half of 2023, surpassing 22,000 individuals by 2024, with HPAI considered the most likely cause of mortality [74,75]. Despite this large-scale mortality event, the WOAH reported only 39 confirmed HPAI cases in marine and river otters and sea lions. Likewise, comparisons between international wild bird notifications and national mortality records or field observations have revealed substantial underreporting [76,77], particularly in coastal and remote areas where carcass detection, sampling, and laboratory confirmation remain challenging.

A similar pattern was observed in the Antarctic region. WOAH reports available through May 2025 underestimated the extent of HPAI circulation when compared with records published by the Scientific Committee on Antarctic Research (SCAR) [67]. SCAR documented confirmed infections in additional species, including gentoo penguins, wandering albatrosses (*Diomedea exulans*), southern giant petrels, and marine mammals that were not represented in WOAH notifications. Collectively, these discrepancies indicate that official WOAH reports should be regarded as a minimum estimate of HPAI occurrence and mortality, rather than a comprehensive assessment of the outbreak’s ecological impact on wildlife populations.

Furthermore, this study provides temporal phylogenetic insights that also have important limitations. The unequal geographic distribution of sequences, with Ecuador underrepresented despite high bird mortality, constrains transmission pattern interpretation. Phylogeographic reconstruction and formal reassortment detection (RDP4, GARD) were beyond the scope of this temporal analysis. However, temporal phylogenetic methods are less sensitive to geographic bias than phylogeographic approaches and provide robust evolutionary rate estimates.

Future priorities include: (1) strengthened wildlife surveillance systems; (2) enhanced regional genomic surveillance infrastructure; (3) complete human H5N1 genome sequencing; (4) segment-level analysis for reassortment characterization; and (5) phylogeographic reconstruction with balanced geographic sampling.

Our findings suggest that HPAI surveillance in South America should be organized around the major epidemiological interfaces highlighted by this study rather than relying on uniform surveillance approaches. At the wildlife–backyard poultry interface, surveillance should prioritize areas where small-scale poultry production overlaps with habitats used by wild birds, complemented by practical biosecurity measures, community engagement, and timely reporting of suspected cases. Along coastal wetlands, breeding colonies, and migratory flyways, surveillance should focus on species and locations with the greatest potential to facilitate long-distance viral dissemination, particularly species that connect distinct ecological regions within South America or link South America with sub-Antarctic and Antarctic ecosystems. Similarly, systematic monitoring of marine mammal colonies and stranding events should be integrated with avian surveillance, given the repeated spillover events documented in this study. However, it is important to emphasize that improving surveillance will also require a better understanding of the movement ecology of wild birds. Significant knowledge gaps remain regarding the migration routes, seasonal movements, and ecological connectivity of many South American and Southern Ocean species, particularly those migrating within the Southern Hemisphere. Addressing these gaps will be essential to identifying the species most likely to disseminate HPAI H5N1, refining risk assessments, and optimizing surveillance efforts across the region. Together, these actions would provide a more targeted and operational One Health framework for early detection and response to future HPAI incursions.

## 5. Conclusions

Taken together, these findings indicate that the emergence of HPAI H5N1 clade 2.3.4.4b in South America represents a large-scale, multi-host epizootic shaped by ecological connectivity, repeated cross-species transmission, and uneven surveillance capacity, rather than a localized outbreak in domestic poultry. The expansion of the virus into marine mammals and Antarctic ecosystems highlights the ecological connectivity underlying viral dissemination at a continental scale.

The exclusive use of WOAH reports is a substantial limitation of this study, as it likely understates the true mortality among wild animals. Consequently, mortality records may be subject to reporting biases, while phylogeographic inferences may be affected by uneven genomic sampling across the continent. These constraints call for cautious interpretation of our findings, as they may not fully capture the epidemiological scenario. This highlights the need to strengthen collaborative surveillance networks among South American countries, harmonize case reporting practices, expand sequencing efforts, and promote open genomic data sharing—all of which are essential to better characterize and understand viral dissemination dynamics across the region.

These findings provide a novel resolution beyond previous reports and highlight the extensive inter-country connectivity of circulating viruses. The unprecedented detection of HPAI in Antarctica further illustrates the ecological risks posed by the ongoing southward spread. Looking forward, the combined effects of climate change and limited surveillance, particularly in backyard and marine systems, pose ongoing challenges to outbreak detection and control. These findings underscore the urgent need for a coordinated One Health approach in South America, integrating wildlife, domestic animal, and human health systems while prioritizing surveillance at these key epidemiological interfaces. Finally, a better understanding of the movement ecology of wild birds, particularly species migrating within the Southern Hemisphere, will be essential to predict future pathways of HPAI H5N1 spread and to develop more effective risk-based surveillance strategies across South America and the Antarctic region.

## Figures and Tables

**Table 1:** Wild bird species that were reported by South American countries and overseas territories in this continent to the World Organization for Animal Health (WOAH) for “Influenza A viruses of high pathogenicity” until the 30 May 2025. This data is present in the World Animal Health Information System (WAHIS). The global assessment of the IUCN Red List Category was included for each species. Given that reporting methods differed across countries and overseas territories, we show columns for positive cases, deaths and eliminations. For some countries, positive case counts may appear again in the deaths or elimination columns, these values should not be summed. * This symbol in the country column indicates the species with the highest reported number of cases or deaths/eliminations in each country or overseas territory.

Species	Scientific Name	Country or Overseas Territory	Cases	Deaths	Eliminations	IUCN
American golden plover	*Pluvialis dominica*	Brazil	1	0	1	Least concern
American oystercatcher	*Haematopus palliatus*	Chile	1	1	0	Least concern
Anatidae (unidentified)		Argentina	3	3	0	
Andean condor	*Vultur gryphus*	Peru	1	1	0	Vulnerable
Andean goose	*Oressochen melanopterus*	Argentina	2	2	0	Least concern
		Peru	102	102	0	
Anhinga	*Anhinga anhinga*	Brazil	2	2	0	Least concern
Antarctic Prion	*Pachyptila desolata*	Brazil	1	0	1	Least concern
Austral parakeet	*Enicognathus ferrugineus*	Chile	1	0	1	Least concern
Belcher’s gull	*Larus belcheri*	Chile	27	27	0	Least concern
		Peru	0	1	0	
Black skimmer	*Rynchops niger*	Chile	6	6	0	Least concern
		Peru	12	11	0	
Black swan	*Cygnus atratus*	Brazil	96	84	0	Least concern
Black vulture	*Coragyps atratus*	Chile	5	5	0	Least concern
		Peru	1	1	0	
Black-browed albatross	*Thalassarche melanophris*	Chile	1	1	0	Least concern
		Falkland Is.	1	1	0	
Black-chested buzzard-eagle	*Geranoaetus melanoleucus*	Chile	1	1	0	Least concern
		Peru	13	10	2	
Black-crowned Night-Heron	*Nycticorax nycticorax*	Chile	2	2	0	Least concern
Black-faced ibis	*Theristicus melanopis*	Chile	1	1	0	Least concern
Blackish oystercatcher	*Haematopus ater*	Chile	4	4	0	Least concern
Black-necked swan	*Cygnus melancoryphus*	Argentina	21	19	0	Least concern
		Brazil	141	132	9	
		Chile	123	119	4	
		Uruguay *	14	14	0	
Blue-and-white swallow	*Pygochelidon cyanoleuca*	Bolivia	4	4	0	Least concern
Blue-footed booby	*Sula nebouxii*	Ecuador	4	3	1	Least concern
		Peru	4	2	1	
Brown Booby	*Sula leucogaster*	Brazil	3	0	3	Least concern
Brown pelican	*Pelecanus occidentalis*	Colombia *	376	167	1	Least concern
		Venezuela	172	172	0	
Brown skua	*Stercorarius antarcticus*	Falkland Is.	0	7	0	Least concern
Brown-hooded gull	*Chroicocephalus maculipennis*	Brazil	1	0	1	Least concern
		Chile	7	7	0	
		Peru	10	8	0	
Burrowing parrot	*Cyanoliseus patagonus*	Chile	2	0	2	Least concern
Cabot’s tern	*Thalasseus sandvicensis* spp. *acuflavidus*	Brazil *	915	828	87	Least concern
Cayenne tern	*Thalasseus sandvicensis* spp. *eurygnathus*	Argentina	1	1	0	Least concern
Chilean flamingo	*Phoenicopterus chilensis*	Argentina	10	10	0	Near threatened
Chilean skua	*Stercorarius chilensis*	Chile	17	17	0	Least concern
Chimango caracara	*Daptrius chimango*	Chile	9	9	0	Least concern
Cinnamon teal	*Spatula cyanoptera*	Chile	5	0	5	Least concern
Cocoi heron	*Ardea cocoi*	Brazil	1	1	0	Least concern
Common quail	*Coturnix coturnix*	Chile	5	5	0	Least concern
Common tern	*Sterna hirundo*	Brazil	27	19	8	Least concern
Coscoroba swan	*Coscoroba coscoroba*	Brazil	9	9	0	Least concern
		Chile	15	12	3	
Crested caracara	*Caracara plancus*	Brazil	2	1	1	Least concern
		Falkland Is.	1	0	1	
Dolphin gull	*Leucophaeus scoresbii*	Chile	2	2	0	Least concern
Elegant tern	*Thalasseus elegans*	Chile	11	11	0	Near threatened
Emu	*Dromaius novaehollandiae*	Chile	1	1	0	Least concern
Flightless steamer duck	*Tachyeres pteneres*	Chile	4	4	0	Least concern
Franklin’s gull	*Leucophaeus pipixcan*	Chile	5	5	0	Least concern
Fregatidae (unidentified)		Ecuador	3	1	0	
		Peru	12	10	0	
Gentoo penguin	*Pygoscelis papua*	Falkland Is. *	0	2300	0	Least concern
Gray gull	*Leucophaeus modestus*	Chile	43	43	0	Least concern
		Peru *	837	831	1	
Graylag goose	*Anser anser*	Brazil	4	0	0	Least concern
		Chile	2	2	0	
		Uruguay	2	2	28	
Great black-hawk	*Buteogallus urubitinga*	Brazil	1	0	1	Least concern
Great egret	*Ardea alba*	Brazil	1	0	1	Least concern
		Peru	1	1	0	
		Uruguay	1	1	0	
Great frigatebird	*Fregata minor*	Colombia	1	1	0	Least concern
		Ecuador	1002	101	0	
Great grebe	*Podiceps major*	Chile	4	4	0	Least concern
Grey-hooded gull	*Chroicocephalus cirrocephalus*	Brazil	2	0	2	Least concern
		Peru	2	2	0	
Guanay cormorant	*Leucocarbo bougainvilliorum*	Chile	161	161	0	Near threatened
		Peru	197	180	1	
Gull-billed tern	*Gelochelidon nilotica*	Argentina	2	2	0	Least concern
Harris’s hawk	*Parabuteo unicinctus*	Chile	2	2	0	Least concern
Helmeted guineafowl	*Numida meleagris*	Peru	4	1	0	Least concern
House sparrow	*Passer domesticus*	Chile	1	1	0	Least concern
Humboldt penguin	*Spheniscus humboldti*	Chile	5	5	0	Vulnerable
		Peru	0	1	0	
Imperial cormorant	*Leucocarbo atriceps*	Argentina	2	2	0	Least concern
		Chile	3	3	0	
Inca tern	*Larosterna inca*	Chile	24	24	0	Near threatened
Indian peafowl	*Pavo cristatus*	Brazil	4	1	0	Least concern
		Uruguay	1	1	0	
Kelp gull	*Larus dominicanus*	Argentina	22	22	0	Least concern
		Chile	71	68	3	
		Peru	16	15	1	
Laughing gull	*Leucophaeus atricilla*	Ecuador	1	0	0	Least concern
Lesser horned owl	*Bubo magellanicus*	Chile	1	1	0	Least concern
Lesser yellowlegs	*Tringa flavipes*	Chile	1	1	0	Vulnerable
Magnificent frigatebird	*Fregata magnificens*	Brazil	1	0	1	Least concern
		Ecuador *	6000	6000	0	
		Peru	210	104	0	
Mandarin duck	*Aix galericulata*	Chile	4	0	4	Least concern
Manx shearwater	*Puffinus puffinus*	Brazil	4	1	3	Least concern
Muscovy duck	*Cairina moschata*	Brazil	8	8	0	Least concern
Mute swan	*Cygnus olor*	Brazil	2	2	0	Least concern
Neotropic cormorant	*Nannopterum brasilianum*	Brazil	2	0	2	Least concern
		Chile	17	17	0	
		Peru	0	1	0	
Peregrine falcon	*Falco peregrinus*	Chile	3	3	0	Least concern
		Peru	2	2	0	
Peruvian booby	*Sula variegata*	Chile *	199	199	0	Least concern
		Peru	457	459	6	
Peruvian diving petrel	*Pelecanoides garnotii*	Chile	2	2	0	Near threatened
Peruvian pelican	*Pelecanus thagus*	Chile	189	154	35	Near threatened
		Peru	707	583	31	
Rallidae (unidentified)		Argentina	4	4	0	
Red-footed booby	*Sula sula*	Ecuador	6	5	0	Least concern
Red-gartered coot	*Fulica armillata*	Chile	3	3	0	Least concern
Red-legged cormorant	*Poikilocarbo gaimardi*	Chile	18	18	0	Near threatened
		Peru	22	21	1	
Ringed teal	*Callonetta leucophrys*	Brazil	2	2	0	Least concern
Roadside hawk	*Rupornis magnirostris*	Brazil	1	0	1	Least concern
Rock pigeon	*Columba livia*	Peru	2	0	2	Least concern
Royal tern	*Thalasseus maximus*	Argentina	3	3	0	Least concern
		Brazil	88	36	52	
		Uruguay	1	1	0	
Rufous hornero	*Furnarius rufus*	Brazil	1	0	0	Least concern
Sanderling	*Calidris alba*	Chile	5	5	0	Least concern
Slender-billed parakeet	*Enicognathus leptorhynchus*	Chile	30	24	6	Least concern
Snowy egret	*Egretta thula*	Brazil	1	0	1	Least concern
		Chile	3	3	0	
Sooty shearwater	*Ardenna grisea*	Chile	3	3	0	Near threatened
		Peru	6	1	0	
South American tern	*Sterna hirundinacea*	Argentina *	208	208	0	Least concern
		Brazil	15	6	9	
		Chile	85	85	0	
		Uruguay	5	4	1	
South polar skua	*Stercorarius maccormicki*	Peru	25	20	5	Least concern
Southern fulmar	*Fulmarus glacialoides*	Falkland Is.	2	2	0	Least concern
Southern giant-petrel	*Macronectes giganteus*	Chile	2	2	0	Least concern
Southern lapwing	*Vanellus chilensis*	Brazil	1	0	1	Least concern
		Chile	2	2	0	
Southern rockhopper penguin	*Eudyptes chrysocome*	Falkland Is.	0	30	0	Vulnerable
Spheniscidae (unidentified)		Falkland Is.	0	400	0	
Strigiformes (unidentified)		Peru	1	1	0	
Tawny eagle	*Aquila rapax*	Peru	3	1	0	Vulnerable
Tropical screech-owl	*Megascops choliba*	Brazil	1	0	1	Least concern
Turkey vulture	*Cathartes aura*	Chile	31	30	1	Least concern
Upland goose	*Chloephaga picta*	Chile	7	6	1	Least concern
Variable hawk	*Geranoaetus polyosoma*	Falkland Is.	1	1	0	Least concern
Waved albatross	*Phoebastria irrorata*	Peru	4	4	0	Critically endangered
Whimbrel	*Numenius phaeopus*	Chile	2	2	0	Least concern
		Peru	1	0	1	
White-chinned petrel	*Procellaria aequinoctialis*	Brazil	2	0	2	Vulnerable
White-faced ibis	*Plegadis chihi*	Brazil	11	10	0	Least concern
White-faced whistling duck	*Dendrocygna viduata*	Brazil	16	0	0	Least concern
Wilson’s storm-petrel	*Oceanites oceanicus*	Chile	1	1	0	Least concern
Wood Duck	*Aix sponsa*	Brazil	1	1	0	Least concern
		Chile	24	0	24	
Yellow-billed pintail	*Anas georgica*	Chile	7	7	0	Least concern
Yellow-billed teal	*Anas flavirostris*	Chile	11	11	0	Least concern
Yellow-crowned night heron	*Nyctanassa violacea*	Ecuador	1	1	0	Least concern
TOTAL			13,118	14,144	361	

**Table 2:** Wild mammal species reported by South American countries and overseas territories in this continent to the World Organization for Animal Health (WOAH) for “Influenza A viruses of high pathogenicity” until the 30 May 2025. This data is present in the World Animal Health Information System (WAHIS). The global assessment of the IUCN Red List Category was included for each species. Given that reporting methods differed across countries and overseas territories, we show columns for positive cases, deaths and eliminations. For some countries, positive case counts may appear again in the deaths or elimination columns, these values should not be summed.

Species	Scientific Name	Country	Nº of Cases	Nº of Deaths	Nº of Eliminated	IUCN Assessment
Geoffroy’s Cat	*Leopardus geoffroyi*	Chile	1	1	0	Least concern
Lion	*Panthera leo*	Peru	1	1	0	Vulnerable
Marine otter	*Lontra felina*	Chile	2	2	0	Endangered
Southern river otter	*Lontra provocax*	Chile	1	1	0	Endangered
South American Coati	*Nasua nasua*	Uruguay	16	16	0	Least concern
South American fur seal	*Arctocephalus australis*	Argentina	0	44	0	Least concern
		Uruguay	8	8	0	
		Brazil	439	381	1	
South American sea lion	*Otaria flavescens*	Peru	2	2	0	Least concern
		Chile	36	36	0	
		Argentina	17	2134	0	
		Uruguay	32	32	0	
		Brazil	578	488	6	
Southern elephant seal	*Mirounga leonina*	Argentina	8	2579	0	Least concern
		Falkland Islands	0	40	0	
TOTAL			1141	5765	7	

**Table 3:** Wild bird species in the Antarctic region that were reported to the World Organization for Animal Health (WOAH) for “Influenza A viruses of high pathogenicity” until the 30 May 2025. This data is present in the World Animal Health Information System (WAHIS). The global assessment of the IUCN Red List Category was included for each species.

Species	Scientific Name	Antarctic Region	Nº of Cases	Nº of Deaths	Nº of Eliminated	IUCN Assessment
Brown skua	*Stercorarius antarcticus*	Lagoon Island	2	2	0	Least concern
		South Georgia and the South Sandwich Islands	14	14	0	
South polar skua	*Stercorarius maccormicki*	Antarctic base “Primavera”	1	1	0	Least concern
Kelp gull	*Larus dominicanus*	South Georgia and the South Sandwich Islands	8	8	0	Least concern
TOTAL			25	25	0	

## Data Availability

Epidemiological data used in this study were obtained from the World Organization for Animal Health (WOAH) and the World Animal Health Information System (WAHIS), and genomic sequence data were obtained from the GISAID EpiFlu database.

## Supplementary Materials

The following supporting information can be downloaded at: https://www.mdpi.com/article/10.3390/v18070764/s1, Supplementary Method S1. Python Script for Duplicate Sequence Removal; Table S1. Summary of cases and deaths of all host types per country; Table S2. Classification of migration types among wild bird species affected by H5N1 in South America, 2022–May 2025; Table S3. Pathogenicity classification of avian influenza A viruses used in this study based on the HA0 cleavage site; Table S4. Inferred ancestral origins of avian influenza A viruses based on the HA segment; Table S5. Inferred ancestral origins of avian influenza A viruses based on the NA segment; Table S6. Complete sequence metadata used for phylogenetic analysis; Figure S1. Maximum likelihood phylogenetic trees of the eight genomic segments of HPAI H5N1 clade 2.3.4.4b circulating in South America.

## Abbreviations

The following abbreviations are used in this manuscript:

HPAI	Highly Pathogenic Avian Influenza
LPAI	Low-Pathogenic Avian influenza
WOAH	World Animal Health Agency
