# Supplementary material for: Highly Pathogenic Avian Influenza H5N1 in South America, 2022–2025: Spread, Affected Species, and Southward Expansion into the Antarctic Region
Source: Viruses. 2026 Jul 12;18(7):764. doi: 10.3390/v18070764 (PMC13431583; doi:10.3390/v18070764)

Supplementary Methods S1. Python Script for Duplicate Sequence Removal Duplicate sequences were identified using a Python 3 script leveraging the BioPython library (SeqIO module). The algorithm: (1) loads all sequences from the aligned FASTA file; (2) removes alignment gaps ("-" characters) from each sequence; (3) converts sequences to uppercase for standardized comparison; (4) performs exact character-by-character comparison; (5) groups identical sequences together; (6) retains the first occurrence as the representative; and (7) outputs a deduplicated FASTA file and a report of duplicate groups. Code (Python 3.7+):

```python
from Bio import SeqIO
from collections import defaultdict


input_fasta = "input.fasta"
output_groups = "input_identicos.txt"
output_representatives = "input_unicos.fasta"


seq_dict = defaultdict(list)

for record in SeqIO.parse(input_fasta, "fasta"):
    seq_str = str(record.seq).upper().replace("-", "")
    seq_dict[seq_str].append(record.id)


with open(output_groups, "w") as out:
    for i, (seq, headers) in enumerate(seq_dict.items(), start=1):
        if len(headers) > 1:
            out.write(f"Grupo {i} ({len(headers)} secuencias):\n")
            for h in headers:
                out.write(f"  {h}\n")
            out.write("\n")

print(f"Archivo con grupos duplicados: {output_groups}")


representatives = []

for seq, headers in seq_dict.items():
    rep = headers[0]
    representatives.append(rep)

with open(output_representatives, "w") as out_fasta:
    for seq, headers in seq_dict.items():
        rep_id = headers[0]
        out_fasta.write(f">{rep_id}\n{seq}\n")

print(f"Archivo FASTA sin duplicados: {output_representatives}")
```

Supplementary Table S1. Total positive cases, deaths, and eliminations of host species affected in South American countries and overseas territories, 2022–May 2025. Given that reporting methods differed across countries and overseas territories, we show columns for positive cases, deaths and eliminations. For some countries, positive case counts may appear again in the deaths or elimination columns, these values should not be summed.

	Nº of cases	Nº of deaths	Nº of eliminations	Sum of deaths and eliminations
**Backyard**	**20,121**	**16,446**	**35,284**	**51,73**
Argentina	4,514	3,885	6,642	10,527
Bolivia	764	627	688	1,315
Brazil	170	44	325	369
Chile	4,688	2,891	14,611	17,502
Colombia	2,052	1,68	2,899	4,579
Ecuador	642	229	413	642
Paraguay	68	355	396	751
Peru	7,046	6,589	8,859	15,448
Uruguay	162	144	398	542
Venezuela	15	2	53	55
**Commercial**	**2,910,289**	**2,174,132**	**3,921,314**	**6,095,446**
Argentina	450,596	424,166	1,790,366	2,214,532
Bolivia	296,094	233,194	357,439	590,633
Brazil	15,662	15,662	1,363	17,025
Chile	528,255	505,152	930,129	1,435,281
Ecuador	1,251,693	923,456	328,237	1,251,693
Peru	367,989	72,502	513,78	586,282
**Total domestic birds**	**2,930,410**	**2,190,578**	**3,956,598**	**6,147,176**
**Wild mammals**	**1,141**	**5,765**	**7**	**5,772**
Argentina	25	4757	0	4757
Brazil	1017	869	7	876
Chile	40	40	0	40
Falkland Is.	0	40	0	40
Peru	3	3	0	3
Uruguay	56	56	0	56

**Wild bird**	**13,118**	**14,144**	**361**	**14,505**
Argentina	278	276	0	276
Bolivia	4	4	0	4
Brazil	1,368	1,143	188	1,331
Chile	1,221	1,132	89	1,221
Colombia	377	168	1	169
Ecuador	7,017	6,111	1	6,112
Falkland Is.	5	2,741	1	2,742
Peru	2,652	2,374	52	2,426
Uruguay	24	23	29	52
Venezuela	172	172	0	172
**TOTAL**	**2,944,669**	**2,210,487**	**3,956,966**	**6,167,453**

Supplementary Table S2. Classification of migration types among wild bird species affected by H5N1 in South America, 2022–May 2025.

Type of migrator	Common name	Scientific name
Trans-equatorial	American Golden Plover	*Pluvialis dominica*
	Cabot's Tern	*Thalasseus sandvicensis spp. acuflavidus*
	Cayenne tern	*Thalasseus sandvicensis spp. eurygnath*
	Common Tern	*Sterna hirundo*
	Elegant Tern	*Thalasseus elegans*
	Franklin's gull	*Leucophaeus pipixcan*
	Gull-billed tern	*Gelochelidon nilotica*
	Laughing gull	*Leucophaeus atricilla*
	Lesser yellowlegs	*Tringa flavipes*
	Magnificent Frigatebird	*Fregata magnificens*
	Manx Shearwater	*Puffinus puffinus*
	Peregrine falcon	*Falco peregrinus*
	Royal Tern	*Thalasseus maximus*
	Sanderling	*Calidris alba*
	Snowy egret	*Egretta thula*
	Sooty shearwater	*Ardenna grisea*
	South polar skua	*Stercorarius maccormicki*
	Whimbrel	*Numenius phaeopus*
	Wilson's storm-petrel	*Oceanites oceanicus*
	Yellow-crowned night heron	*Nyctanassa violacea*
	Andean condor	*Vultur gryphus*
	Andean goose	*Oressochen melanopterus*
	Antarctic Prion	*Pachyptila desolata*
	Austral parakeet	*Enicognathus ferrugineus*
	Black skimmer	*Rynchops niger*
	Black-browed albatross	*Thalassarche melanophris*
	Black-faced ibis	*Theristicus melanopis*
	Black-necked Swan	*Cygnus melancoryphus*
	Blue-and-white swallow	*Pygochelidon cyanoleuca*
	Brown pelican	*Pelecanus occidentalis*
	Brown skua	*Stercorarius antarcticus*
	Brown-hooded Gull	*Chroicocephalus maculipennis*
	Burrowing parrot	*Cyanoliseus patagonus*
	Chilean Flamingo	*Phoenicopterus chilensis*
	Chilean Skua	*Stercorarius chilensis*
	Chimango caracara	*Daptrius chimango*
	Cinnamon teal	*Spatula cyanoptera*
	Cocoi heron	*Ardea cocoi*
	Coscoroba Swan	*Coscoroba coscoroba*

**Non-trans-equatorial**	Dolphin gull	*Leucophaeus scoresbii*
	Gentoo penguin	*Pygoscelis papua*
	Gray gull	*Leucophaeus modestus*
	Great Grebe	*Podiceps major*
	Grey-hooded gull	*Chroicocephalus cirrocephalus*
	Humboldt penguin	*Spheniscus humboldti*
	Inca Tern	*Larosterna inca*
	Peruvian diving petrel	*Pelecanoides garnotii*
	Peruvian pelican	*Pelecanus thagus*
	Ringed teal	*Callonetta leucophrys*
	Slender-billed Parakeet	*Enicognathus leptorhynchus*
	South American Tern	*Sterna hirundinacea*
	Southern fulmar	*Fulmarus glacialoides*
	Southern Giant-Petrel	*Macronectes giganteus*
	Southern rockhopper penguin	*Eudyptes chrysocome*
	Turkey Vulture	*Cathartes aura*
	Upland Goose	*Chloephaga picta*
	Variable Hawk	*Geranoaetus polyosoma*
	Waved albatross	*Phoebastria irrorata*
	White-chinned Petrel	*Procellaria aequinoctialis*
	White-faced Ibis	*Plegadis chihi*
	Yellow-billed pintail	*Anas georgica*
	Yellow-billed teal	*Anas flavirostris*
**Non-migratory**	American Oystercatcher	*Haematopus palliatus*
	Anhinga	*Anhinga anhinga*
	Belcher's gull	*Larus belcheri*
	Black swan	*Cygnus atratus*
	Black vulture	*Coragyps atratus*
	Black-chested buzzard-eagle	*Geranoaetus melanoleucus*
	Black-crowned Night-Heron	*Nycticorax nycticorax*
	Blackish Oystercatcher	*Haematopus ater*
	Blue-footed booby	*Sula nebouxii*
	Brown Booby	*Sula leucogaster*
	Common Quail	*Coturnix coturnix*
	Crested Caracara	*Caracara plancus*
	Emu	*Dromaius novaehollandiae*
	Flightless steamer duck	*Tachyeres pteneres*
	Graylag Goose	*Anser anser*
	Great Black-Hawk	*Buteogallus urubitinga*
	Great egret	*Ardea alba*
	Great frigatebird	*Fregata minor*
	Guanay cormorant	*Leucocarbo bougainvilliorum*
	Harris's hawk	*Parabuteo unicinctus*
	Helmeted Guineafowl	*Numida meleagris*

**Non-migratory**	House Sparrow	*Passer domesticus*
	Imperial cormorant	*Leucocarbo atriceps*
	Indian Peafowl	*Pavo cristatus*
	Kelp gull	*Larus dominicanus*
	Lesser horned owl	*Bubo magellanicus*
	Mandarin Duck	*Aix galericulata*
	Muscovy duck	*Cairina moschata*
	Mute swan	*Cygnus olor*
	Neotropic cormorant	*Nannopterum brasilianum*
	Peruvian booby	*Sula variegata*
	Red-footed Booby	*Sula sula*
	Red-gartered Coot	*Fulica armillata*
	Red-legged cormorant	*Poikilocarbo gaimardi*
	Roadside Hawk	*Rupornis magnirostris*
	Rock pigeon	*Columba livia*
	Rufous Hornero	*Furnarius rufus*
	Southern Lapwing	*Vanellus chilensis*
	Tawny eagle	*Aquila rapax*
	Tropical Screech-Owl	*Megascops choliba*
	White-faced whistling duck	*Dendrocygna viduata*
	Wood Duck	*Aix sponsa*

Supplementary Table S3. Pathogenicity classification of avian influenza A viruses used in this study based on the HA0 cleavage site.

ID	classification	RK_count	RK_runs	window_before_GLF_no_gaps	around_site_gapped
EPI_ISL_20179903	HPAI	6	KRRKR	SPLREKRRKR	TGLRNSPLREKRRKRGLFGAIAGF
EPI_ISL_18439563	HPAI	6	KRRKR	SPPREKRRKR	TGLRNSPPREKRRKRGLFGAKAGF
EPI_ISL_18439562	HPAI	6	KRRKR	SPLREKRRKR	TGLRNSPLREKRRKRGLFGAKAGF
EPI_ISL_18439564	HPAI	6	KRRKR	SPLREKRRKR	TGLRNSPLREKRRKRGLFGAIAGF
EPI_ISL_20179748	HPAI	6	KRRKR	SPLREKRRKR	TGLRNSPLREKRRKRGLFGAIAGF
EPI_ISL_20179753	HPAI	6	KRRKR	SPLREKRRKR	TGLRNSPLREKRRKRGLFGAIAGF
EPI_ISL_20151597	HPAI	6	KRRKR	SPLREKRRKR	TGLRNSPLREKRRKRGLFGAIAGF
EPI_ISL_20179757	HPAI	6	KRRKR	SPLREKRRKR	TGLRNSPLREKRRKRGLFGAIAGF
EPI_ISL_19645365	HPAI	6	RRRKR	SPLRERRRKR	TGLRNSPLRERRRKRGLFGAIAGF
EPI_ISL_20151596	HPAI	6	KRRKR	SPLREKRRKR	TGLRNSPLREKRRKRGLFGAIAGF
EPI_ISL_19745586	HPAI	6	RRRKR	SPLRERRRKR	TGLRNSPLRERRRKRGLFGAIAGF
EPI_ISL_19747213	HPAI	6	KRRKR	SPLREKRRKR	TGLRNSPLREKRRKRGLFGAIAGF
EPI_ISL_19747212	HPAI	6	KRRKR	SPLREKRRKR	TGLRNSPLREKRRKRGLFGAIAGF
EPI_ISL_19747215	HPAI	6	KRRKR	SPLREKRRKR	TGLRNSPLREKRRKRGLFGAIAGF
EPI_ISL_20180240	HPAI	6	KRRKR	SPLREKRRKR	TGLRNSPLREKRRKRGLFGAIAGF
EPI_ISL_19747214	HPAI	6	KRRKR	SPLREKRRKR	TGLRNSPLREKRRKRGLFGAIAGF
EPI_ISL_19747209	HPAI	6	KRRKR	SPLREKRRKR	TGLRNSPLREKRRKRGLFGAIAGF
EPI_ISL_19747208	HPAI	6	KRRKR	SPLREKRRKR	TGLRNSPLREKRRKRGLFGAIAGF
EPI_ISL_20180245	HPAI	6	KRRKR	SPLREKRRKR	TGLRNSPLREKRRKRGLFGAIAGF
EPI_ISL_19747211	HPAI	6	KRRKR	SPLREKRRKR	TGLRNSPLREKRRKRGLFGAIAGF
EPI_ISL_19747210	HPAI	6	KRRKR	SPLREKRRKR	TGLRNSPLREKRRKRGLFGAIAGF
EPI_ISL_19747205	HPAI	6	KRRKR	SPLREKRRKR	TGLRNSPLREKRRKRGLFGAIAGF
EPI_ISL_19747204	HPAI	6	KRRKR	SPLREKRRKR	TGLRNSPLREKRRKRGLFGAIAGF
EPI_ISL_20180249	HPAI	6	KRRKR	SPLREKRRKR	TGLRNSPLREKRRKRGLFGAIAGF
EPI_ISL_19747207	HPAI	6	KRRKR	SPLREKRRKR	TGLRNSPLREKRRKRGLFGAIAGF
EPI_ISL_19747206	HPAI	6	KRRKR	SPLREKRRKR	TGLRNSPLREKRRKRGLFGAIAGF
EPI_ISL_19747201	HPAI	6	KRRKR	SPLREKRRKR	TGLRNSPLREKRRKRGLFGAIAGF
EPI_ISL_19747200	HPAI	6	KRRKR	SPLREKRRKR	TGLRNSPLREKRRKRGLFGAIAGF
EPI_ISL_19747203	HPAI	6	KRRKR	SPLREKRRKR	TGLRNSPLREKRRKRGLFGAIAGF
EPI_ISL_19747202	HPAI	6	KRRKR	SPLREKRRKR	TGLRNSPLREKRRKRGLFGAIAGF
EPI_ISL_18592427	HPAI	6	KRRKR	SPLREKRRKR	TGLRNSPLREKRRKRGLFGAIAGF
EPI_ISL_18592426	HPAI	6	KRRKR	SPLREKRRKR	TGLRNSPLREKRRKRGLFGAIAGF
EPI_ISL_18592425	HPAI	6	KRRKR	SPLREKRRKR	TGLRNSPLREKRRKRGLFGAIAGF
EPI_ISL_18592424	HPAI	6	KRRKR	SPLREKRRKR	TGLRNSPLREKRRKRGLFGAIAGF
EPI_ISL_20180231	HPAI	6	KRRKR	SPLREKRRKR	TGLRNSPLREKRRKRGLFGAIAGF
EPI_ISL_18592429	HPAI	6	KRRKR	SPLREKRRKR	TGLRNSPLREKRRKRGLFGAIAGF

EPI_ISL_19589149	HPAI	6	KRRKR	SPLREKRRKR	TGLRNSPLREKRRKRGLFGAIAGF
EPI_ISL_18592428	HPAI	6	KRRKR	SPLREKRRKR	TGLRNSPLREKRRKRGLFGAIAGF
EPI_ISL_20182795	HPAI	6	KRRKR	SPLREKRRKR	TGLRNSPLREKRRKRGLFGAIAGF
EPI_ISL_20180235	HPAI	6	KRRKR	SPLREKRRKR	TGLRNSPLREKRRKRGLFGAIAGF
EPI_ISL_19747221	HPAI	6	KRRKR	SPLREKRRKR	TGLRNSPLREKRRKRGLFGAIAGF
EPI_ISL_19747220	HPAI	6	KRRKR	SPLREKRRKR	TGLRNSPLREKRRKRGLFGAIAGF
EPI_ISL_19879315	HPAI	6	KRRKR	SPLKEKRRKR	TGLRNSPLKEKRRKRGLFGAIAGF
EPI_ISL_19747217	HPAI	6	KRRKR	SPLREKRRKR	TGLRNSPLREKRRKRGLFGAIAGF
EPI_ISL_18592423	HPAI	6	KRRKR	SPLREKRRKR	TGLRNSPLREKRRKRGLFGAIAGF
EPI_ISL_19879314	HPAI	6	KRRKR	SPLREKRRKR	TGLRNSPLREKRRKRGLFGAIAGF
EPI_ISL_19747216	HPAI	6	KRRKR	SPLREKRRKR	TGLRNSPLREKRRKRGLFGAIAGF
EPI_ISL_18592422	HPAI	6	KRRKR	SPLREKRRKR	TGLRNSPLREKRRKRGLFGAIAGF
EPI_ISL_19879313	HPAI	6	KRRKR	SPLREKRRKR	TGLRNSPLREKRRKRGLFGAIAGF
EPI_ISL_19747219	HPAI	6	KRRKR	SPLREKRRKR	TGLRNSPLREKRRKRGLFGAIAGF
EPI_ISL_19747218	HPAI	6	KRRKR	SPLREKRRKR	TGLRNSPLREKRRKRGLFGAIAGF
EPI_ISL_19847535	HPAI	6	KRRKR	SPLREKRRKR	TGLRNSPLREKRRKRGLFGAIAGF
EPI_ISL_19747197	HPAI	6	KRRKR	SPLREKRRKR	TGLRNSPLREKRRKRGLFGAIAGF
EPI_ISL_18742221	HPAI	6	KRRKR	SPXREKRRKR	TGLRNSPXREKRRKRGLFGAKAGF
EPI_ISL_18742220	HPAI	6	KRRKR	SPLREKRRKR	TGLRNSPLREKRRKRGLFGAIAGF
EPI_ISL_19747199	HPAI	6	KRRKR	SPLREKRRKR	TGLRNSPLREKRRKRGLFGAIAGF
EPI_ISL_19747198	HPAI	6	KRRKR	SPLREKRRKR	TGLRNSPLREKRRKRGLFGAIAGF
EPI_ISL_18742217	HPAI	6	KRRKR	SPLREKRRKR	TGLRNSPLREKRRKRGLFGAKAGF
EPI_ISL_18742216	HPAI	6	KRRKR	SPLREKRRKR	TGLRNSPLREKRRKRGLFGAKAGF
EPI_ISL_18742219	HPAI	6	KRRKR	SPLREKRRKR	TGLRNSPLREKRRKRGLFGAKAGF
EPI_ISL_18742218	HPAI	6	KRRKR	SPLREKRRKR	TGLRNSPLREKRRKRGLFGAKAGF
EPI_ISL_18742213	HPAI	6	KRRKR	SPLREKRRKR	TGLRNSPLREKRRKRGLFGAKAGF
EPI_ISL_18742212	HPAI	6	KRRKR	SPLREKRRKR	TGLRNSPLREKRRKRGLFGAKAGF
EPI_ISL_18742215	HPAI	6	KRRKR	SPLREKRRKR	TGLRNSPLREKRRKRGLFGAKAGF
EPI_ISL_18742214	HPAI	6	KRRKR	SPLREKRRKR	TGLRNSPLREKRRKRGLFGAKAGF
EPI_ISL_19847538	HPAI	6	KRRKR	SPLREKRRKR	TGLRNSPLREKRRKRGLFGAIAGF
EPI_ISL_19847539	HPAI	6	KRRKR	SPLREKRRKR	TGLRNSPLREKRRKRGLFGAIAGF
EPI_ISL_19847536	HPAI	6	KRRKR	SPLREKRRKR	TGLRNSPLREKRRKRGLFGAIAGF
EPI_ISL_20180691	HPAI	6	KRRKR	SPLREKRRKR	TGLRNSPLREKRRKRGLFGAIAGF
EPI_ISL_20180690	HPAI	6	KRRKR	SPLREKRRKR	TGLRNSPLREKRRKRGLFGAIAGF
EPI_ISL_20180689	HPAI	6	KRRKR	SPLREKRRKR	TGLRNSPLREKRRKRGLFGAIAGF
EPI_ISL_20180688	HPAI	6	KRRKR	SPLREKRRKR	TGLRNSPLREKRRKRGLFGAIAGF
EPI_ISL_20180695	HPAI	6	KRRKR	SPLREKRRKR	TGLRNSPLREKRRKRGLFGAIAGF
EPI_ISL_20180694	HPAI	6	KRRKR	SPLREKRRKR	TGLRNSPLREKRRKRGLFGAIAGF
EPI_ISL_20180693	HPAI	6	KRRKR	SPLREKRRKR	TGLRNSPLREKRRKRGLFGAIAGF
EPI_ISL_20180692	HPAI	6	KRRKR	SPLREKRRKR	TGLRNSPLREKRRKRGLFGAIAGF
EPI_ISL_20180699	HPAI	6	KRRKR	SPLREKRRKR	TGLRNSPLREKRRKRGLFGAIAGF

EPI_ISL_20180698	HPAI	6	KRRKR	SPLKEKRRKR	TGLRNSPLKEKRRKRGLFGAIAGF
EPI_ISL_20180697	HPAI	6	KRRKR	SPLKEKRRKR	TGLRNSPLKEKRRKRGLFGAIAGF
EPI_ISL_20180696	HPAI	6	KRRKR	SPLREKRRKR	TGLRNSPLREKRRKRGLFGAIAGF
EPI_ISL_20180700	HPAI	6	KRRKR	SPLREKRRKR	TGLRNSPLREKRRKRGLFGAKAGF
EPI_ISL_20180047	HPAI	6	KRRKR	SPLREKRRKR	TGLRNSPLREKRRKRGLFGAIAGF
EPI_ISL_20180046	HPAI	6	KRRKR	SPLREKRRKR	TGLRNSPLREKRRKRGLFGAIAGF
EPI_ISL_19605291	HPAI	6	RRRKR	SPLRERRRKR	TGLRNSPLRERRRKRGLFGAIAGF
EPI_ISL_19490472	HPAI	6	RRRKR	SPLRERRRKR	TGLRNSPLRERRRKRGLFGAIAGF
EPI_ISL_17964848	HPAI	6	KRRKR	SPLREKRRKR	TGLRNSPLREKRRKRGLFGAIAGF
EPI_ISL_17964849	HPAI	6	RRRKR	SPLRERRRKR	TGLRNSPLRERRRKRGLFGAIAGF
EPI_ISL_19765893	HPAI	6	KRRKR	SPLREKRRKR	TGLRNSPLREKRRKRGLFGAIAGF
EPI_ISL_19605440	HPAI	6	RRRKR	SPLRERRRKR	TGLRNSPLRERRRKRGLFGAIAGF
EPI_ISL_19094638	HPAI	6	KRRKR	SPLREKRRKR	TGLRNSPLREKRRKRGLFGAIAGF
EPI_ISL_19606308	HPAI	6	KRRKR	SPLREKRRKR	TGLRNSPLREKRRKRGLFGAIAGF
EPI_ISL_19775581	HPAI	6	KRRKR	SPLREKRRKR	TGLRNSPLREKRRKRGLFGAIAGF
EPI_ISL_19154741	HPAI	6	RRRKR	SPLRERRRKR	TGLRNSPLRERRRKRGLFGAIAGF
EPI_ISL_18737454	HPAI	6	KRRKR	SPLREKRRKR	TGLRNSPLREKRRKRGLFGAIAGF
EPI_ISL_19303634	HPAI	6	KRRKR	SPLREKRRKR	TGLRNSPLREKRRKRGLFGAIAGF
EPI_ISL_19736856	HPAI	6	KRRKR	SPLREKRRKR	TGLRNSPLREKRRKRGLFGAIAGF
EPI_ISL_19604242	HPAI	6	KRRKR	SPLREKRRKR	TGLRNSPLREKRRKRGLFGAIAGF
EPI_ISL_19560339	HPAI	7	KRRKR	SRLREKRRKR	TGLRNSRLREKRRKRGLFGAIAGF
EPI_ISL_19594064	HPAI	6	KRRKR	SPLREKRRKR	TGLRNSPLREKRRKRGLFGAIAGF
EPI_ISL_19606295	HPAI	6	KRRKR	SPLREKRRKR	TGLRNSPLREKRRKRGLFGAIAGF
EPI_ISL_19303642	HPAI	6	KRRKR	SPLREKRRKR	TGLRNSPLREKRRKRGLFGAIAGF
EPI_ISL_19075358	HPAI	6	KRRKR	SPLREKRRKR	TGLRNSPLREKRRKRGLFGAIAGF
EPI_ISL_19606709	HPAI	6	KRRKR	SPLREKRRKR	TGLRNSPLREKRRKRGLFGAIAGF
EPI_ISL_17424631	HPAI	6	KRRKR	SPLREKRRKR	TGLRNSPLREKRRKRGLFGAIAGF
EPI_ISL_19775602	HPAI	6	RRRKR	SPLRERRRKR	TGLRNSPLRERRRKRGLFGAIAGF
EPI_ISL_18698525	HPAI	6	KRRKR	SPLREKRRKR	TGLRNSPLREKRRKRGLFGAIAGF
EPI_ISL_18698524	HPAI	6	KRRKR	SPLREKRRKR	TGLRNSPLREKRRKRGLFGAIAGF
EPI_ISL_18945315	HPAI	6	KRRKR	SPLREKRRKR	TGLRNSPLREKRRKRGLFGAIAGF
EPI_ISL_18698527	HPAI	6	KRRKR	NPLREKRRKR	TGLRNNPLREKRRKRGLFGAIAGF
EPI_ISL_19131168	HPAI	6	KRRKR	SPLREKRRKR	TGLRNSPLREKRRKRGLFGAIAGF
EPI_ISL_18698526	HPAI	6	KRRKR	SPLREKRRKR	TGLRNSPLREKRRKRGLFGAIAGF
EPI_ISL_18945317	HPAI	6	KRRKR	SPLREKRRKR	TGLRNSPLREKRRKRGLFGAIAGF
EPI_ISL_18698521	HPAI	6	KRRKR	SPLREKRRKR	TGLRNSPLREKRRKRGLFGAIAGF
EPI_ISL_17885972	HPAI	6	KRRKR	SPLREKRRKR	TGLRNSPLREKRRKRGLFGAIAGF
EPI_ISL_18945316	HPAI	6	KRRKR	SPLREKRRKR	TGLRNSPLREKRRKRGLFGAIAGF
EPI_ISL_18698520	HPAI	6	KRRKR	SPLREKRRKR	TGLRNSPLREKRRKRGLFGAIAGF
EPI_ISL_17885973	HPAI	6	KRRKR	SPLREKRRKR	TGLRNSPLREKRRKRGLFGAIAGF
EPI_ISL_18945319	HPAI	6	KRRKR	SPLREKRRKR	TGLRNSPLREKRRKRGLFGAIAGF

EPI_ISL_18698517	HPAI	6	KRRKR	SPLREKRRKR	TGLRNSPLREKRRKRGLFGAIAGF
EPI_ISL_17885976	HPAI	6	KRRKR	SPLREKRRKR	TGLRNSPLREKRRKRGLFGAIAGF
EPI_ISL_18945320	NA_no_GLF				
EPI_ISL_18698516	HPAI	6	KRRKR	SPLREKRRKR	TGLRNSPLREKRRKRGLFGAIAGF
EPI_ISL_18310942	HPAI	6	KRRKR	SPLREKRRKR	TGLRNSPLREKRRKRGLFGAIAGF
EPI_ISL_18698519	HPAI	6	KRRKR	SPLREKRRKR	TGLRNSPLREKRRKRGLFGAIAGF
EPI_ISL_17885978	HPAI	6	KRRKR	SPLREKRRKR	TGLRNSPLREKRRKRGLFGAIAGF
EPI_ISL_18698518	HPAI	6	KRRKR	SPLREKRRKR	TGLRNSPLREKRRKRGLFGAIAGF
EPI_ISL_18698513	HPAI	6	KRRKR	SPLREKRRKR	TGLRNSPLREKRRKRGLFGAIAGF
EPI_ISL_17885980	HPAI	6	KRRKR	SPLREKRRKR	TGLRNSPLREKRRKRGLFGAIAGF
EPI_ISL_18698512	HPAI	6	KRRKR	SPLREKRRKR	TGLRNSPLREKRRKRGLFGAIAGF
EPI_ISL_18698515	HPAI	6	KRRKR	SPLREKRRKR	TGLRNSPLREKRRKRGLFGAIAGF
EPI_ISL_17885982	HPAI	6	KRRKR	SPLREKRRKR	TGLRNSPLREKRRKRGLFGAIAGF
EPI_ISL_18698514	HPAI	6	KRRKR	SPLREKRRKR	TGLRNSPLREKRRKRGLFGAIAGF
EPI_ISL_17885983	HPAI	6	KRRKR	SPLREKRRKR	TGLRNSPLREKRRKRGLFGAIAGF
EPI_ISL_17885952	HPAI	6	KRRKR	SPLREKRRKR	TGLRNSPLREKRRKRGLFGAIAGF
EPI_ISL_18698508	HPAI	6	KRRKR	SPLREKRRKR	TGLRNSPLREKRRKRGLFGAIAGF
EPI_ISL_18698511	HPAI	6	KRRKR	SPLREKRRKR	TGLRNSPLREKRRKRGLFGAIAGF
EPI_ISL_17885954	HPAI	6	KRRKR	SPLREKRRKR	TGLRNSPLREKRRKRGLFGAIAGF
EPI_ISL_18698510	HPAI	6	KRRKR	SPLREKRRKR	TGLRNSPLREKRRKRGLFGAIAGF
EPI_ISL_17885955	HPAI	6	KRRKR	SPLREKRRKR	TGLRNSPLREKRRKRGLFGAIAGF
EPI_ISL_18755338	HPAI	6	KRKKR	SPLREKRKKR	TGLRNSPLREKRKKRGLFGAIAGF
EPI_ISL_18698505	HPAI	6	KRRKR	SPLREKRRKR	TGLRNSPLREKRRKRGLFGAIAGF
EPI_ISL_17885956	HPAI	6	KRRKR	SPLREKRRKR	TGLRNSPLREKRRKRGLFGAIAGF
EPI_ISL_18698504	HPAI	6	KRRKR	SPLREKRRKR	TGLRNSPLREKRRKRGLFGAIAGF
EPI_ISL_18698507	HPAI	6	KRRKR	SPLREKRRKR	TGLRNSPLREKRRKRGLFGAIAGF
EPI_ISL_17885958	HPAI	6	KRRKR	SPLREKRRKR	TGLRNSPLREKRRKRGLFGAIAGF
EPI_ISL_18698506	HPAI	6	KRRKR	SPLREKRRKR	TGLRNSPLREKRRKRGLFGAIAGF
EPI_ISL_17885962	HPAI	6	KRRKR	SPLREKRRKR	TGLRNSPLREKRRKRGLFGAIAGF
EPI_ISL_18698502	HPAI	6	KRRKR	SPLREKRRKR	TGLRNSPLREKRRKRGLFGAIAGF
EPI_ISL_18698497	HPAI	6	KRRKR	SPLREKRRKR	TGLRNSPLREKRRKRGLFGAIAGF
EPI_ISL_17885964	HPAI	6	KRRKR	SPLREKRRKR	TGLRNSPLREKRRKRGLFGAIAGF
EPI_ISL_17885965	HPAI	6	KRRKR	SPLREKRRKR	TGLRNSPLREKRRKRGLFGAIAGF
EPI_ISL_17885966	HPAI	6	KRRKR	SPLREKRRKR	TGLRNSPLREKRRKRGLFGAIAGF
EPI_ISL_19131196	HPAI	6	KRRKR	SPLREKRRKR	TGLRNSPLREKRRKRGLFGAVAGF
EPI_ISL_19781426	HPAI	6	KRRKR	SPLREKRRKR	TGLRNSPLREKRRKRGLFGAIAGF
EPI_ISL_17885967	HPAI	6	KRRKR	SPLREKRRKR	TGLRNSPLREKRRKRGLFGAIAGF
EPI_ISL_18310967	HPAI	6	KRRKR	SPLREKRRKR	TGLRNSPLREKRRKRGLFGAIAGF
EPI_ISL_18310966	HPAI	6	KRRKR	SPLREKRRKR	TGLRNSPLREKRRKRGLFGAIAGF
EPI_ISL_18310965	HPAI	6	KRRKR	SPLREKRRKR	TGLRNSPLREKRRKRGLFGAIAGF
EPI_ISL_18310964	HPAI	6	KRRKR	SPLREKRRKR	TGLRNSPLREKRRKRGLFGAIAGF

EPI_ISL_18310963	HPAI	6	KRRKR	SPLREKRRKR	TGLRNSPLREKRRKRGLFGAIAGF
EPI_ISL_18310962	HPAI	6	KRRKR	SPLREKRRKR	TGLRNSPLREKRRKRGLFGAIAGF
EPI_ISL_18310961	HPAI	6	KRRKR	SPLREKRRKR	TGLRNSPLREKRRKRGLFGAIAGF
EPI_ISL_18310960	HPAI	6	KRRKR	SPLREKRRKR	TGLRNSPLREKRRKRGLFGAIAGF
EPI_ISL_17353507	HPAI	6	KRRKR	SPLREKRRKR	TGLRNSPLREKRRKRGLFGAIAGF
EPI_ISL_17353508	HPAI	6	KRRKR	SPLREKRRKR	TGLRNSPLREKRRKRGLFGAIAGF
EPI_ISL_17353509	HPAI	6	KRRKR	SPLREKRRKR	TGLRNSPLREKRRKRGLFGAIAGF
EPI_ISL_17353510	HPAI	6	KRRKR	SPLREKRRKR	TGLRNSPLREKRRKRGLFGAIAGF
EPI_ISL_17012018	HPAI	6	KRRKR	SPLREKRRKR	TGLRNSPLREKRRKRGLFGAIAGF
EPI_ISL_19131159	HPAI	6	KRRKR	SPLREKRRKR	TGLRNSPLREKRRKRGLFGAIAGF
EPI_ISL_19131156	HPAI	6	KRRKR	SPLREKRRKR	TGLRNSPLREKRRKRGLFGAIAGF
EPI_ISL_19836182	HPAI	6	KRRKR	SPLREKRRKR	TGLRNSPLREKRRKRGLFGAIAGF
EPI_ISL_19131163	HPAI	6	KRRKR	SPLREKRRKR	TGLRNSPLREKRRKRGLFGAIAGF
EPI_ISL_18310959	HPAI	6	KRRKR	SPLREKRRKR	TGLRNSPLREKRRKRGLFGAIAGF
EPI_ISL_19131162	HPAI	6	KRRKR	SPLREKRRKR	TGLRNSPLREKRRKRGLFGAIAGF
EPI_ISL_18310958	HPAI	6	KRRKR	SPLREKRRKR	TGLRNSPLREKRRKRGLFGAIAGF
EPI_ISL_18310957	HPAI	6	KRRKR	SPLREKRRKR	TGLRNSPLREKRRKRGLFGAIAGF
EPI_ISL_20078350	HPAI	6	KRRKR	SPLREKRRKR	TGLRNSPLREKRRKRGLFGAIAGF
EPI_ISL_18698529	HPAI	6	KRRKR	SPLREKRRKR	TGLRNSPLREKRRKRGLFGAIAGF
EPI_ISL_19131166	HPAI	6	KRRKR	SPLREKRRKR	TGLRNSPLREKRRKRGLFGAIAGF
EPI_ISL_18698528	HPAI	6	KRRKR	SPLREKRRKR	TGLRNSPLREKRRKRGLFGAIAGF
EPI_ISL_19131164	HPAI	6	KRRKR	SPLREKRRKR	TGLRNSPLREKRRKRGLFGAIAGF
EPI_ISL_18698530	HPAI	6	KRRKR	SPLREKRRKR	TGLRNSPLREKRRKRGLFGAIAGF
EPI_ISL_19410280	HPAI	6	KRRKR	SPLREKRRKR	TGLRNSPLREKRRKRGLFGAIAGF
EPI_ISL_18265430	HPAI	6	KRRKR	SPLREKRRKR	TGLRNSPLREKRRKRGLFGAIAGF
EPI_ISL_19410281	HPAI	6	KRRKR	SPLREKRRKR	TGLRNSPLREKRRKRGLFGAIAGF
EPI_ISL_18265431	HPAI	6	KRRKR	SPLREKRRKR	TGLRNSPLREKRRKRGLFGAIAGF
EPI_ISL_19410283	HPAI	6	KRRKR	SPLREKRRKR	TGLRNSPLREKRRKRGLFGAIAGF
EPI_ISL_19410284	HPAI	6	KRRKR	SPLREKRRKR	TGLRNSPLREKRRKRGLFGAIAGF
EPI_ISL_19410285	HPAI	6	KRRKR	SPLREKRRKR	TGLRNSPLREKRRKRGLFGAIAGF
EPI_ISL_19215200	HPAI	6	KRKKR	SPLREKRKKR	TGLRNSPLREKRKKRGLFGAIAGF
EPI_ISL_19410286	HPAI	6	KRRKR	SPLREKRRKR	TGLRNSPLREKRRKRGLFGAIAGF
EPI_ISL_19410272	HPAI	6	KRRKR	SPLREKRRKR	TGLRNSPLREKRRKRGLFGAIAGF
EPI_ISL_18265436	HPAI	6	KRRKR	SPLREKRRKR	TGLRNSPLREKRRKRGLFGAIAGF
EPI_ISL_19410275	LPAI	0		TTWNVTWNNQ	QIFPDTTWNVTWNNQGLFKLILPE
EPI_ISL_18522961	HPAI	6	KRRKR	SPLREKRRKR	TGLRNSPLREKRRKRGLFGAKAGF
EPI_ISL_18265437	NA_no_GLF				
EPI_ISL_18265434	HPAI	6	KRRKR	SPLREKRRKR	TGLRNSPLREKRRKRGLFGAIAGF
EPI_ISL_19410277	HPAI	6	KRRKR	SPLREKRRKR	TGLRNSPLREKRRKRGLFGAIAGF
EPI_ISL_18265435	HPAI	6	KRRKR	SPLREKRRKR	TGLRNSPLREKRRKRGLFGAIAGF
EPI_ISL_18265432	HPAI	6	KRRKR	SPLREKRRKR	TGLRNSPLREKRRKRGLFGAIAGF

EPI_ISL_19410279	HPAI	6	KRRKR	SPLREKRRKR	TGLRNSPLREKRRKRGLFGAIAGF
EPI_ISL_18265433	HPAI	6	KRRKR	SPLREKRRKR	TGLRNSPLREKRRKRGLFGAIAGF
EPI_ISL_19418491	HPAI	6	KRRKR	SPLREKRRKR	TGLRNSPLREKRRKRGLFGAIAGF
EPI_ISL_19410299	HPAI	6	KRRKR	SPLREKRRKR	TGLRNSPLREKRRKRGLFGAIAGF
EPI_ISL_19410300	HPAI	6	KRRKR	SPLREKRRKR	TGLRNSPLREKRRKRGLFGAIAGF
EPI_ISL_18265422	NA_no_GLF				
EPI_ISL_16631973	HPAI	6	RRRKR	SPLRERRRKR	TGLRNSPLRERRRKRGLFGAIAGF
EPI_ISL_19418487	HPAI	6	KRRKR	SPLREKRRKR	TGLRNSPLREKRRKRGLFGAIAGF
EPI_ISL_18760063	HPAI	6	KRRKR	SPLREKRRKR	TGLRNSPLREKRRKRGLFGAIAGF
EPI_ISL_19215175	HPAI	6	KRKKR	SPLREKRKKR	TGLRNSPLREKRKKRGLFGAIAGF
EPI_ISL_18760060	HPAI	6	KRRKR	SPLREKRRKR	TGLRNSPLREKRRKRGLFGAIAGF
EPI_ISL_18760061	HPAI	6	KRRKR	SPLREKRRKR	TGLRNSPLREKRRKRGLFGAIAGF
EPI_ISL_19215181	HPAI	6	KRKKR	SPLREKRKKR	TGLRNSPLREKRKKRGLFGAIAGF
EPI_ISL_19215180	HPAI	6	KRRKR	SPLREKRRKR	TGLRNSPLREKRRKRGLFGAIAGF
EPI_ISL_18939722	HPAI	6	KRRKR	SPLREKRRKR	TGLRNSPLREKRRKRGLFGAIAGF
EPI_ISL_19215183	HPAI	6	KRKKR	SPLREKRKKR	TGLRNSPLREKRKKRGLFGAIAGF
EPI_ISL_19215182	HPAI	6	KRRKR	SPLREKRRKR	TGLRNSPLREKRRKRGLFGAIAGF
EPI_ISL_19215177	HPAI	6	KRKKR	SPLREKRKKR	TGLRNSPLREKRKKRGLFGAIAGF
EPI_ISL_19215176	HPAI	6	KRRKR	SPLREKRRKR	TGLRNSPLREKRRKRGLFGAIAGF
EPI_ISL_19215179	HPAI	6	KRRKR	SPLREKRRKR	TGLRNSPLREKRRKRGLFGAIAGF
EPI_ISL_19215178	HPAI	7	KRRKR	RPLREKRRKR	TGLRNRPLREKRRKRGLFGAIAGF
EPI_ISL_19410264	HPAI	6	KRRKR	SPLREKRRKR	TGLRNSPLREKRRKRGLFGAIAGF
EPI_ISL_19215189	HPAI	6	KRKKR	SPLREKRKKR	TGLRNSPLREKRKKRGLFGAIAGF
EPI_ISL_19410265	HPAI	6	KRRKR	SPLREKRRKR	TGLRNSPLREKRRKRGLFGAIAGF
EPI_ISL_19215188	HPAI	6	KRKKR	SPLREKRKKR	TGLRNSPLREKRKKRGLFGAIAGF
EPI_ISL_19410266	HPAI	6	KRRKR	SPLREKRRKR	TGLRNSPLREKRRKRGLFGAIAGF
EPI_ISL_19215191	HPAI	6	KRKKR	SPLREKRKKR	TGLRNSPLREKRKKRGLFGAIAGF
EPI_ISL_19070288	HPAI	6	KRRKR	SPLREKRRKR	TGLRNSPLREKRRKRGLFGAIAGF
EPI_ISL_17777533	HPAI	6	KRRKR	SPLREKRRKR	TGLRNSPLREKRRKRGLFGAKAGF
EPI_ISL_19410267	HPAI	6	KRRKR	SPLREKRRKR	TGLRNSPLREKRRKRGLFGAIAGF
EPI_ISL_19215190	HPAI	6	KRKKR	SPLREKRKKR	TGLRNSPLREKRKKRGLFGAIAGF
EPI_ISL_17777532	HPAI	6	KRRKR	SPLREKRRKR	TGLRNSPLREKRRKRGLFGAKAGF
EPI_ISL_19410268	HPAI	6	KRRKR	SPLREKRRKR	TGLRNSPLREKRRKRGLFGAIAGF
EPI_ISL_19215185	HPAI	6	KRKKR	SPLREKRKKR	TGLRNSPLREKRKKRGLFGAIAGF
EPI_ISL_18054503	HPAI	6	KRRKR	SPLREKRRKR	TGLRNSPLREKRRKRGLFGAIAGF
EPI_ISL_17777531	HPAI	6	KRRKR	SPLREKRRKR	TGLRNSPLREKRRKRGLFGAKAGF
EPI_ISL_19410269	HPAI	6	KRRKR	SPLREKRRKR	TGLRNSPLREKRRKRGLFGAIAGF
EPI_ISL_19215184	HPAI	6	KRKKR	SPLREKRKKR	TGLRNSPLREKRKKRGLFGAIAGF
EPI_ISL_18054502	HPAI	6	KRRKR	SPLREKRRKR	TGLRNSPLREKRRKRGLFGAIAGF
EPI_ISL_17777530	HPAI	6	KRRKR	SPLREKRRKR	TGLRNSPLREKRRKRGLFGAIAGF
EPI_ISL_19215187	HPAI	6	KRKKR	SPLREKRKKR	TGLRNSPLREKRKKRGLFGAIAGF

EPI_ISL_18054501	HPAI	6	KRRKR	SPLREKRRKR	TGLRNSPLREKRRKRGLFGAIAGF
EPI_ISL_17777529	HPAI	6	KRRKR	SPLREKRRKR	TGLRNSPLREKRRKRGLFGAIAGF
EPI_ISL_19410271	HPAI	6	KRRKR	SPLREKRRKR	TGLRNSPLREKRRKRGLFGAIAGF
EPI_ISL_19215186	HPAI	6	KRKKR	SPLREKRKKR	TGLRNSPLREKRKKRGLFGAIAGF
EPI_ISL_18054500	HPAI	6	KRRKR	SPLREKRRKR	TGLRNSPLREKRRKRGLFGAIAGF
EPI_ISL_17777528	HPAI	6	KRRKR	SPLREKRRKR	TGLRNSPLREKRRKRGLFGAIAGF
EPI_ISL_19215197	HPAI	6	KRKKR	SPLREKRKKR	TGLRNSPLREKRKKRGLFGAIAGF
EPI_ISL_18054507	HPAI	6	KRRKR	SPLREKRRKR	TGLRNSPLREKRRKRGLFGAIAGF
EPI_ISL_17777527	HPAI	6	KRRKR	SPLREKRRKR	TGLRNSPLREKRRKRGLFGAIAGF
EPI_ISL_19215196	HPAI	6	KRKKR	SPLREKRKKR	TGLRNSPLREKRKKRGLFGAIAGF
EPI_ISL_18054506	HPAI	6	KRRKR	SPLREKRRKR	TGLRNSPLREKRRKRGLFGAIAGF
EPI_ISL_17777526	NA_no_GLF				
EPI_ISL_19215199	HPAI	6	KRKKR	SPLREKRKKR	TGLRNSPLREKRKKRGLFGAIAGF
EPI_ISL_17777525	HPAI	6	KRRKR	SPLREKRRKR	TGLRNSPLREKRRKRGLFGAIAGF
EPI_ISL_19215198	HPAI	6	KRKKR	SPLREKRKKR	TGLRNSPLREKRKKRGLFGAIAGF
EPI_ISL_18909019	HPAI	6	RRRKR	SPLRERRRKR	TGLRNSPLRERRRKRGLFGAIAGF
EPI_ISL_18054504	HPAI	6	KRRKR	SPLREKRRKR	TGLRNSPLREKRRKRGLFGAIAGF
EPI_ISL_19215193	HPAI	6	KRKKR	SPLREKRKKR	TGLRNSPLREKRKKRGLFGAIAGF
EPI_ISL_19410261	HPAI	6	KRRKR	SPLREKRRKR	TGLRNSPLREKRRKRGLFGAIAGF
EPI_ISL_19215192	HPAI	6	KRKKR	SPLREKRKKR	TGLRNSPLREKRKKRGLFGAIAGF
EPI_ISL_18054510	NA_no_GLF				
EPI_ISL_19410262	HPAI	6	KRRKR	SPLREKRRKR	TGLRNSPLREKRRKRGLFGAIAGF
EPI_ISL_19215195	HPAI	6	KRKKR	SPLREKRKKR	TGLRNSPLREKRKKRGLFGAIAGF
EPI_ISL_18054509	HPAI	6	KRRKR	SPLREKRRKR	TGLRNSPLREKRRKRGLFGAIAGF
EPI_ISL_19410263	HPAI	6	KRRKR	SPLREKRRKR	TGLRNSPLREKRRKRGLFGAIAGF
EPI_ISL_19215194	HPAI	6	KRKKR	SPLREKRKKR	TGLRNSPLREKRKKRGLFGAIAGF
EPI_ISL_18497946	HPAI	6	KRRKR	SPLREKRRKR	TGLRNSPLREKRRKRGLFGAKAGF
EPI_ISL_18497950	NA_no_GLF				
EPI_ISL_19466158	HPAI	6	KRRKR	SPLREKRRKR	TGLRNSPLREKRRKRGLFGAIAGF
EPI_ISL_19752381	HPAI	6	KRRKR	SPLREKRRKR	TGLRNSPLREKRRKRGLFGAIAGF
EPI_ISL_18760074	HPAI	6	KRRKR	SPLREKRRKR	TGLRNSPLREKRRKRGLFGAIAGF
EPI_ISL_18760073	HPAI	6	KRRKR	SPLREKRRKR	TGLRNSPLREKRRKRGLFGAIAGF
EPI_ISL_18760069	HPAI	6	KRRKR	SPLREKRRKR	TGLRNSPLREKRRKRGLFGAIAGF
EPI_ISL_18760067	HPAI	6	KRRKR	SPLREKRRKR	TGLRNSPLREKRRKRGLFGAIAGF
EPI_ISL_19139974	HPAI	6	KRRKR	SPLREKRRKR	TGLRNSPLREKRRKRGLFGAIAGF
EPI_ISL_19391463	HPAI	6	KRRKR	SPLREKRRKR	TGLRNSPLREKRRKRGLFGAIAGF
EPI_ISL_19391462	HPAI	6	KRRKR	SPLREKRRKR	TGLRNSPLREKRRKRGLFGAIAGF
EPI_ISL_19391461	HPAI	6	KRRKR	SPLREKRRKR	TGLRNSPLREKRRKRGLFGAIAGF
EPI_ISL_19391460	HPAI	6	KRRKR	SPLREKRRKR	TGLRNSPLREKRRKRGLFGAIAGF
EPI_ISL_19391459	HPAI	6	KRRKR	SPLREKRRKR	TGLRNSPLREKRRKRGLFGAIAGF
EPI_ISL_19391458	HPAI	6	KRRKR	SPLREKRRKR	TGLRNSPLREKRRKRGLFGAIAGF

EPI_ISL_19466210	HPAI	6	KRRKR	SPLREKRRKR	TGLRNSPLREKRRKRGLFGAIAGF
EPI_ISL_17468386	HPAI	6	KRRKR	SPLREKRRKR	TGLRNSPLREKRRKRGLFGAIAGF
EPI_ISL_19466181	HPAI	6	KRRKR	SPLREKRRKR	TGLRNSPLREKRRKRGLFGAIAGF
EPI_ISL_18698732	HPAI	6	KRRKR	SPLREKRRKR	TGLRNSPLREKRRKRGLFGAIAGF
EPI_ISL_19823067	HPAI	6	KRRKR	SPLREKRRKR	TGLRNSPLREKRRKRGLFGAIAGF
EPI_ISL_19823064	HPAI	6	KRRKR	SPLREKRRKR	TGLRNSPLREKRRKRGLFGAIAGF
EPI_ISL_18698731	HPAI	6	KRRKR	SPLREKRRKR	TGLRNSPLREKRRKRGLFGAIAGF
EPI_ISL_18698730	HPAI	6	KRRKR	SPLREKRRKR	TGLRNSPLREKRRKRGLFGAIAGF
EPI_ISL_19823065	HPAI	6	KRRKR	SPLREKRRKR	TGLRNSPLREKRRKRGLFGAIAGF
EPI_ISL_19070498	HPAI	6	KRRKR	SPLREKRRKR	TGLRNSPLREKRRKRGLFGAIAGF
EPI_ISL_16891400	HPAI	6	KRRKR	SPLREKRRKR	TGLRNSPLREKRRKRGLFGAIAGF
EPI_ISL_16891402	HPAI	6	KRRKR	SPLREKRRKR	TGLRNSPLREKRRKRGLFGAIAGF
EPI_ISL_19151400	HPAI	6	KRRKR	SPLREKRRKR	TGLRNSPLREKRRKRGLFGAIAGF
EPI_ISL_18698755	HPAI	6	KRRKR	SPLREKRRKR	TGLRNSPLREKRRKRGLFGAIAGF
EPI_ISL_18698754	HPAI	6	KRRKR	SPLREKRRKR	TGLRNSPLREKRRKRGLFGAIAGF
EPI_ISL_19466252	HPAI	6	KRRKR	SPLREKRRKR	TGLRNSPLREKRRKRGLFGAIAGF
EPI_ISL_19070482	HPAI	6	KRRKR	SPLREKRRKR	TGLRNSPLREKRRKRGLFGAIAGF
EPI_ISL_19070483	HPAI	6	KRRKR	SPLREKRRKR	TGLRNSPLREKRRKRGLFGAIAGF
EPI_ISL_19070481	HPAI	6	KRRKR	SPLREKRRKR	TGLRNSPLREKRRKRGLFGAIAGF
EPI_ISL_20267521	HPAI	6	KRRKR	SPLREKRRKR	TGLRNSPLREKRRKRGLFGAIAGF
EPI_ISL_19070486	HPAI	6	KRRKR	SPLREKRRKR	TGLRNSPLREKRRKRGLFGAIAGF
EPI_ISL_20267523	HPAI	6	KRRKR	SPLREKRRKR	TGLRNSPLREKRRKRGLFGAIAGF
EPI_ISL_20267522	HPAI	6	KRRKR	SPLREKRRKR	TGLRNSPLREKRRKRGLFGAIAGF
EPI_ISL_19070485	HPAI	6	KRRKR	SPLREKRRKR	TGLRNSPLREKRRKRGLFGAIAGF
EPI_ISL_19070492	HPAI	6	KRRKR	SPLREKRRKR	TGLRNSPLREKRRKRGLFGAIAGF
EPI_ISL_18371664	HPAI	6	KRRKR	SPLREKRRKR	TGLRNSPLREKRRKRGLFGAIAGF
EPI_ISL_18371665	HPAI	6	KRRKR	SPLREKRRKR	TGLRNSPLREKRRKRGLFGAIAGF
EPI_ISL_18371666	HPAI	6	KRRKR	SPLREKRRKR	TGLRNSPLREKRRKRGLFGAIAGF
EPI_ISL_16013752	HPAI	6	RRRKR	SPLRERRRKR	TGLRNSPLRERRRKRGLFGAIAGF
EPI_ISL_19162729	HPAI	6	KRRKR	SPLREKRRKR	TGLRNSPLREKRRKRGLFGAIAGF
EPI_ISL_16249274	NA_no_GLF				
EPI_ISL_16013753	HPAI	6	RRRKR	SPLRERRRKR	TGLRNSPLRERRRKRGLFGAIAGF
EPI_ISL_19404902	HPAI	6	KRRKR	SPLREKRRKR	TGLRNSPLREKRRKRGLFGAIAGF
EPI_ISL_19162731	HPAI	6	KRRKR	SPLREKRRKR	TGLRNSPLREKRRKRGLFGAVAGF
EPI_ISL_19137653	HPAI	6	KRRKR	SPLREKRRKR	TGLRNSPLREKRRKRGLFGAIAGF
EPI_ISL_18137671	LPAI	2	KR	XXXXXXXXKR	XXXXXXXXXXXXXKRGLFGAIAGF
EPI_ISL_19404916	HPAI	6	KRRKR	SPLREKRRKR	TGLRNSPLREKRRKRGLFGAIAGF
EPI_ISL_18931268	HPAI	6	RRRKR	SPLRERRRKR	TGLRNSPLRERRRKRGLFGAIAGF
EPI_ISL_17353838	HPAI	6	KRRKR	SPLREKRRKR	TGLRNSPLREKRRKRGLFGAIAGF
EPI_ISL_18931271	HPAI	6	RRRKR	SPLRERRRKR	TGLRNSPLRERRRKRGLFGAIAGF
EPI_ISL_17353839	HPAI	6	KRRKR	SPLREKRRKR	TGLRNSPLREKRRKRGLFGAIAGF

EPI_ISL_18931272	HPAI	6	RRRKR	SPLRERRRKR	TGLRNSPLRERRRKRGLFGAIAGF
EPI_ISL_18931274	HPAI	6	RRRKR	SPLRERRRKR	TGLRNSPLRERRRKRGLFGAIAGF
EPI_ISL_18931276	HPAI	6	RRRKR	SPLRERRRKR	TGLRNSPLRERRRKRGLFGAIAGF
EPI_ISL_16701840	HPAI	6	RRRKR	SPLRERRRKR	TGLRNSPLRERRRKRGLFGAIAGF
EPI_ISL_16249730	HPAI	6	KRRKR	SPLREKRRKR	TGLRNSPLREKRRKRGLFGAKAGF
EPI_ISL_18217104	HPAI	6	KRKKR	SPLREKRKKR	TGLRNSPLREKRKKRGLFGAIAGF
EPI_ISL_17885849	HPAI	6	KRRKR	SPLREKRRKR	TGLRNSPLREKRRKRGLFGAIAGF
EPI_ISL_19167924	HPAI	6	KRRKR	SPLREKRRKR	TGLRNSPLREKRRKRGLFGAIAGF
EPI_ISL_19167925	HPAI	6	KRRKR	SPLREKRRKR	TGLRNSPLREKRRKRGLFGAIAGF
EPI_ISL_19167926	HPAI	6	KRRKR	SPLREKRRKR	TGLRNSPLREKRRKRGLFGAIAGF
EPI_ISL_17885872	HPAI	6	KRRKR	SPLREKRRKR	TGLRNSPLREKRRKRGLFGAIAGF
EPI_ISL_17805999	HPAI	6	KRRKR	SPLRGKRRKR	TGLRNSPLRGKRRKRGLFGAIAGF
EPI_ISL_17885873	HPAI	6	KRRKR	SPLREKRRKR	TGLRNSPLREKRRKRGLFGAIAGF
EPI_ISL_17805998	HPAI	6	KRRKR	SPLREKRRKR	TGLRNSPLREKRRKRGLFGAIAGF
EPI_ISL_17885874	HPAI	6	KRRKR	SPLREKRRKR	TGLRNSPLREKRRKRGLFGAIAGF
EPI_ISL_17805997	HPAI	6	KRRKR	SPLREKRRKR	TGLRNSPLREKRRKRGLFGAIAGF
EPI_ISL_17805996	HPAI	6	KRRKR	SPLREKRRKR	TGLRNSPLREKRRKRGLFGAIAGF
EPI_ISL_17885876	HPAI	6	KRRKR	SPLREKRRKR	TGLRNSPLREKRRKRGLFGAIAGF
EPI_ISL_17805995	HPAI	6	KRRKR	SPLREKRRKR	TGLRNSPLREKRRKRGLFGAIAGF
EPI_ISL_17660073	HPAI	6	KRRKR	SPLREKRRKR	TGLRNSPLREKRRKRGLFGAIAGF
EPI_ISL_17885877	HPAI	6	KRRKR	SPLREKRRKR	TGLRNSPLREKRRKRGLFGAIAGF
EPI_ISL_17805994	HPAI	6	KRRKR	SPLREKRRKR	TGLRNSPLREKRRKRGLFGAIAGF
EPI_ISL_17660072	HPAI	6	KRRKR	SPLREKRRKR	TGLRNSPLREKRRKRGLFGAIAGF
EPI_ISL_17885878	HPAI	6	KRRKR	SPLREKRRKR	TGLRNSPLREKRRKRGLFGAIAGF
EPI_ISL_16249681	HPAI	6	KRRKR	SPLREKRRKR	TGLRNSPLREKRRKRGLFGAKAGF
EPI_ISL_19174531	HPAI	6	KRRKR	SPLREKRRKR	TGLRNSPLREKRRKRGLFGAIAGF
EPI_ISL_17660074	HPAI	6	KRRKR	SPLREKRRKR	TGLRNSPLREKRRKRGLFGAIAGF
EPI_ISL_17805991	HPAI	6	KRRKR	SPLREKRRKR	TGLRNSPLREKRRKRGLFGAIAGF
EPI_ISL_17805990	HPAI	6	KRRKR	SPLREKRRKR	TGLRNSPLREKRRKRGLFGAIAGF
EPI_ISL_18130622	HPAI	6	KRKKR	SPLREKRKKR	TGLRNSPLREKRKKRGLFGAIAGF
EPI_ISL_17805989	HPAI	6	KRRKR	SPLREKRRKR	TGLRNSPLREKRRKRGLFGAIAGF
EPI_ISL_17660071	HPAI	6	KRRKR	SPLREKRRKR	TGLRNSPLREKRRKRGLFGAIAGF
EPI_ISL_19404931	HPAI	6	KRRKR	SPLREKRRKR	TGLRNSPLREKRRKRGLFGAIAGF
EPI_ISL_17805988	HPAI	6	KRRKR	SPLREKRRKR	TGLRNSPLREKRRKRGLFGAIAGF
EPI_ISL_17660070	HPAI	6	KRRKR	SPLREKRRKR	TGLRNSPLREKRRKRGLFGAIAGF
EPI_ISL_17805987	HPAI	6	KRRKR	SPLREKRRKR	TGLRNSPLREKRRKRGLFGAIAGF
EPI_ISL_18975372	HPAI	6	KRKKR	SPLREKRKKR	TGLRNSPLREKRKKRGLFGAIAGF
EPI_ISL_18755251	HPAI	6	KRKKR	SPLREKRKKR	TGLRNSPLREKRKKRGLFGAIAGF
EPI_ISL_17805986	HPAI	6	KRRKR	SPLREKRRKR	TGLRNSPLREKRRKRGLFGAIAGF
EPI_ISL_18130597	HPAI	6	KRKKR	SPLREKRKKR	TGLRNSPLREKRKKRGLFGAIAGF
EPI_ISL_17099964	HPAI	6	KRRKR	SPLREKRRKR	TGLRNSPLREKRRKRGLFGAIAGF

EPI_ISL_17806003	HPAI	6	KRRKR	SPLREKRRKR	TGLRNSPLREKRRKRGLFGAIAGF
EPI_ISL_17885869	HPAI	6	KRRKR	SPLREKRRKR	TGLRNSPLREKRRKRGLFGAIAGF
EPI_ISL_17806002	HPAI	6	KRRKR	SPLREKRRKR	TGLRNSPLREKRRKRGLFGAIAGF
EPI_ISL_17885870	HPAI	6	KRRKR	SPLREKRRKR	TGLRNSPLREKRRKRGLFGAIAGF
EPI_ISL_17806001	HPAI	6	KRRKR	SPLREKRRKR	TGLRNSPLREKRRKRGLFGAIAGF
EPI_ISL_17885871	HPAI	6	KRRKR	SPLREKRRKR	TGLRNSPLREKRRKRGLFGAIAGF
EPI_ISL_17806000	HPAI	6	KRRKR	SPLREKRRKR	TGLRNSPLREKRRKRGLFGAIAGF
EPI_ISL_19765485	HPAI	6	KRRKR	SPLREKRRKR	TGLRNSPLREKRRKRGLFGAIAGF
EPI_ISL_17973458	HPAI	4	RRKR	XXXXXXRRKR	XXXXXXXXXXXRRKRGLFGAIAGF
EPI_ISL_18698460	HPAI	6	KRRKR	SPLREKRRKR	TGLRNSPLREKRRKRGLFGAIAGF
EPI_ISL_18698462	HPAI	6	KRRKR	SPLREKRRKR	TGLRNSPLREKRRKRGLFGAIAGF
EPI_ISL_18698459	HPAI	6	KRRKR	SPLREKRRKR	TGLRNSPLREKRRKRGLFGAIAGF
EPI_ISL_17885912	HPAI	6	KRRKR	SPLREKRRKR	TGLRNSPLREKRRKRGLFGAIAGF
EPI_ISL_17885915	HPAI	6	KRRKR	SPLREKRRKR	TGLRNSPLREKRRKRGLFGAIAGF
EPI_ISL_19765475	HPAI	6	KRRKR	SPLREKRRKR	TGLRNSPLREKRRKRGLFGAIAGF
EPI_ISL_17885918	HPAI	6	KRRKR	SPLREKRRKR	TGLRNSPLREKRRKRGLFGAIAGF
EPI_ISL_18918640	HPAI	6	KRRKR	SPLREKRRKR	TGLRNSPLREKRRKRGLFGAIAGF
EPI_ISL_17973443	HPAI	6	KRRKR	SPLREKRRKR	TGLRNSPLREKRRKRGLFGAIAGF
EPI_ISL_18698492	HPAI	6	KRRKR	SPLREKRRKR	TGLRNSPLREKRRKRGLFGAIAGF
EPI_ISL_18698494	HPAI	6	KRRKR	SPLREKRRKR	TGLRNSPLREKRRKRGLFGAIAGF
EPI_ISL_17885942	HPAI	6	KRRKR	SPLREKRRKR	TGLRNSPLREKRRKRGLFGAIAGF
EPI_ISL_18698490	HPAI	6	KRRKR	SPLREKRRKR	TGLRNSPLREKRRKRGLFGAIAGF
EPI_ISL_18698485	HPAI	6	KRRKR	SPLREKRRKR	TGLRNSPLREKRRKRGLFGAIAGF
EPI_ISL_17885944	HPAI	6	KRRKR	SPLREKRRKR	TGLRNSPLREKRRKRGLFGAIAGF
EPI_ISL_17885945	HPAI	6	KRRKR	NPLREKRRKR	TGLRNNPLREKRRKRGLFGAIAGF
EPI_ISL_18698487	HPAI	6	KRRKR	SPLREKRRKR	TGLRNSPLREKRRKRGLFGAIAGF
EPI_ISL_17885946	HPAI	6	KRRKR	SPLREKRRKR	TGLRNSPLREKRRKRGLFGAIAGF
EPI_ISL_17885948	HPAI	6	KRRKR	SPLREKRRKR	TGLRNSPLREKRRKRGLFGAIAGF
EPI_ISL_18698480	HPAI	6	KRRKR	SPLREKRRKR	TGLRNSPLREKRRKRGLFGAIAGF
EPI_ISL_17885949	HPAI	6	KRRKR	SPLREKRRKR	TGLRNSPLREKRRKRGLFGAIAGF
EPI_ISL_17885950	HPAI	6	KRRKR	SPLREKRRKR	TGLRNSPLREKRRKRGLFGAIAGF
EPI_ISL_18698482	HPAI	6	KRRKR	SPLREKRRKR	TGLRNSPLREKRRKRGLFGAIAGF
EPI_ISL_17885951	HPAI	6	KRRKR	SPLREKRRKR	TGLRNSPLREKRRKRGLFGAIAGF
EPI_ISL_18698476	HPAI	6	KRRKR	SPLREKRRKR	TGLRNSPLREKRRKRGLFGAIAGF
EPI_ISL_18698478	HPAI	6	KRRKR	SPLREKRRKR	TGLRNSPLREKRRKRGLFGAIAGF
EPI_ISL_19765465	HPAI	6	KRRKR	SPLREKRRKR	TGLRNSPLREKRRKRGLFGAIAGF
EPI_ISL_17011959	HPAI	6	KRRKR	SPLREKRRKR	TGLRNSPLREKRRKRGLFGAIAGF
EPI_ISL_17011958	HPAI	6	KRRKR	SPLREKRRKR	TGLRNSPLREKRRKRGLFGAIAGF
EPI_ISL_17885926	HPAI	6	KRRKR	SPLREKRRKR	TGLRNSPLREKRRKRGLFGAIAGF
EPI_ISL_17011957	HPAI	6	KRRKR	SPLREKRRKR	TGLRNSPLREKRRKRGLFGAIAGF
EPI_ISL_18698474	HPAI	6	KRRKR	SPLREKRRKR	TGLRNSPLREKRRKRGLFGAIAGF

EPI_ISL_17885927	HPAI	6	KRRKR	SPLREKRRKR	TGLRNSPLREKRRKRGLFGAIAGF
EPI_ISL_18698469	HPAI	6	KRRKR	SPLREKRRKR	TGLRNSPLREKRRKRGLFGAIAGF
EPI_ISL_17885928	HPAI	6	KRRKR	SPLREKRRKR	TGLRNSPLREKRRKRGLFGAIAGF
EPI_ISL_17011963	HPAI	6	KRRKR	SPLREKRRKR	TGLRNSPLREKRRKRGLFGAIAGF
EPI_ISL_17011962	HPAI	6	KRRKR	SPLREKRRKR	TGLRNSPLREKRRKRGLFGAIAGF
EPI_ISL_18698471	HPAI	6	KRRKR	SPLREKRRKR	TGLRNSPLREKRRKRGLFGAIAGF
EPI_ISL_17885930	HPAI	6	KRRKR	SPLREKRRKR	TGLRNSPLREKRRKRGLFGAIAGF
EPI_ISL_17011961	HPAI	6	KRRKR	SPLREKRRKR	TGLRNSPLREKRRKRGLFGAIAGF
EPI_ISL_17885931	HPAI	6	KRRKR	SPLREKRRKR	IGLRNSPLREKRRKRGLFGAIAGF
EPI_ISL_17885932	HPAI	6	KRRKR	SPLREKRRKR	TGLRNSPLREKRRKRGLFGAIAGF
EPI_ISL_18698464	HPAI	6	KRRKR	SPLREKRRKR	TGLRNSPLREKRRKRGLFGAIAGF
EPI_ISL_17885934	HPAI	6	KRRKR	SPLREKRRKR	TGLRNSPLREKRRKRGLFGAIAGF
EPI_ISL_18698466	HPAI	6	KRRKR	SPLREKRRKR	TGLRNSPLREKRRKRGLFGAIAGF
EPI_ISL_18265321	HPAI	6	KRRKR	SPLREKRRKR	TGLRNSPLREKRRKRGLFGAIAGF
EPI_ISL_17011964	HPAI	6	KRRKR	SPLREKRRKR	TGLRNSPLREKRRKRGLFGAIAGF
EPI_ISL_16854405	HPAI	6	RRRKR	SPLRERRRKR	TGLRNSPLRERRRKRGLFGAIAGF
EPI_ISL_19466216	HPAI	6	KRRKR	SPLREKRRKR	TGLRNSPLREKRRKRGLFGAIAGF
EPI_ISL_19466211	HPAI	6	KRRKR	SPLREKRRKR	TGLRNSPLREKRRKRGLFGAIAGF
EPI_ISL_19466213	HPAI	6	KRRKR	SPLREKRRKR	TGLRNSPLREKRRKRGLFGAIAGF
EPI_ISL_19466212	HPAI	6	KRRKR	SPLREKRRKR	TGLRNSPLREKRRKRGLFGAIAGF
EPI_ISL_19466215	HPAI	6	KRRKR	SPLREKRRKR	TGLRNSPLREKRRKRGLFGAIAGF
EPI_ISL_19466214	HPAI	6	KRRKR	SPLREKRRKR	TGLRNSPLREKRRKRGLFGAIAGF
EPI_ISL_19158339	HPAI	6	KRRKR	SPLREKRRKR	TGLRNSPLREKRRKRGLFGAIAGF
EPI_ISL_16854397	HPAI	6	RRRKR	SPLRERRRKR	TGLRNSPLRERRRKRGLFGAIAGF
EPI_ISL_18720765	HPAI	6	RRRKR	SPLRERRRKR	TGLRNSPLRERRRKRGLFGAIAGF
EPI_ISL_6959592	HPAI	6	RRRKR	SPLRERRRKR	TGLRNSPLRERRRKRGLFGAIAGF
EPI_ISL_14064697	HPAI	6	RRRKR	SPLRERRRKR	TGLRNSPLRERRRKRGLFGAIAGF
EPI_ISL_18720845	HPAI	6	RRRKR	SPLRERRRKR	TGLRNSPLRERRRKRGLFGAIAGF
EPI_ISL_20111939	HPAI	6	RRRKR	SPLRERRRKR	TGLRNSPLRERRRKRGLFGAIAGF
EPI_ISL_19624025	HPAI	6	KRRKR	SPLREKRRKR	TGLRNSPLREKRRKRGLFGAIAGF
EPI_ISL_18615599	HPAI	6	KRRKR	SPLREKRRKR	TGLRNSPLREKRRKRGLFGAIAGF
EPI_ISL_18286307	HPAI	6	RRRKR	SPLRERRRKR	TGLRNSPLRERRRKRGLFGAIAGF
EPI_ISL_20051140	HPAI	6	KRRKR	SPLREKRRKR	TGLRNSPLREKRRKRGLFGAIAGF
EPI_ISL_15647835	HPAI	6	KRRKR	SPLREKRRKR	TGLRNSPLREKRRKRGLFGAIAGF
EPI_ISL_20065029	HPAI	6	RRRKR	SPLRERRRKR	TGLRNSPLRERRRKRGLFGAIAGF
EPI_ISL_18286286	HPAI	6	RRRKR	SPLRERRRKR	TGLRNSPLRERRRKRGLFGAIAGF
EPI_ISL_20069768	HPAI	6	RRRKR	SPLRERRRKR	TGLRNSPLRERRRKRGLFGAIAGF
EPI_ISL_17950253	HPAI	6	KRRKR	SPLREKRRKR	TGLRNSPLREKRRKRGLFGAIAGF
EPI_ISL_19453461	HPAI	6	KRRKR	SPLREKRRKR	TGLRNSPLREKRRKRGLFGAIAGF
EPI_ISL_19035743	HPAI	6	KRRKR	SPLREKRRKR	TGLRNSPLREKRRKRGLFGAIAGF
EPI_ISL_19655349	HPAI	6	KRRKR	SPLREKRRKR	TGLRNSPLREKRRKRGLFGAIAGF

EPI_ISL_18720867	HPAI	6	RRRKR	SPLRERRRKR	TGLRNSPLRERRRKRGLFGAIAGF
EPI_ISL_13370925	HPAI	6	KRRKR	SPLREKRRKR	TGLRNSPLREKRRKRGLFGAIAGF
EPI_ISL_7778775	HPAI	6	RRRKR	SPLRERRRKR	TGLRNSPLRERRRKRGLFGAIAGF
EPI_ISL_18901634	HPAI	6	KRRKR	SPLREKRRKR	TGLRNSPLREKRRKRGLFGAIAGF
EPI_ISL_18901635	HPAI	6	KRRKR	SPLREKRRKR	TGLRNSPLREKRRKRGLFGAIAGF
EPI_ISL_18074199	HPAI	6	RRRKR	SPLRERRRKR	TGLRNSPLRERRRKRGLFGAIAGF
EPI_ISL_18963398	HPAI	6	KRRKR	SPLREKRRKR	TGLRNSPLREKRRKRGLFGAIAGF
EPI_ISL_18901673	HPAI	6	KRRKR	SPLREKRRKR	TGLRNSPLREKRRKRGLFGAIAGF
EPI_ISL_11561593	HPAI	6	KRRKR	SPLREKRRKR	TGLRNSPLREKRRKRGLFGAIAGF
EPI_ISL_16618967	HPAI	6	KRRKR	SPLREKRRKR	TGLRNSPLREKRRKRGLFGAIAGF
EPI_ISL_7748188	HPAI	6	KRRKR	SPLREKRRKR	TGLRNSPLREKRRKRGLFGAIAGF
EPI_ISL_17785732	HPAI	6	KRRKR	SPLREKRRKR	TGLRNSPLREKRRKRGLFGAIAGF
EPI_ISL_6029360	HPAI	6	KRRKR	SPLREKRRKR	TGLRNSPLREKRRKRGLFGAIAGF
EPI_ISL_2227276	HPAI	6	KRRKR	SPLREKRRKR	TGLRNSPLREKRRKRGLFGAIAGF
EPI_ISL_19080209	HPAI	6	KRRKR	SPLREKRRKR	TGLRNSPLREKRRKRGLFGAIAGF
EPI_ISL_18901682	HPAI	6	KRRKR	SPLREKRRKR	TGLRNSPLREKRRKRGLFGAIAGF
EPI_ISL_13370618	HPAI	6	KRRKR	SPLREKRRKR	TGLRNSPLREKRRKRGLFGAIAGF
EPI_ISL_17880904	HPAI	6	KRRKR	SPLREKRRKR	TGLRNSPLREKRRKRGLFGAIAGF
EPI_ISL_9640111	HPAI	6	KRRKR	SPLREKRRKR	TGLRNSPLREKRRKRGLFGAIAGF
EPI_ISL_18720674	HPAI	6	KRRKR	SPLREKRRKR	TGLRNSPLREKRRKRGLFGAIAGF
EPI_ISL_19775532	HPAI	6	KRRKR	SPLREKRRKR	TGLRNSPLREKRRKRGLFGAIAGF
EPI_ISL_19606632	HPAI	6	KRRKR	SPLREKRRKR	TGLRNSPLREKRRKRGLFGAIAGF
EPI_ISL_19776297	HPAI	6	KRRKR	SPLREKRRKR	TGLRNSPLREKRRKRGLFGAIAGF
EPI_ISL_19775595	HPAI	6	KRRKR	SPLREKRRKR	TGLRNSPLREKRRKRGLFGAIAGF
EPI_ISL_17964948	HPAI	6	RRRKR	SPLRERRRKR	TGLRNSPLRERRRKRGLFGAIAGF
EPI_ISL_18132957	HPAI	6	RRRKR	SPLRERRRKR	TGLRNSPLRERRRKRGLFGAIAGF
EPI_ISL_19606369	HPAI	6	RRRKR	SPLRERRRKR	TGLRNSPLRERRRKRGLFGAIAGF
EPI_ISL_19871011	HPAI	6	KRRKR	SPLREKRRKR	TGLRNSPLREKRRKRGLFGAIAGF
EPI_ISL_18132379	HPAI	6	KRRKR	SPLREKRRKR	TGLRNSPLREKRRKRGLFGAIAGF
EPI_ISL_19607353	HPAI	6	KRRKR	SPLREKRRKR	TGLRNSPLREKRRKRGLFGAIAGF
EPI_ISL_19158326	HPAI	6	KRRKR	SPLREKRRKR	TGLRNSPLREKRRKRGLFGAIAGF
EPI_ISL_19776184	HPAI	6	KRRKR	SPLREKRRKR	TGLRNSPLREKRRKRGLFGAIAGF
EPI_ISL_18132803	HPAI	6	KRRKR	SPLREKRRKR	TGLRNSPLREKRRKRGLFGAIAGF
EPI_ISL_19607986	HPAI	6	KRRKR	SPLREKRRKR	TGLRNSPLREKRRKRGLFGAIAGF
EPI_ISL_19607542	HPAI	6	KRRKR	SPLREKRRKR	TGLRNSPLREKRRKRGLFGAIAGF
EPI_ISL_18132490	HPAI	6	KRRKR	SPLREKRRKR	TGLRNSPLREKRRKRGLFGAIAGF
EPI_ISL_19775628	HPAI	6	KRRKR	SPLREKRRKR	TGLRNSPLREKRRKRGLFGAIAGF
EPI_ISL_19607562	HPAI	6	RRRKR	SPLRERRRKR	TGLRNSPLRERRRKRGLFGAIAGF
EPI_ISL_19605258	HPAI	6	KRRKR	SPLREKRRKR	TGLRNSPLREKRRKRGLFGAIAGF
EPI_ISL_17964912	HPAI	6	KRRKR	SPLREKRRKR	TGLRNSPLREKRRKRGLFGAIAGF
EPI_ISL_19776006	HPAI	6	KRRKR	SPLREKRRKR	TGLRNSPLREKRRKRGLFGAIAGF

EPI_ISL_17964857	HPAI	6	RRRKR	SPLRERRRKR	TGLRNSPLRERRRKRGLFGAIAGF
EPI_ISL_19678913	HPAI	6	KRRKR	SPLREKRRKR	TGLRNSPLREKRRKRGLFGAIAGF
EPI_ISL_19604100	HPAI	6	KRRKR	SPLREKRRKR	TGLRNSPLREKRRKRGLFGAIAGF
EPI_ISL_18737452	HPAI	6	RRRKR	SPLRERRRKR	TGLRNSPLRERRRKRGLFGAIAGF
EPI_ISL_19605209	HPAI	6	KRRKR	SPLREKRRKR	TGLRNSPLREKRRKRGLFGAIAGF
EPI_ISL_20082756	HPAI	6	KRRKR	SPLREKRRKR	TGLRNSPLREKRRKRGLFGAIAGF
EPI_ISL_16190849	HPAI	6	KRRKR	SPLREKRRKR	TGLRNSPLREKRRKRGLFGAIAGF
EPI_ISL_19592595	HPAI	6	KRRKR	SPLREKRRKR	TGLRNSPLREKRRKRGLFGAIAGF
EPI_ISL_19154653	HPAI	6	KRRKR	SPLREKRRKR	TGLRNSPLREKRRKRGLFGAIAGF
EPI_ISL_19604369	HPAI	6	RRRKR	SPLRERRRKR	TGLRNSPLRERRRKRGLFGAIAGF
EPI_ISL_19560337	HPAI	7	KRRKR	SRLREKRRKR	TGLRNSRLREKRRKRGLFGAIAGF
EPI_ISL_19158296	HPAI	6	RRRKR	SPLRERRRKR	TGLRNSPLRERRRKRGLFGAIAGF
EPI_ISL_18133416	HPAI	6	KRRKR	SPLREKRRKR	TGLRNSPLREKRRKRGLFGAIAGF
EPI_ISL_19303641	HPAI	6	RRRKR	SPLRERRRKR	TGLRNSPLRERRRKRGLFGAIAGF

Supplementary Table S4. Temporal dynamics and inferred ancestral origins of avian influenza A viruses based on the HA segment.

Inferred ancestral	Country of origin	Chronological order	Tip date	Estimated	Height 95%	Height 95%	Host	Host calssification	ID (EPI_ISL)
USA	Colombia	1st	2022,77	2022,57	2022,66	2022,44	Duck sp.	Domestic	17353839
USA	Colombia	2nd	2022,85	2022,74	2022,84	2022,62	Chicken sp.	Domestic	17353507
USA	Peru	3rd	2022,86	2022,58	2022,68	2022,47	Pelican (*Pelecanus thagus*)	Wild bird	17806000
USA	Venezuela	4th	2022,90	2022,60	2022,71	2022,44	Pelican (*Pelecanus occidentalis*)	Wild bird	16854405
Peru	Chile	5th	2022,92	2022,65	2022,73	2022,56	Black skimmer (*Rynchops niger*)	Wild bird	16891402
Peru	Ecuador	6th	2023,03	2022,93	2023,02	2022,83	Magnificent frigatebird (*Fregata magnificens*)	Wild bird	17973443
Argentina	Bolivia	7th	2023,07	2022,92	2022,99	2022,82	Chicken sp.	Domestic	19410267
USA	Argentina	8th	2023,11	2022,58	2022,68	2022,47	Goose (*Andean Guayata*)	Wild bird	18698459
Argentina	Uruguay	9th	2023,13	2023,09	2023,13	2023,02	Black-necked swan (Cygnus melancoryphus)	Wild bird	18310958
Argentina	Brazil	10th	2023,40	2023,13	2023,20	2023,04	Common tern (*Sterna hirundo*)	Wild bird	19215183
Argentina	Antartctica	11th	2023,77	2023,64	2023,73	2023,51	Brown Skua (*Stercorarius antarcticus*)	Wild bird	18439563
Argentina	Falkland Islands	12th	2023,83	2023,73	2023,82	2023,56	Southern fulmar (*Fulmarus glacialoides*)	Wild bird	18522961

Supplementary Table S5. Temporal dynamics and inferred ancestral origins of avian influenza A viruses based on the NA segment.

Inferred ancestral	Country of origin	Chronological order	Tip date	Estimated	Height 95%	Height 95%	Host	Host	ID (EPI_ISL)
USA	Colombia	1st	2022,83	2022,43	2022,57	2022,27	Duck sp.	Domestic	17353509
USA	Colombia	2nd	2022,85	2021,92	2022,20	2021,61	Chicken sp.	Domestic	17353507
USA	Peru	3rd	2022,86	2022,46	2022,62	2022,26	Owl sp.	Wild bird	17660073
USA	Venezuela	4th	2022,90	2022,57	2022,71	2022,40	Pelican (*Pelecanus occidentalis*)	Wild bird	16013752
USA	Chile	5th	2022,92	2022,52	2022,66	2022,39	Black skimmer (*Rynchops niger*)	Wild bird	16891402
Peru	Ecuador	6th	2023,03	2022,88	2023,00	2022,76	Magnificent frigatebird (*Fregata magnificens*)	Wild bird	17973443
Chile	Bolivia	7th	2023,14	2022,91	2022,95	2022,70	Chicken sp.	Domestic	19410267
Chile	Argentina	8th	2023,19	2022,91	2022,95	2022,70	Goose (*Andean Guayata*)	Wild bird	18698459
Argentina	Uruguay	9th	2023,21	2023,06	2023,07	2022,91	Black-necked swan (Cygnus melancoryphus)	Wild bird	18310958
Argentina	Brazil	10th	2023,50	2023,18	2023,17	2023,03	Royal tern (*Thalasseus maximus*)	Wild bird	19215189
Brazil	Falkland Islands	11th	2023,83	2023,43	2023,57	2023,26	Southern fulmar (*Fulmarus glacialoides*)	Wild bird	18522961
Argentina	Antarctica	12th	2023,84	2023,18	2023,22	2023,00	Brown Skua (*Stercorarius antarcticus*)	Wild bird	18439562

Supplementary Table S6. Complete sequence metadata uses for phylogenetic analysis

Virus name	PB2	PB1	PA	NP	MP	HA	NA	NS	Country
A/American_Black_Duck/NL/OTH-0041-118/2023	EPI_ISL_19775595	EPI_ISL_19775595	EPI_ISL_19775595	EPI_ISL_19775595		EPI_ISL_19775595	EPI_ISL_19775595	EPI_ISL_19775595	USA
A/american_crow/Massachusetts/22-008951-007/2022	EPI_ISL_19606632	EPI_ISL_19606632	EPI_ISL_19606632	EPI_ISL_19606632	EPI_ISL_19606632	EPI_ISL_19606632	EPI_ISL_19606632	EPI_ISL_19606632	USA
A/American_Crow/NS/FAV-0181-2/2024	EPI_ISL_19776297	EPI_ISL_19776297	EPI_ISL_19776297	EPI_ISL_19776297	EPI_ISL_19776297	EPI_ISL_19776297	EPI_ISL_19776297	EPI_ISL_19776297	USA
A/american_green_winged_teal/Idaho/22-034533-007-R2/2022	EPI_ISL_19606709	EPI_ISL_19606709	EPI_ISL_19606709	EPI_ISL_19606709	EPI_ISL_19606709	EPI_ISL_19606709	EPI_ISL_19606709	EPI_ISL_19606709	USA
A/antarctic_fur_seal/Antarctica/O2904-BR/2024	EPI_ISL_20180699	EPI_ISL_20180699	EPI_ISL_20180699	EPI_ISL_20180699	EPI_ISL_20180699	EPI_ISL_20180699	EPI_ISL_20180699	EPI_ISL_20180699	Antarctica
A/antarctic_fur_seal/Antarctica/O2913-BR/2024	EPI_ISL_20180700	EPI_ISL_20180700	EPI_ISL_20180700	EPI_ISL_20180700	EPI_ISL_20180700	EPI_ISL_20180700	EPI_ISL_20180700	EPI_ISL_20180700	Antarctica
A/antarctic_fur_seal/Antarctica/O3122-NS/2024	EPI_ISL_20180697	EPI_ISL_20180697	EPI_ISL_20180697	EPI_ISL_20180697	EPI_ISL_20180697	EPI_ISL_20180697	EPI_ISL_20180697	EPI_ISL_20180697	Antarctica
A/antarctic_fur_seal/Antarctica/O3122-OR/2024	EPI_ISL_20180698								Antarctica
A/antarctic_fur_seal/Antarctica/O3122-BR/2024			EPI_ISL_19879315	EPI_ISL_19879315		EPI_ISL_19879315			Antarctica
A/antarctic shag/Antarctica/O3348-OR/2025		EPI_ISL_20182795							Antarctica
A/Antarctic_Fur_Seal/Jason_Harbour/141037/2023	EPI_ISL_18742212					EPI_ISL_18742212	EPI_ISL_18742212		Antarctica
A/Antofagasta/25945/2023	EPI_ISL_19391463		EPI_ISL_19391463				EPI_ISL_19391463	EPI_ISL_19391463	Chile
A/antarctic_shag/Antarctica/O3348-OR/2025				EPI_ISL_20182795	EPI_ISL_20182795	EPI_ISL_20182795			Antarctica
A/Antarctic_tern/King_Edward_Point/141271/2023						EPI_ISL_18742220			Antarctica
A/Avian/Argentina/1762-2/2023	EPI_ISL_18698516	EPI_ISL_18698516	EPI_ISL_18698516	EPI_ISL_18698516	EPI_ISL_18698516	EPI_ISL_18698516	EPI_ISL_18698516	EPI_ISL_18698516	Argentina
A/Avian/Argentina/1790-5/2023	EPI_ISL_18698517	EPI_ISL_18698517	EPI_ISL_18698517	EPI_ISL_18698517	EPI_ISL_18698517	EPI_ISL_18698517	EPI_ISL_18698517	EPI_ISL_18698517	Argentina
A/Avian/Argentina/579-5/2023	EPI_ISL_18698485	EPI_ISL_18698485	EPI_ISL_18698485	EPI_ISL_18698485	EPI_ISL_18698485		EPI_ISL_18698485		Argentina
A/Avian/Argentina/586-4/2023	EPI_ISL_18698487	EPI_ISL_18698487	EPI_ISL_18698487	EPI_ISL_18698487	EPI_ISL_18698487	EPI_ISL_18698487			Argentina
A/avian/Peru/AISA0446/2022	EPI_ISL_17805997	EPI_ISL_17805997					EPI_ISL_17805997		Peru
A/avian/Peru/AISA0451/2022			EPI_ISL_17805998					EPI_ISL_17805998	Peru
A/backyard_chicken/Uruguay/UDELAR-040-M7/2023			EPI_ISL_18310967	EPI_ISL_18310967	EPI_ISL_18310967			EPI_ISL_18310967	Uruguay
A/backyard_chicken/Uruguay/UDELAR-040-M5/2023		EPI_ISL_18310942			EPI_ISL_18310942				Uruguay
A/backyard_chicken/Uruguay/UDELAR-047-M3/2023	EPI_ISL_18310965	EPI_ISL_18310965	EPI_ISL_18310965		EPI_ISL_18310965	EPI_ISL_18310965	EPI_ISL_18310965	EPI_ISL_18310965	Uruguay
A/backyard_chicken/Uruguay/UDELAR-127-M1/2023			EPI_ISL_18310963						Uruguay
A/backyard chicken/Uruguay/UDELAR-047-M1/2023								EPI_ISL_18310966	Uruguay
A/backyard_chicken/Uruguay/UDELAR-127-M4/2023	EPI_ISL_18310962	EPI_ISL_18310962	EPI_ISL_18310962	EPI_ISL_18310962		EPI_ISL_18310962	EPI_ISL_18310962	EPI_ISL_18310962	Uruguay
A/backyard_chicken/Uruguay/UDELAR-144-M3/2023	EPI_ISL_18310961	EPI_ISL_18310961	EPI_ISL_18310961	EPI_ISL_18310961		EPI_ISL_18310961	EPI_ISL_18310961	EPI_ISL_18310961	Uruguay
A/backyard_turkey/Uruguay/UDELAR-124-M6/2023	EPI_ISL_18310959	EPI_ISL_18310959	EPI_ISL_18310959	EPI_ISL_18310959			EPI_ISL_18310959	EPI_ISL_18310959	Uruguay
A/Bald_Eagle/Alaska/USDA-013001-001/2022	EPI_ISL_18132957	EPI_ISL_18132957	EPI_ISL_18132957	EPI_ISL_18132957		EPI_ISL_18132957	EPI_ISL_18132957	EPI_ISL_18132957	USA
A/bald_eagle/Virginia/23-009210-001/2023	EPI_ISL_17964912	EPI_ISL_17964912	EPI_ISL_17964912	EPI_ISL_17964912	EPI_ISL_17964912	EPI_ISL_17964912		EPI_ISL_17964912	USA
A/Band-tailed_gull/Antofagasta/228525-1/2022		EPI_ISL_19131166		EPI_ISL_19131166	EPI_ISL_19131166	EPI_ISL_19131166	EPI_ISL_19131166		Chile
A/Band-tailed_gull/Arica_y_Parinacota/232200-1/2023	EPI_ISL_19131168	EPI_ISL_19131168		EPI_ISL_19131168	EPI_ISL_19131168	EPI_ISL_19131168	EPI_ISL_19131168	EPI_ISL_19131168	Chile
A/barnacle_goose/Finland/6955_21VIR7689-9/2021	EPI_ISL_7778775	EPI_ISL_7778775	EPI_ISL_7778775	EPI_ISL_7778775	EPI_ISL_7778775	EPI_ISL_7778775	EPI_ISL_7778775	EPI_ISL_7778775	Eurasia
A/barnacle_goose/Netherlands/21027357-002/2021	EPI_ISL_2227276	EPI_ISL_2227276	EPI_ISL_2227276	EPI_ISL_2227276	EPI_ISL_2227276	EPI_ISL_2227276	EPI_ISL_2227276	EPI_ISL_2227276	Eurasia
A/Belcher_gull/Peru/UNMSM-A267/2022	EPI_ISL_18371664							EPI_ISL_18371664	Peru
A/black_skimmer/Chile/C61962/2022	EPI_ISL_16891402	EPI_ISL_16891402	EPI_ISL_16891402	EPI_ISL_16891402	EPI_ISL_16891402	EPI_ISL_16891402	EPI_ISL_16891402	EPI_ISL_16891402	Chile
A/Black_Skimmer/Maule/240379/2023	EPI_ISL_17885849	EPI_ISL_17885849	EPI_ISL_17885849	EPI_ISL_17885849			EPI_ISL_17885849		Chile
A/black_vulture/Alabama/23-040176-001-original/2023	EPI_ISL_19560337	EPI_ISL_19560337	EPI_ISL_19560337	EPI_ISL_19560337	EPI_ISL_19560337	EPI_ISL_19560337	EPI_ISL_19560337	EPI_ISL_19560337	USA
A/black_vulture/Alabama/23-040176-002-original/2023	EPI_ISL_19560339	EPI_ISL_19560339	EPI_ISL_19560339	EPI_ISL_19560339	EPI_ISL_19560339	EPI_ISL_19560339	EPI_ISL_19560339	EPI_ISL_19560339	USA
A/black_vulture/Mississippi/23-001343-002/2023	EPI_ISL_19606295	EPI_ISL_19606295	EPI_ISL_19606295	EPI_ISL_19606295	EPI_ISL_19606295	EPI_ISL_19606295	EPI_ISL_19606295	EPI_ISL_19606295	USA
A/black_vulture/Mississippi/23-001343-015/2023	EPI_ISL_19606308						EPI_ISL_19606308		USA
A/black-headed_gull/England/388256/2022	EPI_ISL_11561593	EPI_ISL_11561593	EPI_ISL_11561593	EPI_ISL_11561593	EPI_ISL_11561593	EPI_ISL_11561593	EPI_ISL_11561593	EPI_ISL_11561593	Eurasia
A/Black-Necked_Swan/Neuquen/1151-1/2023	EPI_ISL_19836182		EPI_ISL_19836182	EPI_ISL_19836182		EPI_ISL_19836182	EPI_ISL_19836182		Argentina
A/black-necked_swan/Uruguay/UDELAR-014-M3/2023				EPI_ISL_18310958	EPI_ISL_18310958	EPI_ISL_18310958	EPI_ISL_18310958	EPI_ISL_18310958	Uruguay
A/black-necked_swan/Uruguay/UDELAR-078-M2/2023	EPI_ISL_18310957	EPI_ISL_18310957	EPI_ISL_18310957	EPI_ISL_18310957	EPI_ISL_18310957	EPI_ISL_18310957	EPI_ISL_18310957	EPI_ISL_18310957	Uruguay
A/Blackish_oystercatcher/OHiggins/240628/2023	EPI_ISL_17885967	EPI_ISL_17885967		EPI_ISL_17885967	EPI_ISL_17885967	EPI_ISL_17885967	EPI_ISL_17885967		Chile
A/blue_winged_teal/Oklahoma/22-029332-029/2022	EPI_ISL_19606369	EPI_ISL_19606369	EPI_ISL_19606369	EPI_ISL_19606369	EPI_ISL_19606369	EPI_ISL_19606369	EPI_ISL_19606369	EPI_ISL_19606369	USA
A/Blue-winged_Teal/South_Carolina/USDA-000345-003/2021	EPI_ISL_18133416	EPI_ISL_18133416	EPI_ISL_18133416	EPI_ISL_18133416		EPI_ISL_18133416	EPI_ISL_18133416	EPI_ISL_18133416	USA
A/brown_booby/Peru/23-005629-003/2022	EPI_ISL_17660074	EPI_ISL_17660074	EPI_ISL_17660074		EPI_ISL_17660074	EPI_ISL_17660074		EPI_ISL_17660074	Peru
A/Brown_skua/Bird_Island/128287/2023		EPI_ISL_18439562			EPI_ISL_18439562	EPI_ISL_18439562	EPI_ISL_18439562		Antarctica
A/Brown_skua/Bird_Island/128288/2023	EPI_ISL_18439563				EPI_ISL_18439563	EPI_ISL_18439563			Antarctica
A/Brown_skua/Bird_Island/128289/2023	EPI_ISL_18439564	EPI_ISL_18439564	EPI_ISL_18439564	EPI_ISL_18439564			EPI_ISL_18439564	EPI_ISL_18439564	Antarctica
A/Brown_Skua/Bird_Island/141232/2023			EPI_ISL_18742214			EPI_ISL_18742214			Antarctica
A/brown_skua/Crozet/24P023412/2024						EPI_ISL_19747215	EPI_ISL_19747215	EPI_ISL_19747215	Antarctica
A/Brown_skua/Fildes_Peninsula/B1/2024_H5N1_				EPI_ISL_19847535	EPI_ISL_19847535			EPI_ISL_19847535	Antarctica
A/Brown_skua/Fildes_Peninsula/B2/2024_H5N1_	EPI_ISL_19847536	EPI_ISL_19847536	EPI_ISL_19847536			EPI_ISL_19847536			Antarctica
A/Brown_skua/Fildes_Peninsula/F4/2024_H5N1_	EPI_ISL_19847539								Antarctica
A/Brown_Skua/Gough_Island/047354/2024	EPI_ISL_20151596	EPI_ISL_20151596	EPI_ISL_20151596	EPI_ISL_20151596	EPI_ISL_20151596	EPI_ISL_20151596	EPI_ISL_20151596	EPI_ISL_20151596	Antarctica
A/Brown_Skua/Gough_Island/047355/2024	EPI_ISL_20151597				EPI_ISL_20151597				Antarctica
A/Brown_skua/Hope_Bay/SK02-30/2024	EPI_ISL_20179748	EPI_ISL_20179748	EPI_ISL_20179748	EPI_ISL_20179748	EPI_ISL_20179748	EPI_ISL_20179748	EPI_ISL_20179748	EPI_ISL_20179748	Antarctica
A/Brown_Skua/Hound_Bay/133947/2023	EPI_ISL_18592428	EPI_ISL_18592428	EPI_ISL_18592428	EPI_ISL_18592428	EPI_ISL_18592428	EPI_ISL_18592428			Antarctica
A/Brown_Skua/Hound_Bay/133949/2023	EPI_ISL_18592429	EPI_ISL_18592429	EPI_ISL_18592429		EPI_ISL_18592429		EPI_ISL_18592429		Antarctica
A/brown_skua/Kerguelen/24P023443/2024				EPI_ISL_19747221	EPI_ISL_19747221	EPI_ISL_19747221			Antarctica
A/brown_skua/Kerguelen/24P023446/2024			EPI_ISL_19747220						Antarctica
A/Brown_Skua/Moltke_Harbour/133752/2023	EPI_ISL_18592424		EPI_ISL_18592424	EPI_ISL_18592424		EPI_ISL_18592424	EPI_ISL_18592424	EPI_ISL_18592424	Antarctica
A/Brown_Skua/Moltke_Harbour/133755/2023	EPI_ISL_18592426		EPI_ISL_18592426			EPI_ISL_18592426	EPI_ISL_18592426		Antarctica
A/Brown_Skua/Penguin_River/141236/2023					EPI_ISL_18742216	EPI_ISL_18742216			Antarctica
A/Brown_Skua/Penguin_River/141240/2023	EPI_ISL_18742218	EPI_ISL_18742218	EPI_ISL_18742218	EPI_ISL_18742218		EPI_ISL_18742218	EPI_ISL_18742218	EPI_ISL_18742218	Antarctica
A/Brown_skua/Torgersen_Island/o8182/2024	EPI_ISL_19745586	EPI_ISL_19745586	EPI_ISL_19745586	EPI_ISL_19745586	EPI_ISL_19745586	EPI_ISL_19745586			Antarctica
A/buff-necked_ibis/Bio_bio/247636-1/2023	EPI_ISL_19418491		EPI_ISL_19418491	EPI_ISL_19418491	EPI_ISL_19418491	EPI_ISL_19418491	EPI_ISL_19418491	EPI_ISL_19418491	Chile
A/burmeisters_porpoise/Chile/246506-2/2023		EPI_ISL_19391458	EPI_ISL_19391458	EPI_ISL_19391458	EPI_ISL_19391458	EPI_ISL_19391458			Chile
A/Buteogallus_urubitinga/Espirito_Santo/1630-SC/2023	EPI_ISL_19215175	EPI_ISL_19215175	EPI_ISL_19215175	EPI_ISL_19215175			EPI_ISL_19215175	EPI_ISL_19215175	Brazil
A/Cackling_Goose/BC/AIVPHL-1690/2024	EPI_ISL_19303641	EPI_ISL_19303641	EPI_ISL_19303641	EPI_ISL_19303641	EPI_ISL_19303641	EPI_ISL_19303641	EPI_ISL_19303641	EPI_ISL_19303641	USA
A/Calidris_alba/Lima/NA_6/2023							EPI_ISL_19158337		Peru
A/Calidris_alba/Lima/PB2_1/2023	EPI_ISL_19158333								Peru
A/Calidris_alba/Lima/PA_3/2023			EPI_ISL_19158344						Peru
A/Calidris_alba/Lima/NP_5/2023				EPI_ISL_19158335					Peru
A/Calidris_alba/Lima/HA_4/2023						EPI_ISL_19158339			Peru
A/Calidris_alba/Lima/NEP_8/2023								EPI_ISL_19158336	Peru
A/Canada_Goose/AB/FAV-0026-14/2023	EPI_ISL_19775532	EPI_ISL_19775532	EPI_ISL_19775532	EPI_ISL_19775532	EPI_ISL_19775532	EPI_ISL_19775532	EPI_ISL_19775532	EPI_ISL_19775532	USA
A/Canada_Goose/NB/FAV-0036-1/2024	EPI_ISL_19776006	EPI_ISL_19776006	EPI_ISL_19776006	EPI_ISL_19776006	EPI_ISL_19776006	EPI_ISL_19776006	EPI_ISL_19776006	EPI_ISL_19776006	USA
A/Canada_Goose/ON/FAV-0118-9/2023	EPI_ISL_19775581	EPI_ISL_19775581	EPI_ISL_19775581	EPI_ISL_19775581	EPI_ISL_19775581	EPI_ISL_19775581	EPI_ISL_19775581	EPI_ISL_19775581	USA
A/Canada_Goose/PE/FAV-0035-1/2024	EPI_ISL_19776184	EPI_ISL_19776184	EPI_ISL_19776184	EPI_ISL_19776184	EPI_ISL_19776184	EPI_ISL_19776184	EPI_ISL_19776184	EPI_ISL_19776184	USA
A/cape_petrel/Crozet/24P023427/2024						EPI_ISL_19747216			Antarctica
A/caspian_tern/Washington/23-025001-001/2023	EPI_ISL_18737454	EPI_ISL_18737454	EPI_ISL_18737454	EPI_ISL_18737454		EPI_ISL_18737454	EPI_ISL_18737454		USA
A/chicken/Araucania/239189-2/2023	EPI_ISL_17885958	EPI_ISL_17885958	EPI_ISL_17885958				EPI_ISL_17885958	EPI_ISL_17885958	Chile
A/chicken/Araucania/239569-1/2023		EPI_ISL_17885954				EPI_ISL_17885954	EPI_ISL_17885954		Chile
A/chicken/Araucania/239569-2/2023				EPI_ISL_17885952		EPI_ISL_17885952	EPI_ISL_17885952		Chile
A/chicken/Araucania/240481-1/2023	EPI_ISL_17885949		EPI_ISL_17885949	EPI_ISL_17885949	EPI_ISL_17885949	EPI_ISL_17885949	EPI_ISL_17885949		Chile
A/chicken/Araucania/241892-2/2023			EPI_ISL_17885946	EPI_ISL_17885946	EPI_ISL_17885946	EPI_ISL_17885946	EPI_ISL_17885946	EPI_ISL_17885946	Chile
A/chicken/Araucania/241914-1/2023		EPI_ISL_17885945	EPI_ISL_17885945	EPI_ISL_17885945		EPI_ISL_17885945	EPI_ISL_17885945		Chile
A/chicken/Araucania/244469/2023	EPI_ISL_18760073	EPI_ISL_18760073	EPI_ISL_18760073	EPI_ISL_18760073		EPI_ISL_18760073	EPI_ISL_18760073	EPI_ISL_18760073	Chile
A/Chicken/Argentina/1035-1/2023	EPI_ISL_18698504		EPI_ISL_18698504	EPI_ISL_18698504		EPI_ISL_18698504	EPI_ISL_18698504	EPI_ISL_18698504	Chile
A/Chicken/Argentina/1147-2/2023		EPI_ISL_18698505		EPI_ISL_18698505	EPI_ISL_18698505	EPI_ISL_18698505	EPI_ISL_18698505		Argentina
A/Chicken/Argentina/1200-1/2023	EPI_ISL_18698506	EPI_ISL_18698506	EPI_ISL_18698506	EPI_ISL_18698506	EPI_ISL_18698506	EPI_ISL_18698506	EPI_ISL_18698506	EPI_ISL_18698506	Argentina
A/Chicken/Argentina/1340-2/2023	EPI_ISL_18698507	EPI_ISL_18698507	EPI_ISL_18698507	EPI_ISL_18698507	EPI_ISL_18698507	EPI_ISL_18698507	EPI_ISL_18698507	EPI_ISL_18698507	Argentina
A/Chicken/Argentina/1416-3/2023	EPI_ISL_18698510	EPI_ISL_18698510	EPI_ISL_18698510			EPI_ISL_18698510	EPI_ISL_18698510	EPI_ISL_18698510	Argentina
A/Chicken/Argentina/1530-3/2023	EPI_ISL_18698511	EPI_ISL_18698511	EPI_ISL_18698511	EPI_ISL_18698511		EPI_ISL_18698511	EPI_ISL_18698511	EPI_ISL_18698511	Argentina
A/Chicken/Argentina/1708-1/2023	EPI_ISL_18698512	EPI_ISL_18698512	EPI_ISL_18698512	EPI_ISL_18698512	EPI_ISL_18698512	EPI_ISL_18698512	EPI_ISL_18698512	EPI_ISL_18698512	Argentina
A/Chicken/Argentina/1976-2/2023	EPI_ISL_18698518	EPI_ISL_18698518	EPI_ISL_18698518	EPI_ISL_18698518		EPI_ISL_18698518	EPI_ISL_18698518		Argentina
A/Chicken/Argentina/1984-4/2023	EPI_ISL_18698519	EPI_ISL_18698519	EPI_ISL_18698519	EPI_ISL_18698519	EPI_ISL_18698519	EPI_ISL_18698519	EPI_ISL_18698519	EPI_ISL_18698519	Argentina
A/Chicken/Argentina/2016-2/2023	EPI_ISL_18698732	EPI_ISL_18698732	EPI_ISL_18698732	EPI_ISL_18698732	EPI_ISL_18698732	EPI_ISL_18698732	EPI_ISL_18698732	EPI_ISL_18698732	Argentina
A/Chicken/Argentina/2034-5/2023	EPI_ISL_18698520	EPI_ISL_18698520	EPI_ISL_18698520	EPI_ISL_18698520	EPI_ISL_18698520	EPI_ISL_18698520		EPI_ISL_18698520	Argentina
A/Chicken/Argentina/2049-3/2023	EPI_ISL_18698521	EPI_ISL_18698521	EPI_ISL_18698521			EPI_ISL_18698521	EPI_ISL_18698521	EPI_ISL_18698521	Argentina
A/Chicken/Argentina/2305-1/2023			EPI_ISL_18698524			EPI_ISL_18698524	EPI_ISL_18698524		Argentina
A/Chicken/Argentina/2305-6/2023			EPI_ISL_18698525	EPI_ISL_18698525				EPI_ISL_18698525	Argentina
A/Chicken/Argentina/2483-3/2023	EPI_ISL_18698526	EPI_ISL_18698526	EPI_ISL_18698526	EPI_ISL_18698526		EPI_ISL_18698526	EPI_ISL_18698526		Argentina
A/Chicken/Argentina/2796-2/2023	EPI_ISL_18698527	EPI_ISL_18698527	EPI_ISL_18698527	EPI_ISL_18698527	EPI_ISL_18698527	EPI_ISL_18698527	EPI_ISL_18698527	EPI_ISL_18698527	Argentina
A/Chicken/Argentina/3346-1/2023	EPI_ISL_18698528	EPI_ISL_18698528	EPI_ISL_18698528	EPI_ISL_18698528	EPI_ISL_18698528	EPI_ISL_18698528	EPI_ISL_18698528	EPI_ISL_18698528	Argentina
A/Chicken/Argentina/3657-2/2023	EPI_ISL_18698529	EPI_ISL_18698529	EPI_ISL_18698529	EPI_ISL_18698529	EPI_ISL_18698529	EPI_ISL_18698529	EPI_ISL_18698529	EPI_ISL_18698529	Argentina
A/Chicken/Argentina/3695-3/2023	EPI_ISL_18698530	EPI_ISL_18698530	EPI_ISL_18698530	EPI_ISL_18698530		EPI_ISL_18698530	EPI_ISL_18698530		Argentina
A/Chicken/Argentina/464-4/2023	EPI_ISL_18698460	EPI_ISL_18698460	EPI_ISL_18698460	EPI_ISL_18698460	EPI_ISL_18698460	EPI_ISL_18698460	EPI_ISL_18698460	EPI_ISL_18698460	Argentina
A/Chicken/Argentina/477-5/2023	EPI_ISL_18698462	EPI_ISL_18698462	EPI_ISL_18698462		EPI_ISL_18698462	EPI_ISL_18698462			Argentina
A/Chicken/Argentina/481-2/2023	EPI_ISL_18698464	EPI_ISL_18698464	EPI_ISL_18698464	EPI_ISL_18698464	EPI_ISL_18698464	EPI_ISL_18698464	EPI_ISL_18698464	EPI_ISL_18698464	Argentina
A/Chicken/Argentina/485-6/2023	EPI_ISL_18698466	EPI_ISL_18698466	EPI_ISL_18698466	EPI_ISL_18698466	EPI_ISL_18698466	EPI_ISL_18698466	EPI_ISL_18698466		Argentina
A/Chicken/Argentina/491-2/2023	EPI_ISL_18698469	EPI_ISL_18698469	EPI_ISL_18698469	EPI_ISL_18698469	EPI_ISL_18698469	EPI_ISL_18698469	EPI_ISL_18698469		Argentina
A/Chicken/Argentina/501-1/2023	EPI_ISL_18698471	EPI_ISL_18698471	EPI_ISL_18698471	EPI_ISL_18698471	EPI_ISL_18698471	EPI_ISL_18698471	EPI_ISL_18698471		Argentina
A/Chicken/Argentina/506-2/2023	EPI_ISL_18698474	EPI_ISL_18698474	EPI_ISL_18698474	EPI_ISL_18698474	EPI_ISL_18698474	EPI_ISL_18698474	EPI_ISL_18698474		Argentina
A/Chicken/Argentina/509-2/2023	EPI_ISL_18698476	EPI_ISL_18698476	EPI_ISL_18698476		EPI_ISL_18698476	EPI_ISL_18698476	EPI_ISL_18698476	EPI_ISL_18698476	Argentina
A/Chicken/Argentina/516-5/2025		EPI_ISL_19823064		EPI_ISL_19823064	EPI_ISL_19823064	EPI_ISL_19823064	EPI_ISL_19823064		Argentina
A/Chicken/Argentina/516-6/2025						EPI_ISL_19823067			Argentina
A/Chicken/Argentina/556-6/2023	EPI_ISL_18698478	EPI_ISL_18698478	EPI_ISL_18698478	EPI_ISL_18698478	EPI_ISL_18698478	EPI_ISL_18698478			Argentina
A/Chicken/Argentina/559-8/2023	EPI_ISL_18698480		EPI_ISL_18698480	EPI_ISL_18698480	EPI_ISL_18698480	EPI_ISL_18698480	EPI_ISL_18698480		Argentina
A/Chicken/Argentina/578-2/2023	EPI_ISL_18698482	EPI_ISL_18698482	EPI_ISL_18698482	EPI_ISL_18698482		EPI_ISL_18698482	EPI_ISL_18698482	EPI_ISL_18698482	Argentina
A/Chicken/Argentina/588-4/2023	EPI_ISL_18698490	EPI_ISL_18698490	EPI_ISL_18698490	EPI_ISL_18698490		EPI_ISL_18698490	EPI_ISL_18698490	EPI_ISL_18698490	Argentina
A/Chicken/Argentina/606-1/2023	EPI_ISL_18698492	EPI_ISL_18698492		EPI_ISL_18698492		EPI_ISL_18698492			Argentina
A/Chicken/Argentina/736-1/2023	EPI_ISL_18698494	EPI_ISL_18698494	EPI_ISL_18698494		EPI_ISL_18698494	EPI_ISL_18698494	EPI_ISL_18698494		Argentina
A/Chicken/Argentina/747-1/2023	EPI_ISL_18698730		EPI_ISL_18698730	EPI_ISL_18698730		EPI_ISL_18698730			Argentina
A/Chicken/Argentina/895-1/2023	EPI_ISL_18698731	EPI_ISL_18698731	EPI_ISL_18698731	EPI_ISL_18698731	EPI_ISL_18698731	EPI_ISL_18698731	EPI_ISL_18698731	EPI_ISL_18698731	Argentina
A/Chicken/Argentina/919-3/2023	EPI_ISL_18698502	EPI_ISL_18698502	EPI_ISL_18698502	EPI_ISL_18698502		EPI_ISL_18698502	EPI_ISL_18698502		Argentina
A/chicken/Atacama/235254-2/2023	EPI_ISL_18760074	EPI_ISL_18760074	EPI_ISL_18760074	EPI_ISL_18760074		EPI_ISL_18760074	EPI_ISL_18760074		Chile
A/chicken/Aysen/250755-2/2023	EPI_ISL_19404916	EPI_ISL_19404916	EPI_ISL_19404916	EPI_ISL_19404916		EPI_ISL_19404916	EPI_ISL_19404916		Chile
A/chicken/Bolivar/ICA-3500/2022	EPI_ISL_17353507	EPI_ISL_17353507	EPI_ISL_17353507	EPI_ISL_17353507	EPI_ISL_17353507	EPI_ISL_17353507	EPI_ISL_17353507	EPI_ISL_17353507	Venezuela
A/chicken/Chaco/516-4/2025		EPI_ISL_19752381		EPI_ISL_19752381	EPI_ISL_19752381	EPI_ISL_19752381	EPI_ISL_19752381		Argentina
A/chicken/Chelyabinsk_region/2KS/Russia/2021	EPI_ISL_18720674	EPI_ISL_18720674	EPI_ISL_18720674	EPI_ISL_18720674	EPI_ISL_18720674	EPI_ISL_18720674	EPI_ISL_18720674	EPI_ISL_18720674	Antarctica
A/chicken/Chiba/21B4T/2022	EPI_ISL_18720765	EPI_ISL_18720765	EPI_ISL_18720765	EPI_ISL_18720765	EPI_ISL_18720765	EPI_ISL_18720765	EPI_ISL_18720765	EPI_ISL_18720765	Eurasia
A/chicken/Choco/ICA-3502/2022						EPI_ISL_17353508	EPI_ISL_17353508		Colombia
A/chicken/Choco/ICA-3504/2022	EPI_ISL_17353509	EPI_ISL_17353509	EPI_ISL_17353509			EPI_ISL_17353509	EPI_ISL_17353509	EPI_ISL_17353509	Colombia
A/chicken/Cochabamba/39356/2023	EPI_ISL_19410267	EPI_ISL_19410267	EPI_ISL_19410267	EPI_ISL_19410267	EPI_ISL_19410267	EPI_ISL_19410267	EPI_ISL_19410267	EPI_ISL_19410267	Bolivia
A/chicken/Cochabamba/39383/2023	EPI_ISL_19410268	EPI_ISL_19410268	EPI_ISL_19410268	EPI_ISL_19410268	EPI_ISL_19410268	EPI_ISL_19410268	EPI_ISL_19410268	EPI_ISL_19410268	Bolivia
A/chicken/Cochabamba/39425/2023	EPI_ISL_19410269	EPI_ISL_19410269	EPI_ISL_19410269	EPI_ISL_19410269	EPI_ISL_19410269	EPI_ISL_19410269	EPI_ISL_19410269	EPI_ISL_19410269	Bolivia
A/chicken/Cochabamba/39476/2023	EPI_ISL_19410271	EPI_ISL_19410271	EPI_ISL_19410271	EPI_ISL_19410271	EPI_ISL_19410271	EPI_ISL_19410271	EPI_ISL_19410271		Bolivia
A/chicken/Cochabamba/39539/2023	EPI_ISL_19418487		EPI_ISL_19418487	EPI_ISL_19418487		EPI_ISL_19418487	EPI_ISL_19418487		Bolivia
A/chicken/Cordoba/ICA-3499/2022	EPI_ISL_17353510	EPI_ISL_17353510	EPI_ISL_17353510	EPI_ISL_17353510	EPI_ISL_17353510	EPI_ISL_17353510	EPI_ISL_17353510	EPI_ISL_17353510	Argentina
A/chicken/England/063896/2022	EPI_ISL_13370925	EPI_ISL_13370925	EPI_ISL_13370925		EPI_ISL_13370925	EPI_ISL_13370925	EPI_ISL_13370925		Eurasia
A/chicken/Hokkaido/21D3T/2022	EPI_ISL_18720845	EPI_ISL_18720845	EPI_ISL_18720845	EPI_ISL_18720845	EPI_ISL_18720845	EPI_ISL_18720845	EPI_ISL_18720845	EPI_ISL_18720845	Eurasia
A/chicken/Hokkaido/24A1T/2024	EPI_ISL_19655349	EPI_ISL_19655349	EPI_ISL_19655349	EPI_ISL_19655349	EPI_ISL_19655349	EPI_ISL_19655349	EPI_ISL_19655349	EPI_ISL_19655349	Eurasia
A/chicken/Ibaraki/22B6T/2022	EPI_ISL_18286286	EPI_ISL_18286286	EPI_ISL_18286286	EPI_ISL_18286286	EPI_ISL_18286286	EPI_ISL_18286286	EPI_ISL_18286286	EPI_ISL_18286286	Eurasia
A/chicken/Kagoshima/22C2T/2022	EPI_ISL_18286307	EPI_ISL_18286307	EPI_ISL_18286307	EPI_ISL_18286307	EPI_ISL_18286307	EPI_ISL_18286307	EPI_ISL_18286307	EPI_ISL_18286307	Eurasia
A/chicken/Magadan/14-7V/2022	EPI_ISL_16618967	EPI_ISL_16618967	EPI_ISL_16618967	EPI_ISL_16618967	EPI_ISL_16618967	EPI_ISL_16618967	EPI_ISL_16618967	EPI_ISL_16618967	Eurasia
A/chicken/Magdalena/ICA-3503/2022	EPI_ISL_17353838	EPI_ISL_17353838	EPI_ISL_17353838	EPI_ISL_17353838	EPI_ISL_17353838	EPI_ISL_17353838	EPI_ISL_17353838	EPI_ISL_17353838	Argentina
A/chicken/Maule/247588-1/2023	EPI_ISL_19410280	EPI_ISL_19410280	EPI_ISL_19410280		EPI_ISL_19410280	EPI_ISL_19410280		EPI_ISL_19410280	Chile
A/chicken/Metropolitana/243148-1/2023	EPI_ISL_18760060	EPI_ISL_18760060	EPI_ISL_18760060	EPI_ISL_18760060		EPI_ISL_18760060	EPI_ISL_18760060		Chile
A/chicken/New_York/23-008875-001-original/2023	EPI_ISL_19490472	EPI_ISL_19490472	EPI_ISL_19490472	EPI_ISL_19490472		EPI_ISL_19490472	EPI_ISL_19490472		USA
A/chicken/Nuble/239136/2023	EPI_ISL_17885878	EPI_ISL_17885878	EPI_ISL_17885878	EPI_ISL_17885878		EPI_ISL_17885878	EPI_ISL_17885878	EPI_ISL_17885878	Chile
A/chicken/Nuble/240155/2023	EPI_ISL_17885877	EPI_ISL_17885877	EPI_ISL_17885877	EPI_ISL_17885877		EPI_ISL_17885877	EPI_ISL_17885877	EPI_ISL_17885877	Chile
A/chicken/Nuble/240684/2023	EPI_ISL_17885876	EPI_ISL_17885876	EPI_ISL_17885876	EPI_ISL_17885876	EPI_ISL_17885876	EPI_ISL_17885876	EPI_ISL_17885876		Chile
A/chicken/Nuble/241557-1/2023		EPI_ISL_17885874	EPI_ISL_17885874						Chile
A/chicken/Nuble/241681-1/2023			EPI_ISL_17885873		EPI_ISL_17885873				Chile
A/chicken/OHiggins/241252-1/2023			EPI_ISL_17885872		EPI_ISL_17885872	EPI_ISL_17885872			Chile
A/chicken/OHiggins/241252-3/2023		EPI_ISL_17885871	EPI_ISL_17885871	EPI_ISL_17885871		EPI_ISL_17885871	EPI_ISL_17885871		Chile
A/chicken/OHiggins/241252-6/2023	EPI_ISL_17885870	EPI_ISL_17885870	EPI_ISL_17885870				EPI_ISL_17885870		Chile
A/chicken/Peru/23-005607-001/2022	EPI_ISL_17660070	EPI_ISL_17660070		EPI_ISL_17660070				EPI_ISL_17660070	Peru
A/chicken/Peru/23-005607-002/2022		EPI_ISL_17660071				EPI_ISL_17660071		EPI_ISL_17660071	Peru
A/chicken/Peru/23-005607-003/2022	EPI_ISL_17660072	EPI_ISL_17660072		EPI_ISL_17660072	EPI_ISL_17660072	EPI_ISL_17660072		EPI_ISL_17660072	Peru
A/chicken/Peru/AIS0539/2022	EPI_ISL_17805987	EPI_ISL_17805987	EPI_ISL_17805987			EPI_ISL_17805987	EPI_ISL_17805987		Peru
A/chicken/Peru/AIS0540/2022	EPI_ISL_17805988		EPI_ISL_17805988						Peru
A/chicken/Peru/AIS0542/2022		EPI_ISL_17805989	EPI_ISL_17805989	EPI_ISL_17805989	EPI_ISL_17805989	EPI_ISL_17805989	EPI_ISL_17805989		Peru
A/chicken/Peru/AIS0543/2022	EPI_ISL_17805990								Peru
A/chicken/Peru/AIS0544/2022	EPI_ISL_17805991	EPI_ISL_17805991	EPI_ISL_17805991	EPI_ISL_17805991		EPI_ISL_17805991	EPI_ISL_17805991	EPI_ISL_17805991	Peru
A/chicken/Peru/AIS0546/2022		EPI_ISL_17805986	EPI_ISL_17805986			EPI_ISL_17805986	EPI_ISL_17805986		Peru
A/chicken/Peru/AIS0548/2022	EPI_ISL_17805994	EPI_ISL_17805994	EPI_ISL_17805994	EPI_ISL_17805994	EPI_ISL_17805994	EPI_ISL_17805994	EPI_ISL_17805994	EPI_ISL_17805994	Peru
A/chicken/Peru/AIS0550/2022			EPI_ISL_17805995						Peru
A/chicken/Peru/AIS0551/2022	EPI_ISL_17806001	EPI_ISL_17806001	EPI_ISL_17806001	EPI_ISL_17806001	EPI_ISL_17806001	EPI_ISL_17806001	EPI_ISL_17806001	EPI_ISL_17806001	Peru
A/chicken/Peru/LAM-INS-002/2022			EPI_ISL_16249681			EPI_ISL_16249681			Peru
A/chicken/Peru/LIM-INS-003/2022						EPI_ISL_16249730	EPI_ISL_16249730		Peru
A/chicken/Potosi/39870/2023					EPI_ISL_19410299	EPI_ISL_19410299			Bolivia
A/Chicken/SK/FAV-1474-1/2022	EPI_ISL_19154741	EPI_ISL_19154741	EPI_ISL_19154741	EPI_ISL_19154741	EPI_ISL_19154741	EPI_ISL_19154741	EPI_ISL_19154741		USA
A/chicken/Vietnam/RAHO7-22S-5669/2022	EPI_ISL_20065029	EPI_ISL_20065029	EPI_ISL_20065029	EPI_ISL_20065029	EPI_ISL_20065029	EPI_ISL_20065029	EPI_ISL_20065029	EPI_ISL_20065029	Eurasia
A/Chile/25945/2023	EPI_ISL_17468386	EPI_ISL_17468386	EPI_ISL_17468386	EPI_ISL_17468386		EPI_ISL_17468386	EPI_ISL_17468386	EPI_ISL_17468386	Chile
A/Chilean_teal/Nuble/233516-2/2023		EPI_ISL_19410275							Chile
A/Chiloe_wigeon/OHiggins/240893-2/2023	EPI_ISL_17885965	EPI_ISL_17885965	EPI_ISL_17885965	EPI_ISL_17885965	EPI_ISL_17885965	EPI_ISL_17885965	EPI_ISL_17885965	EPI_ISL_17885965	Chile
A/chungungo/Chile/241436/2023	EPI_ISL_19391459	EPI_ISL_19391459	EPI_ISL_19391459	EPI_ISL_19391459	EPI_ISL_19391459	EPI_ISL_19391459		EPI_ISL_19391459	Chile

A/Common_Eider/NL/FAV-1404-1/2022	EPI_ISL_19154653	EPI_ISL_19154653	EPI_ISL_19154653	EPI_ISL_19154653	EPI_ISL_19154653	EPI_ISL_19154653	EPI_ISL_19154653	EPI_ISL_19154653	USA
A/common_teal/Korea/WA537/2022	EPI_ISL_15647835	EPI_ISL_15647835	EPI_ISL_15647835	EPI_ISL_15647835	EPI_ISL_15647835	EPI_ISL_15647835	EPI_ISL_15647835	EPI_ISL_15647835	Eurasia
A/crow/Hokkaido/0101Q044/2022__H5N1_	EPI_ISL_14064697	EPI_ISL_14064697	EPI_ISL_14064697	EPI_ISL_14064697	EPI_ISL_14064697	EPI_ISL_14064697	EPI_ISL_14064697	EPI_ISL_14064697	Eurasia
A/crow/Khabarovsk/776-56/2022	EPI_ISL_18074199	EPI_ISL_18074199	EPI_ISL_18074199	EPI_ISL_18074199	EPI_ISL_18074199	EPI_ISL_18074199	EPI_ISL_18074199	EPI_ISL_18074199	Eurasia
A/dolphin/Peru/PIU-002/2022	EPI_ISL_18054503	EPI_ISL_18054503	EPI_ISL_18054503		EPI_ISL_18054503			EPI_ISL_18054503	Peru
A/dolphin/Peru/PIU-SER002/2022				EPI_ISL_18265431	EPI_ISL_18265431		EPI_ISL_18265431		Peru
A/Double_Crested_Cormorant/BC/AIVPHL-791/2022	EPI_ISL_19303634	EPI_ISL_19303634	EPI_ISL_19303634	EPI_ISL_19303634	EPI_ISL_19303634	EPI_ISL_19303634	EPI_ISL_19303634	EPI_ISL_19303634	USA
A/Duck/Argentina/1712-5/2023	EPI_ISL_18698515	EPI_ISL_18698515	EPI_ISL_18698515	EPI_ISL_18698515		EPI_ISL_18698515	EPI_ISL_18698515		Argentina
A/duck/Choco/ICA-3501/2022	EPI_ISL_17353839	EPI_ISL_17353839	EPI_ISL_17353839	EPI_ISL_17353839	EPI_ISL_17353839	EPI_ISL_17353839	EPI_ISL_17353839	EPI_ISL_17353839	Argentina
A/duck/Hawaii/24-034987-004/2024	EPI_ISL_19678913	EPI_ISL_19678913	EPI_ISL_19678913	EPI_ISL_19678913		EPI_ISL_19678913	EPI_ISL_19678913	EPI_ISL_19678913	USA
A/duck/Korea/H625/2022	EPI_ISL_19453461	EPI_ISL_19453461	EPI_ISL_19453461	EPI_ISL_19453461	EPI_ISL_19453461	EPI_ISL_19453461	EPI_ISL_19453461	EPI_ISL_19453461	Eurasia
A/duck/Peru/CAL-INS-013/2023		EPI_ISL_18217104	EPI_ISL_18217104	EPI_ISL_18217104	EPI_ISL_18217104	EPI_ISL_18217104	EPI_ISL_18217104	EPI_ISL_18217104	Peru
A/duck/Peru/LAM-INS-014/2023	EPI_ISL_18497946	EPI_ISL_18497946	EPI_ISL_18497946	EPI_ISL_18497946	EPI_ISL_18497946	EPI_ISL_18497946	EPI_ISL_18497946	EPI_ISL_18497946	Peru
A/duck/Peru/LAM-INS-015/2023					EPI_ISL_18497950			EPI_ISL_18497950	Peru
A/duck/Vietnam/Raho7-7-23-3-404/2023	EPI_ISL_20069768	EPI_ISL_20069768	EPI_ISL_20069768	EPI_ISL_20069768	EPI_ISL_20069768	EPI_ISL_20069768	EPI_ISL_20069768	EPI_ISL_20069768	Eurasia
A/Elegant_tern/Arica_y_Parinacota/229476-1/2022	EPI_ISL_17885955			EPI_ISL_17885955		EPI_ISL_17885955			Chile
A/emu/Iwate/21B1T/2022	EPI_ISL_18720867	EPI_ISL_18720867	EPI_ISL_18720867	EPI_ISL_18720867	EPI_ISL_18720867	EPI_ISL_18720867	EPI_ISL_18720867	EPI_ISL_18720867	Eurasia
A/environment/Chile/C62044/2022	EPI_ISL_19167924		EPI_ISL_19167924				EPI_ISL_19167924		Chile
A/environment/Chile/C62069/2022	EPI_ISL_19167926	EPI_ISL_19167926	EPI_ISL_19167926	EPI_ISL_19167926	EPI_ISL_19167926	EPI_ISL_19167926	EPI_ISL_19167926	EPI_ISL_19167926	Chile
A/environment/Kagoshima/KU-G2/2023__H5N1_	EPI_ISL_18615599	EPI_ISL_18615599	EPI_ISL_18615599	EPI_ISL_18615599	EPI_ISL_18615599	EPI_ISL_18615599	EPI_ISL_18615599	EPI_ISL_18615599	Eurasia
A/Eurasian_wigeon/Germany-SH/AI05950/2021	EPI_ISL_7748188	EPI_ISL_7748188	EPI_ISL_7748188	EPI_ISL_7748188	EPI_ISL_7748188	EPI_ISL_7748188	EPI_ISL_7748188	EPI_ISL_7748188	Eurasia
A/Franklin_gull/OHiggins/236195-1/2023	EPI_ISL_19162729	EPI_ISL_19162729	EPI_ISL_19162729	EPI_ISL_19162729	EPI_ISL_19162729	EPI_ISL_19162729	EPI_ISL_19162729	EPI_ISL_19162729	Chile
A/frigatebird/Rio_de_Janeiro/MAPA-1532N/2023	EPI_ISL_18755251	EPI_ISL_18755251	EPI_ISL_18755251	EPI_ISL_18755251		EPI_ISL_18755251			Brazil
A/gadwall/Alaska/23-006279-001/2023	EPI_ISL_19607986	EPI_ISL_19607986	EPI_ISL_19607986	EPI_ISL_19607986	EPI_ISL_19607986	EPI_ISL_19607986	EPI_ISL_19607986	EPI_ISL_19607986	USA
A/Gallus_gallus/Mato_Grosso_do_Sul/2108-SN52/2023	EPI_ISL_19215176	EPI_ISL_19215176	EPI_ISL_19215176	EPI_ISL_19215176	EPI_ISL_19215176	EPI_ISL_19215176	EPI_ISL_19215176	EPI_ISL_19215176	Brazil
A/Gallus_gallus/Santo_Antonio_da_Barra/1611-N3/2025	EPI_ISL_20267521	EPI_ISL_20267521	EPI_ISL_20267521	EPI_ISL_20267521	EPI_ISL_20267521	EPI_ISL_20267521	EPI_ISL_20267521		Chile
A/Gallus_gallus/Sao_Paulo/2000-N2/2025	EPI_ISL_20267522	EPI_ISL_20267522	EPI_ISL_20267522	EPI_ISL_20267522	EPI_ISL_20267522	EPI_ISL_20267522	EPI_ISL_20267522	EPI_ISL_20267522	Brazil
A/glaucous_gull/Alaska/22-031916-001/2022	EPI_ISL_19158296	EPI_ISL_19158296	EPI_ISL_19158296	EPI_ISL_19158296		EPI_ISL_19158296	EPI_ISL_19158296	EPI_ISL_19158296	USA
A/glaucous_gull/Alaska/23-025003-001/2023	EPI_ISL_18737452	EPI_ISL_18737452	EPI_ISL_18737452	EPI_ISL_18737452	EPI_ISL_18737452	EPI_ISL_18737452	EPI_ISL_18737452	EPI_ISL_18737452	USA
A/Glaucous-winged_gull/Washington/W231990001-1/2023	EPI_ISL_19592595	EPI_ISL_19592595	EPI_ISL_19592595	EPI_ISL_19592595	EPI_ISL_19592595	EPI_ISL_19592595	EPI_ISL_19592595	EPI_ISL_19592595	USA
A/goose/Araucania/239189-1/2023	EPI_ISL_17885869	EPI_ISL_17885869	EPI_ISL_17885869	EPI_ISL_17885869		EPI_ISL_17885869	EPI_ISL_17885869		Chile
A/Goose/Argentina/389-1/2023	EPI_ISL_18698459	EPI_ISL_18698459	EPI_ISL_18698459	EPI_ISL_18698459	EPI_ISL_18698459	EPI_ISL_18698459	EPI_ISL_18698459	EPI_ISL_18698459	Argentina
A/goose/Barueri/LEIAL5803/2025	EPI_ISL_20078350	EPI_ISL_20078350	EPI_ISL_20078350	EPI_ISL_20078350	EPI_ISL_20078350	EPI_ISL_20078350	EPI_ISL_20078350	EPI_ISL_20078350	Brazil
A/goose/Kentucky/22-040946-001/2022	EPI_ISL_19605291	EPI_ISL_19605291	EPI_ISL_19605291	EPI_ISL_19605291		EPI_ISL_19605291	EPI_ISL_19605291	EPI_ISL_19605291	USA
A/goose/Utah/23-001041-008/2023	EPI_ISL_19605258	EPI_ISL_19605258	EPI_ISL_19605258	EPI_ISL_19605258	EPI_ISL_19605258	EPI_ISL_19605258	EPI_ISL_19605258	EPI_ISL_19605258	USA
A/gray_gull/Arica/239053-1/2023	EPI_ISL_19410281	EPI_ISL_19410281	EPI_ISL_19410281	EPI_ISL_19410281	EPI_ISL_19410281	EPI_ISL_19410281	EPI_ISL_19410281	EPI_ISL_19410281	Chile
A/gray_gull/Chile/C61947/2022	EPI_ISL_16891400			EPI_ISL_16891400		EPI_ISL_16891400		EPI_ISL_16891400	Chile
A/Gray_gull/Tarapaca/232825-1/2023	EPI_ISL_17885951	EPI_ISL_17885951	EPI_ISL_17885951			EPI_ISL_17885951	EPI_ISL_17885951	EPI_ISL_17885951	Chile
A/great_black_backed_gull/Massachusetts/23-004612-027/2023	EPI_ISL_19607353	EPI_ISL_19607353	EPI_ISL_19607353		EPI_ISL_19607353	EPI_ISL_19607353	EPI_ISL_19607353		USA
A/Great_Black-Backed_Gull/NL/OTH-0114	EPI_ISL_16190849	EPI_ISL_16190849	EPI_ISL_16190849	EPI_ISL_16190849	EPI_ISL_16190849	EPI_ISL_16190849	EPI_ISL_16190849	EPI_ISL_16190849	USA
A/Great_egret/Araucania/240518/2023		EPI_ISL_17885950	EPI_ISL_17885950			EPI_ISL_17885950	EPI_ISL_17885950	EPI_ISL_17885950	Chile
A/Great_grabe/Atacama/231482-2/2023		EPI_ISL_19162731	EPI_ISL_19162731			EPI_ISL_19162731	EPI_ISL_19162731	EPI_ISL_19162731	Chile
A/great_horned_owl/Colorado/23-004706-001/2023	EPI_ISL_17964849	EPI_ISL_17964849	EPI_ISL_17964849	EPI_ISL_17964849		EPI_ISL_17964849	EPI_ISL_17964849	EPI_ISL_17964849	USA
A/great_horned_owl/Massachusetts/015884-023/2023			EPI_ISL_19736856						USA
A/Great_skua/Norway/2022-07-1486-2/2022	EPI_ISL_18901634	EPI_ISL_18901634	EPI_ISL_18901634	EPI_ISL_18901634	EPI_ISL_18901634	EPI_ISL_18901634	EPI_ISL_18901634	EPI_ISL_18901634	Eurasia
A/great_skua/Scotland/042505/2021						EPI_ISL_6029360			Eurasia
A/guanay_cormorant/Atacama/235520-1/2023	EPI_ISL_19151400	EPI_ISL_19151400	EPI_ISL_19151400		EPI_ISL_19151400	EPI_ISL_19151400		EPI_ISL_19151400	Chile
A/Guanay_cormorant/Chile/239584/2023	EPI_ISL_19391460	EPI_ISL_19391460	EPI_ISL_19391460	EPI_ISL_19391460		EPI_ISL_19391460	EPI_ISL_19391460		Chile
A/guanay_cormorant/Peru/CAL-INS-009/2023	EPI_ISL_17777530	EPI_ISL_17777530	EPI_ISL_17777530	EPI_ISL_17777530	EPI_ISL_17777530	EPI_ISL_17777530	EPI_ISL_17777530	EPI_ISL_17777530	Peru
A/guanay_cormorant/Peru/PIU-024/2022						EPI_ISL_18054500			Peru
A/Guanay_cormorant/Peru/UNMSM-A275/2022	EPI_ISL_18371665	EPI_ISL_18371665				EPI_ISL_18371665	EPI_ISL_18371665		Peru
A/gull/Biobio/237012/2023		EPI_ISL_19410300				EPI_ISL_19410300	EPI_ISL_19410300	EPI_ISL_19410300	Chile
A/Gull/Chile/227023-2/2022	EPI_ISL_17011964	EPI_ISL_17011964	EPI_ISL_17011964	EPI_ISL_17011964		EPI_ISL_17011964			Chile
A/Gull/Chile/227023-3/2022	EPI_ISL_17011958	EPI_ISL_17011958	EPI_ISL_17011958	EPI_ISL_17011958	EPI_ISL_17011958	EPI_ISL_17011958			Chile
A/Gull/Norway/2022-07-3180-1/2022	EPI_ISL_18901673	EPI_ISL_18901673	EPI_ISL_18901673	EPI_ISL_18901673	EPI_ISL_18901673	EPI_ISL_18901673	EPI_ISL_18901673	EPI_ISL_18901673	Eurasia
A/gull/Oregon/23-000764-001/2023	EPI_ISL_19607562	EPI_ISL_19607562	EPI_ISL_19607562	EPI_ISL_19607562	EPI_ISL_19607562	EPI_ISL_19607562	EPI_ISL_19607562	EPI_ISL_19607562	USA
A/gull/Peru/LIM-INS-006/2023	EPI_ISL_17777527	EPI_ISL_17777527	EPI_ISL_17777527		EPI_ISL_17777527	EPI_ISL_17777527		EPI_ISL_17777527	Peru
A/herring_gull/Massachusetts/23-001397-012/2022	EPI_ISL_19607542	EPI_ISL_19607542	EPI_ISL_19607542	EPI_ISL_19607542	EPI_ISL_19607542	EPI_ISL_19607542	EPI_ISL_19607542	EPI_ISL_19607542	USA
A/Hooded_crane/Kagoshima/KU-24-2/2024__H5N1_	EPI_ISL_19624025	EPI_ISL_19624025	EPI_ISL_19624025	EPI_ISL_19624025	EPI_ISL_19624025	EPI_ISL_19624025	EPI_ISL_19624025		Eurasia
A/Hooded_Merganser/BC/AIVPHL-2033/2024	EPI_ISL_19303642	EPI_ISL_19303642	EPI_ISL_19303642	EPI_ISL_19303642	EPI_ISL_19303642	EPI_ISL_19303642	EPI_ISL_19303642	EPI_ISL_19303642	USA
A/Humboldt_penguin/Coquimbo/239590/2023	EPI_ISL_17885948		EPI_ISL_17885948		EPI_ISL_17885948	EPI_ISL_17885948	EPI_ISL_17885948	EPI_ISL_17885948	Chile
A/Inca_tern/Antofagasta/238083/2023	EPI_ISL_19131156	EPI_ISL_19131156			EPI_ISL_19131156	EPI_ISL_19131156	EPI_ISL_19131156	EPI_ISL_19131156	Chile
A/Inca_tern/Arica_y_Parinacota/227519-1/2022		EPI_ISL_19131159	EPI_ISL_19131159	EPI_ISL_19131159		EPI_ISL_19131159	EPI_ISL_19131159		Chile
A/Inca_tern/Coquimbo/239619/2023	EPI_ISL_19131162		EPI_ISL_19131162			EPI_ISL_19131162	EPI_ISL_19131162		Chile
A/kelp_gull/Antartica/O2601-CL/2024	EPI_ISL_19589149	EPI_ISL_19589149	EPI_ISL_19589149	EPI_ISL_19589149	EPI_ISL_19589149	EPI_ISL_19589149	EPI_ISL_19589149		Antartica
A/Kelp_Gull/Harpon_Bay/133943/2023	EPI_ISL_18592427	EPI_ISL_18592427		EPI_ISL_18592427		EPI_ISL_18592427	EPI_ISL_18592427		Antartica
A/Kelp_Gull/Hound_Bay/133744/2023	EPI_ISL_18592422	EPI_ISL_18592422	EPI_ISL_18592422	EPI_ISL_18592422	EPI_ISL_18592422	EPI_ISL_18592422	EPI_ISL_18592422		Antartica
A/Kelp_Gull/Hound_Bay/133747/2023			EPI_ISL_18592423	EPI_ISL_18592423	EPI_ISL_18592423	EPI_ISL_18592423	EPI_ISL_18592423		Antartica
A/kelp_gull/Kerguelen/24P023440/2024					EPI_ISL_19747219	EPI_ISL_19747219	EPI_ISL_19747219	EPI_ISL_19747219	Antartica
A/Kelp_gull/Maule/239349/2023	EPI_ISL_17885972	EPI_ISL_17885972	EPI_ISL_17885972	EPI_ISL_17885972	EPI_ISL_17885972	EPI_ISL_17885972	EPI_ISL_17885972		Chile
A/Kelp_Gull/Moltke_Harbour/133754/2023	EPI_ISL_18592425		EPI_ISL_18592425			EPI_ISL_18592425	EPI_ISL_18592425	EPI_ISL_18592425	Antartica
A/Kelp_Gull/Penguin_River/141234/2023	EPI_ISL_18742215	EPI_ISL_18742215		EPI_ISL_18742215	EPI_ISL_18742215	EPI_ISL_18742215		EPI_ISL_18742215	Antartica
A/Kelp_Gull/Penguin_River/141239/2023		EPI_ISL_18742217				EPI_ISL_18742217			Antartica
A/king_penguin/Crozet/24P021412/2024	EPI_ISL_19747201	EPI_ISL_19747201	EPI_ISL_19747201		EPI_ISL_19747201	EPI_ISL_19747201	EPI_ISL_19747201		Antartica
A/king_penguin/Crozet/24P021416/2024	EPI_ISL_19747202	EPI_ISL_19747202	EPI_ISL_19747202			EPI_ISL_19747202		EPI_ISL_19747202	Antartica
A/king_penguin/Crozet/24P023398/2024				EPI_ISL_19747213	EPI_ISL_19747213	EPI_ISL_19747213	EPI_ISL_19747213	EPI_ISL_19747213	Antartica
A/king_penguin/Crozet/24P023407/2024						EPI_ISL_19747212	EPI_ISL_19747212	EPI_ISL_19747212	Antartica
A/king_penguin/Crozet/24P023414/2024				EPI_ISL_19747214		EPI_ISL_19747214	EPI_ISL_19747214		Antartica
A/large-billed_crow/Hokkaido/0103E089/2023	EPI_ISL_17950253	EPI_ISL_17950253	EPI_ISL_17950253	EPI_ISL_17950253	EPI_ISL_17950253	EPI_ISL_17950253	EPI_ISL_17950253	EPI_ISL_17950253	Eurasia
A/lion/Peru/AIS0554/2023	EPI_ISL_17805999	EPI_ISL_17805999	EPI_ISL_17805999	EPI_ISL_17805999		EPI_ISL_17805999	EPI_ISL_17805999	EPI_ISL_17805999	Peru
A/mallard/Michigan/22-024975-001/2022	EPI_ISL_19604100	EPI_ISL_19604100	EPI_ISL_19604100	EPI_ISL_19604100	EPI_ISL_19604100	EPI_ISL_19604100	EPI_ISL_19604100	EPI_ISL_19604100	USA
A/Mallard/Tennessee/767/2025	EPI_ISL_20082756	EPI_ISL_20082756	EPI_ISL_20082756	EPI_ISL_20082756	EPI_ISL_20082756	EPI_ISL_20082756	EPI_ISL_20082756	EPI_ISL_20082756	USA
A/mandarin_duck/Korea/WA585/2021	EPI_ISL_6959592	EPI_ISL_6959592	EPI_ISL_6959592	EPI_ISL_6959592	EPI_ISL_6959592	EPI_ISL_6959592	EPI_ISL_6959592	EPI_ISL_6959592	Eurasia
A/Megascops_choliba/Espirito_Santo/1409-N/2023	EPI_ISL_19215177			EPI_ISL_19215177			EPI_ISL_19215177		Brazil
A/mute_swan/Austria/22007848-001/2022	EPI_ISL_17785732	EPI_ISL_17785732	EPI_ISL_17785732	EPI_ISL_17785732	EPI_ISL_17785732	EPI_ISL_17785732	EPI_ISL_17785732	EPI_ISL_17785732	Eurasia
A/Nasua_nasua/Uruguay/145M1/2023	EPI_ISL_19765465	EPI_ISL_19765465	EPI_ISL_19765465			EPI_ISL_19765465	EPI_ISL_19765465	EPI_ISL_19765465	Uruguay
A/Nasua_nasua/Uruguay/146M1/2023	EPI_ISL_19765485			EPI_ISL_19765485		EPI_ISL_19765485	EPI_ISL_19765485		Uruguay
A/Nasua_nasua/Uruguay/145M2/2023					EPI_ISL_19765475				Uruguay
A/Northern_fur_seal/Russia_Tyuleniy/74-2/2023	EPI_ISL_19080209	EPI_ISL_19080209	EPI_ISL_19080209	EPI_ISL_19080209	EPI_ISL_19080209	EPI_ISL_19080209	EPI_ISL_19080209	EPI_ISL_19080209	Antarctica
A/Northern_goshawk/Norway/2023-07-77-1/2023		EPI_ISL_18963398	EPI_ISL_18963398	EPI_ISL_18963398	EPI_ISL_18963398	EPI_ISL_18963398	EPI_ISL_18963398	EPI_ISL_18963398	Eurasia
A/northern_pintail/Alaska/22-029100-001/2022	EPI_ISL_19158326	EPI_ISL_19158326	EPI_ISL_19158326	EPI_ISL_19158326	EPI_ISL_19158326	EPI_ISL_19158326	EPI_ISL_19158326	EPI_ISL_19158326	USA
A/northern_pintail/USA/IZ24_0636/2024	EPI_ISL_19871011	EPI_ISL_19871011	EPI_ISL_19871011	EPI_ISL_19871011	EPI_ISL_19871011	EPI_ISL_19871011	EPI_ISL_19871011	EPI_ISL_19871011	USA
A/northern_shoveler/Alaska/23-003641-007/2022	EPI_ISL_19604369	EPI_ISL_19604369	EPI_ISL_19604369	EPI_ISL_19604369	EPI_ISL_19604369	EPI_ISL_19604369	EPI_ISL_19604369	EPI_ISL_19604369	USA
A/Northern_shoveler/Jeju/D60/2023	EPI_ISL_19035743	EPI_ISL_19035743	EPI_ISL_19035743	EPI_ISL_19035743		EPI_ISL_19035743	EPI_ISL_19035743	EPI_ISL_19035743	Eurasia
A/Numida_meleagris/Rio_de_Janeiro/2148-N/2025	EPI_ISL_20267523	EPI_ISL_20267523	EPI_ISL_20267523	EPI_ISL_20267523	EPI_ISL_20267523	EPI_ISL_20267523	EPI_ISL_20267523		Brazil
A/Numida_meleagris/Santa_Catarina/1843-N3/2023	EPI_ISL_19215178	EPI_ISL_19215178	EPI_ISL_19215178	EPI_ISL_19215178		EPI_ISL_19215178	EPI_ISL_19215178	EPI_ISL_19215178	Brazil
A/Otaria_flavescens/Rio_Grande_do_Sul/2148-N/2023	EPI_ISL_19215179	EPI_ISL_19215179	EPI_ISL_19215179	EPI_ISL_19215179	EPI_ISL_19215179	EPI_ISL_19215179	EPI_ISL_19215179		Brazil
A/Otaria_flavescens/Rio_Grande_do_Sul/2165-SO/2023		EPI_ISL_19215180	EPI_ISL_19215180	EPI_ISL_19215180		EPI_ISL_19215180	EPI_ISL_19215180		Brazil
A/owl/Peru/23-005629-001/2022	EPI_ISL_17660073		EPI_ISL_17660073	EPI_ISL_17660073	EPI_ISL_17660073	EPI_ISL_17660073	EPI_ISL_17660073		Peru
A/Pelecanus_occidentalis/Venezuela/3S2/2022		EPI_ISL_16854395							Venezuela
A/Pelecanus_occidentalis/Venezuela/3S3/2022			EPI_ISL_16854396						Venezuela
A/Pelecanus_occidentalis/Venezuela/3S4/2022						EPI_ISL_16854397			Venezuela
A/Pelecanus_occidentalis/Venezuela/3S5/2022				EPI_ISL_16854398					Venezuela
A/Pelecanus_occidentalis/Venezuela/3S7/2022					EPI_ISL_16854400				Venezuela
A/Pelecanus_occidentalis/Venezuela/3S8/2022								EPI_ISL_16854401	Venezuela
A/Pelecanus_occidentalis/Venezuela/4S3/2022			EPI_ISL_16854404						Venezuela
A/Pelecanus_occidentalis/Venezuela/4S4/2022						EPI_ISL_16854405			Venezuela
A/Pelecanus_occidentalis/Venezuela/4S7/2022					EPI_ISL_16854408				Venezuela
A/Pelecanus_occidentalis/Venezuela/4S8/2022								EPI_ISL_16854409	Venezuela
A/Pelecanus_occidentalis/Venezuela/Inf1/2022		EPI_ISL_18909019	EPI_ISL_18909019			EPI_ISL_18909019	EPI_ISL_18909019		Venezuela
A/Pelecanus_occidentalis/Venezuela/Inf13/2022		EPI_ISL_16701840						EPI_ISL_16701840	Venezuela
A/Pelecanus_occidentalis/Venezuela/Inf14/2022		EPI_ISL_18931268					EPI_ISL_18931268		Venezuela
A/Pelecanus_occidentalis/Venezuela/Inf19/2022		EPI_ISL_18931271	EPI_ISL_18931271			EPI_ISL_18931271	EPI_ISL_18931271		Venezuela
A/Pelecanus_occidentalis/Venezuela/Inf20/2022			EPI_ISL_18931272	EPI_ISL_18931272	EPI_ISL_18931272		EPI_ISL_18931272	EPI_ISL_18931272	Venezuela
A/Pelecanus_occidentalis/Venezuela/Inf41/2023		EPI_ISL_18931274		EPI_ISL_18931274		EPI_ISL_18931274	EPI_ISL_18931274	EPI_ISL_18931274	Venezuela
A/Pelecanus_occidentalis/Venezuela/Inf44/2023				EPI_ISL_18931276		EPI_ISL_18931276	EPI_ISL_18931276	EPI_ISL_18931276	Venezuela
A/Pelecanus_thagus/Peru/LIM-QUIM006/2023						EPI_ISL_19781426			Peru
A/Pelecanus/Peru/VFAR-140/2022	EPI_ISL_17099964	EPI_ISL_17099964	EPI_ISL_17099964		EPI_ISL_17099964	EPI_ISL_17099964		EPI_ISL_17099964	Peru
A/Pelican/Antofagasta/228244-2/2022	EPI_ISL_17885944	EPI_ISL_17885944	EPI_ISL_17885944	EPI_ISL_17885944			EPI_ISL_17885944		Chile
A/Pelican/Antofagasta/228246-2/2022		EPI_ISL_17885934		EPI_ISL_17885934	EPI_ISL_17885934	EPI_ISL_17885934	EPI_ISL_17885934		Chile
A/Pelican/Antofagasta/228246-3/2022	EPI_ISL_17885931	EPI_ISL_17885931	EPI_ISL_17885931			EPI_ISL_17885931	EPI_ISL_17885931	EPI_ISL_17885931	Chile
A/pelican/Antofagasta/228318-1/2022	EPI_ISL_17885928	EPI_ISL_17885928	EPI_ISL_17885928			EPI_ISL_17885928	EPI_ISL_17885928	EPI_ISL_17885928	Chile
A/Pelican/Atacama/229424-2/2022		EPI_ISL_17885927		EPI_ISL_17885927	EPI_ISL_17885927	EPI_ISL_17885927			Chile
A/Pelican/Atacama/229450-2/2022	EPI_ISL_17885926	EPI_ISL_17885926	EPI_ISL_17885926	EPI_ISL_17885926		EPI_ISL_17885926			Chile
A/pelican/Bio_Bio/236574/2023	EPI_ISL_18760061	EPI_ISL_18760061				EPI_ISL_18760061			Chile
A/pelican/Chile/229424-4/2022	EPI_ISL_19391461	EPI_ISL_19391461	EPI_ISL_19391461				EPI_ISL_19391461	EPI_ISL_19391461	Chile
A/Pelican/Chile/6618-1/2022	EPI_ISL_17011962	EPI_ISL_17011962	EPI_ISL_17011962	EPI_ISL_17011962					Chile
A/Pelican/Chile/6618-2/2022	EPI_ISL_17011961	EPI_ISL_17011961	EPI_ISL_17011961	EPI_ISL_17011961			EPI_ISL_17011961	EPI_ISL_17011961	Chile
A/Pelican/Chile/6924-1/2022	EPI_ISL_17011963	EPI_ISL_17011963	EPI_ISL_17011963	EPI_ISL_17011963	EPI_ISL_17011963	EPI_ISL_17011963	EPI_ISL_17011963		Chile
A/Pelican/Chile/6955-3/2022	EPI_ISL_17011957	EPI_ISL_17011957		EPI_ISL_17011957		EPI_ISL_17011957			Chile
A/Pelican/Chile/6958-1/2022			EPI_ISL_17011959						Chile
A/Pelican/Chile/7087-1/2022			EPI_ISL_17012018		EPI_ISL_17012018	EPI_ISL_17012018	EPI_ISL_17012018		Chile
A/Pelican/Missouri/USDA-012338-019/2022	EPI_ISL_18132379	EPI_ISL_18132379	EPI_ISL_18132379	EPI_ISL_18132379	EPI_ISL_18132379	EPI_ISL_18132379	EPI_ISL_18132379	EPI_ISL_18132379	USA
A/Pelican/Nuble/233947-2/2023	EPI_ISL_17885918	EPI_ISL_17885918	EPI_ISL_17885918	EPI_ISL_17885918		EPI_ISL_17885918	EPI_ISL_17885918		Chile
A/Pelican/OHiggins/233663-1/2023	EPI_ISL_17885915	EPI_ISL_17885915	EPI_ISL_17885915	EPI_ISL_17885915		EPI_ISL_17885915			Chile
A/Pelican/OHiggins/233721-1/2023	EPI_ISL_17885912	EPI_ISL_17885912		EPI_ISL_17885912					Chile
A/pelican/Peru/AIS0538/2022		EPI_ISL_17806000	EPI_ISL_17806000	EPI_ISL_17806000	EPI_ISL_17806000	EPI_ISL_17806000		EPI_ISL_17806000	Peru
A/pelican/Peru/AIS0541/2022		EPI_ISL_17806002	EPI_ISL_17806002	EPI_ISL_17806002	EPI_ISL_17806002		EPI_ISL_17806002	EPI_ISL_17806002	Peru
A/pelican/Peru/AISA0464/2022	EPI_ISL_17806003		EPI_ISL_17806003						Peru
A/pelican/Peru/PIU-013/2022	EPI_ISL_18054504	EPI_ISL_18054504		EPI_ISL_18054504	EPI_ISL_18054504			EPI_ISL_18054504	Peru
A/pelican/Peru/PIU-019/2022							EPI_ISL_18054506		Peru
A/pelican/Peru/PIU-028/2022	EPI_ISL_18054507						EPI_ISL_18054507	EPI_ISL_18054507	Peru
A/pelican/Peru/PIU-SER016/2022	EPI_ISL_18265437	EPI_ISL_18265437					EPI_ISL_18265437	EPI_ISL_18265437	Peru
A/Pelican/Tarapaca/227436-2/2022	EPI_ISL_17885942		EPI_ISL_17885942	EPI_ISL_17885942		EPI_ISL_17885942	EPI_ISL_17885942	EPI_ISL_17885942	Chile
A/pelican/Valparaiso/233091-1/2023	EPI_ISL_17885930	EPI_ISL_17885930	EPI_ISL_17885930					EPI_ISL_17885930	Chile
A/pelican/Valparaiso/233091-2/2023	EPI_ISL_17885956	EPI_ISL_17885956	EPI_ISL_17885956			EPI_ISL_17885956	EPI_ISL_17885956		Chile
A/pelican/Valparaiso/233418-1/2023	EPI_ISL_17885932	EPI_ISL_17885932	EPI_ISL_17885932	EPI_ISL_17885932	EPI_ISL_17885932	EPI_ISL_17885932	EPI_ISL_17885932		Chile
A/pelican/Valparaiso/233450-1/2023	EPI_ISL_17885983	EPI_ISL_17885983	EPI_ISL_17885983		EPI_ISL_17885983	EPI_ISL_17885983	EPI_ISL_17885983	EPI_ISL_17885983	Chile
A/pelican/Valparaiso/234040/2023	EPI_ISL_17885982	EPI_ISL_17885982							Chile
A/Pelican/Venezuela/Inf9/2022		EPI_ISL_16631973			EPI_ISL_16631973	EPI_ISL_16631973	EPI_ISL_16631973		Venezuela
A/Pelican/Venezuela/Pel3/2022	EPI_ISL_16013752						EPI_ISL_16013752		Venezuela
A/Pelican/Venezuela/Pel4/2022				EPI_ISL_16013753			EPI_ISL_16013753		Venezuela
A/peregrine_falcon/Antofagasta/235144-1/2023	EPI_ISL_19410283	EPI_ISL_19410283	EPI_ISL_19410283	EPI_ISL_19410283	EPI_ISL_19410283			EPI_ISL_19410283	Chile
A/Peregrine_Falcon/Korea/23WC224/2024	EPI_ISL_20051140	EPI_ISL_20051140	EPI_ISL_20051140	EPI_ISL_20051140	EPI_ISL_20051140	EPI_ISL_20051140	EPI_ISL_20051140	EPI_ISL_20051140	Eurasia
A/peregrine_falcon/NewYork/160820/2022	EPI_ISL_19075358	EPI_ISL_19075358	EPI_ISL_19075358	EPI_ISL_19075358		EPI_ISL_19075358	EPI_ISL_19075358	EPI_ISL_19075358	USA
A/Peruvean_booby/Atacama/230579-1/2023	EPI_ISL_19139974	EPI_ISL_19139974	EPI_ISL_19139974	EPI_ISL_19139974	EPI_ISL_19139974	EPI_ISL_19139974			Chile
A/Peruvean_booby/Valparaiso/238535/2023	EPI_ISL_19131163	EPI_ISL_19131163	EPI_ISL_19131163				EPI_ISL_19131163	EPI_ISL_19131163	Chile
A/peruvian_booby/Coquimbo/242304/2023	EPI_ISL_19410277	EPI_ISL_19410277			EPI_ISL_19410277	EPI_ISL_19410277		EPI_ISL_19410277	Chile
A/Peruvian_booby/OHiggins/242755-1/2023	EPI_ISL_18760067	EPI_ISL_18760067	EPI_ISL_18760067	EPI_ISL_18760067		EPI_ISL_18760067	EPI_ISL_18760067		Chile
A/peruvian_booby/Peru/CAL-INS-007/2023	EPI_ISL_17777528	EPI_ISL_17777528	EPI_ISL_17777528	EPI_ISL_17777528		EPI_ISL_17777528	EPI_ISL_17777528	EPI_ISL_17777528	Peru
A/peruvian_booby/Peru/CAL-INS-008/2023	EPI_ISL_17777529	EPI_ISL_17777529	EPI_ISL_17777529		EPI_ISL_17777529	EPI_ISL_17777529	EPI_ISL_17777529	EPI_ISL_17777529	Peru
A/peruvian_booby/Peru/LIM-INS-004/2023	EPI_ISL_17777525	EPI_ISL_17777525		EPI_ISL_17777525	EPI_ISL_17777525	EPI_ISL_17777525	EPI_ISL_17777525	EPI_ISL_17777525	Peru
A/peruvian_booby/Peru/LIM-INS-005/2023				EPI_ISL_17777526	EPI_ISL_17777526		EPI_ISL_17777526	EPI_ISL_17777526	Peru
A/peruvian_booby/Peru/LIM-INS-012/2023				EPI_ISL_17777533	EPI_ISL_17777533	EPI_ISL_17777533		EPI_ISL_17777533	Peru
A/Peruvian_booby/Peru/UNMSM-A296/2022	EPI_ISL_18371666	EPI_ISL_18371666	EPI_ISL_18371666	EPI_ISL_18371666		EPI_ISL_18371666	EPI_ISL_18371666	EPI_ISL_18371666	Peru
A/pinniped/Uruguay/P10_8923/2023	EPI_ISL_19070481					EPI_ISL_19070481		EPI_ISL_19070481	Uruguay
A/pinniped/Uruguay/P14_11923/2023		EPI_ISL_19070483	EPI_ISL_19070483			EPI_ISL_19070483			Uruguay
A/pinniped/Uruguay/P17_14923/2023	EPI_ISL_19070485	EPI_ISL_19070485	EPI_ISL_19070485				EPI_ISL_19070485		Uruguay
A/pinniped/Uruguay/P18_14923/2023	EPI_ISL_19070486	EPI_ISL_19070486	EPI_ISL_19070486	EPI_ISL_19070486		EPI_ISL_19070486			Uruguay
A/pinniped/Uruguay/P4_6923/2023		EPI_ISL_19070498	EPI_ISL_19070498	EPI_ISL_19070498	EPI_ISL_19070498		EPI_ISL_19070498	EPI_ISL_19070498	Uruguay
A/pinniped/Uruguay/P8_8923/2023	EPI_ISL_19070492	EPI_ISL_19070492	EPI_ISL_19070492	EPI_ISL_19070492	EPI_ISL_19070492	EPI_ISL_19070492		EPI_ISL_19070492	Uruguay
A/pintado_petrel/Antarctica/O3121-OR/2024			EPI_ISL_19879313			EPI_ISL_19879313		EPI_ISL_19879313	Antarctica
A/pintado_petrel/Antarctica/O3121-CL/2024					EPI_ISL_19879314				Antarctica
A/Pluvialis_dominica/Sao_Paulo/2252-N/2023	EPI_ISL_19215181	EPI_ISL_19215181	EPI_ISL_19215181	EPI_ISL_19215181	EPI_ISL_19215181	EPI_ISL_19215181	EPI_ISL_19215181	EPI_ISL_19215181	Brazil
A/Procellaria_aequinoctialis/Sao_Paulo/2259-N/2023	EPI_ISL_19215182	EPI_ISL_19215182	EPI_ISL_19215182	EPI_ISL_19215182	EPI_ISL_19215182	EPI_ISL_19215182	EPI_ISL_19215182		Brazil
A/Procellaria_aequinoctialis/Sao_Paulo/2271-N/2023								EPI_ISL_19215190	Brazil
A/Raptor/Norway/2022-07-492-1/2022	EPI_ISL_18901682	EPI_ISL_18901682	EPI_ISL_18901682	EPI_ISL_18901682		EPI_ISL_18901682	EPI_ISL_18901682		Eurasia
A/Red_fox/Germany/51023-60/2023	EPI_ISL_17880904	EPI_ISL_17880904	EPI_ISL_17880904	EPI_ISL_17880904	EPI_ISL_17880904	EPI_ISL_17880904	EPI_ISL_17880904	EPI_ISL_17880904	Eurasia
A/red-tailed_hawk/Kentucky/W23-143/2022	EPI_ISL_17424631	EPI_ISL_17424631	EPI_ISL_17424631	EPI_ISL_17424631	EPI_ISL_17424631	EPI_ISL_17424631		EPI_ISL_17424631	USA
A/red-tailed_hawk/New_York/23-005536-001/2023	EPI_ISL_17964857	EPI_ISL_17964857	EPI_ISL_17964857	EPI_ISL_17964857	EPI_ISL_17964857	EPI_ISL_17964857	EPI_ISL_17964857		USA
A/Red-Tailed_Hawk/NS/FAV-0238-1/2023	EPI_ISL_19775602	EPI_ISL_19775602	EPI_ISL_19775602	EPI_ISL_19775602	EPI_ISL_19775602	EPI_ISL_19775602	EPI_ISL_19775602	EPI_ISL_19775602	USA
A/Red-Tailed_Hawk/PE/FAV-0222-3/2023	EPI_ISL_19775628	EPI_ISL_19775628	EPI_ISL_19775628	EPI_ISL_19775628	EPI_ISL_19775628	EPI_ISL_19775628	EPI_ISL_19775628	EPI_ISL_19775628	USA
A/Ross_s_Goose/North_Dakota/USDA-011033-001/2022	EPI_ISL_18132490	EPI_ISL_18132490	EPI_ISL_18132490	EPI_ISL_18132490		EPI_ISL_18132490	EPI_ISL_18132490	EPI_ISL_18132490	USA
A/royal_tern/Argentina/CH-PD036/2023	EPI_ISL_19466181	EPI_ISL_19466181		EPI_ISL_19466181	EPI_ISL_19466181	EPI_ISL_19466181		EPI_ISL_19466181	Argentina
A/Sanderling/Arica_y_Parinacota/230758-1/2022	EPI_ISL_17885980	EPI_ISL_17885980	EPI_ISL_17885980	EPI_ISL_17885980		EPI_ISL_17885980	EPI_ISL_17885980		Chile

Supplementary Figure S1. Maximum likelihood phylogenetic trees inferred independently for each of the eight influenza A virus genome segments: PB2, PB1, PA, HA, NP, NA, MP, and NS. Genotype assignment is indicated by shaded background colors: A, B1.3, B2.2, and B3.2.

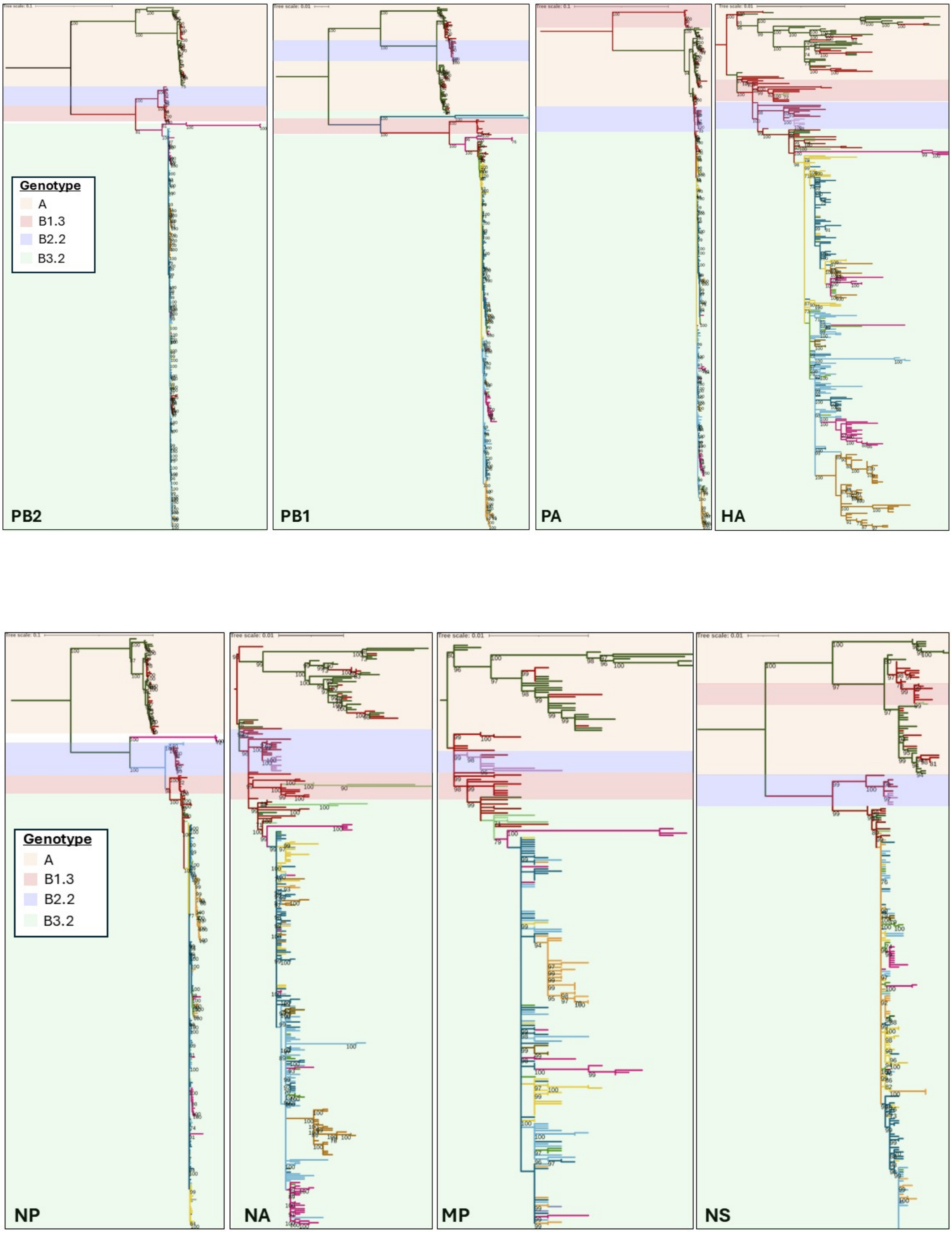

Supplement: Supplementary file 1 [file viruses-18-00764-s001.zip › viruses-4377927-supplementary.pdf]
